# Genome-Wide Small RNA Sequencing and Gene Expression Analysis Reveals a microRNA Profile of Cancer Susceptibility in ATM-Deficient Human Mammary Epithelial Cells

**DOI:** 10.1371/journal.pone.0064779

**Published:** 2013-05-31

**Authors:** Jill E. Hesse, Liwen Liu, Cynthia L. Innes, Yuxia Cui, Stela S. Palii, Richard S. Paules

**Affiliations:** 1 Environmental Stress and Cancer Group, Laboratory of Toxicology and Pharmacology, National Institute of Environmental Health Sciences, Research Triangle Park, North Carolina, United States of America; 2 NIEHS Microarray Core, Laboratory of Toxicology and Pharmacology, National Institute of Environmental Health Sciences, Research Triangle Park, North Carolina, United States of America; H. Lee Moffitt Cancer Center & Research Institute, United States of America

## Abstract

Deficiencies in the ATM gene are the underlying cause for ataxia telangiectasia, a syndrome characterized by neurological, motor and immunological defects, and a predisposition to cancer. MicroRNAs (miRNAs) are useful tools for cancer profiling and prediction of therapeutic responses to clinical regimens. We investigated the consequences of ATM deficiency on miRNA expression and associated gene expression in normal human mammary epithelial cells (HME-CCs). We identified 81 significantly differentially expressed miRNAs in ATM-deficient HME-CCs using small RNA sequencing. Many of these have been implicated in tumorigenesis and proliferation and include down-regulated tumor suppressor miRNAs, such as hsa-miR-29c and hsa-miR-16, as well as over-expressed pro-oncogenic miRNAs, such as hsa-miR-93 and hsa-miR-221. MicroRNA changes were integrated with genome wide gene expression profiles to investigate possible miRNA targets. Predicted mRNA targets of the miRNAs significantly regulated after ATM depletion included many genes associated with cancer formation and progression, such as SOCS1 and the proto-oncogene MAF. While a number of miRNAs have been reported as altered in cancerous cells, there is little understanding as to how these small RNAs might be driving cancer formation or how they might be used as biomarkers for cancer susceptibility. This study provides preliminary data for defining miRNA profiles that may be used as prognostic or predictive biomarkers for breast cancer. Our integrated analysis of miRNA and mRNA expression allows us to gain a better understanding of the signaling involved in breast cancer predisposition and suggests a mechanism for the breast cancer-prone phenotype seen in ATM-deficient patients.

## Introduction

The ataxia telangiectasia mutated (ATM) protein plays a critical role in a vast array of cellular responses including cell cycle regulation, apoptosis, and DNA damage responses. Individuals with complete deficiencies in ATM suffer from severe ataxia, immunological disorders, and have elevated risk for developing lymphoproliferative cancers [1]. In addition to complete loss of ATM functionality, it is estimated that as many as 1% of the United States population carry one mutated copy of ATM [2], [3] and are subject to the consequences of haploinsufficiency. While heterozygous carriers do not suffer from ataxia telangiectasia syndrome, they have an increased risk of developing heart disease, diabetes, and cancers, specifically breast cancer, compared to individuals with normal ATM expression levels [2], [3]. More recent epidemiological studies have shown that ATM mutations, which cause ataxia telangiectasia in the homozygous state, are also breast cancer susceptibility alleles in heterozygous carriers [4], [5], [6], [7], [8], [9], [10]. Additionally, it has been implicated that epigenetic silencing of ATM through methylation may also play a role in breast cancer susceptibility [11], [12]. Despite this link between reduced ATM levels and breast cancer, deficiencies in the ATM protein are not frequently observed in non-familial breast cancer populations [13]. This observation suggests the possibility that signaling pathways or gene expression patterns that are under ATM control might be a plausible mechanism linking ATM expression status to breast cancer predisposition. Here we propose ATM-dependent changes in epigenetic regulation, in particular miRNA regulation of gene expression, play a role in the formation of breast cancer.

MicroRNAs (miRNA) are a large regulatory class of small RNAs that have been described to post-transcriptionally modify gene expression. The high level of conservation in miRNA sequences across species emphasizes their important regulatory role. The primary mode of action for miRNA regulation of gene expression is binding to the 3′UTR region of the messenger RNA (mRNA), leading to a decrease in the amount of mRNA available in the cell, through either RNA degradation or prevention of translation. MicroRNAs play critical roles in development, differentiation and disease progression, and are predicted to target more than 60% of mRNAs in humans [14]. More recently, miRNAs have been used in the diagnosis, identification and prediction of therapeutic responses of cancers [15], [16], [17], [18], [19], [20]. MicroRNA profiling of different types of cancer has been used to identify cancer-causing miRNAs, termed oncomirs, as well as tumor suppressor miRNAs [20], [21], [22]. Many different groups have generated miRNA profiles in breast cancer cells in order to develop potential biomarkers in diagnosis and treatment [23]. Several miRNAs appear in many of the miRNA profiling experiments as being deregulated in breast cancer. However, the majority of these studies were conducted in cancerous cells and tumors. Here we investigate the role of ATM on the expression levels and composition of miRNAs in normal non-cancerous human mammary epithelial cells (HME-CC). We are interested in understanding how changes in miRNA expression due to ATM deficiency may contribute to cancer predisposition and elicit alterations in normal physiological pathways. Utilizing Illumina Next-Generation Sequencing, we performed genome-wide sequencing of small RNAs from normal wild type (WT) and ATM-deficient HME-CCs. In addition to deep sequencing of the small RNAs, we performed whole genome gene expression analysis using Agilent whole genome microarrays. The combination of both gene expression and miRNA profiles allowed us to identify possible gene targets for significantly regulated miRNAs. By understanding the composition and expression of miRNAs in ATM-deficient mammary epithelial cells we hope to gain insight into the mechanisms and underlying physiology through which breast cancer tumor formation proceeds.

## Materials and Methods

### Cell Culture

The HME-CC-LacZ (wild type) and HME-CC-ATM1 (ATM-deficient) human mammary epithelial cell lines were derived as described [24]. Cells were grown and maintained in HuMEC Ready Media (Invitrogen 12752010) with 4 µg/mL blasticidin (Invitrogen 46–1120).

### Cell Harvest and RNA Extraction

Wild type and ATM-deficient human mammary epithelial logarithmically growing cells were harvested using 0.25% trypsin-EDTA (Invitrogen 25200072), which was neutralized using TNS (Lonza CC-5002). Total RNA was isolated using the Ambion mirVana miRNA isolation kit using the standard protocol for total RNA isolation. The isolated total RNA was then split in aliquots so that the same RNA was used for small RNA sequencing and for gene expression analysis.

### Illumina Small RNA-sequencing

Triplicate biological samples of total RNA were submitted to the NIH Intramural Sequencing Center. Libraries of Small RNA cDNA were created using the Illumina Small RNA Sample Preparation Alternative v1.5 Protocol with only small deviations from the manufacturer’s protocol. These cDNA libraries were then sequenced on the Illumina Genome Analyzer IIX with 35 base pair reads according to the manufacturer’s instructions. Each library was run on a single lane in the flow cell with 36 cycles using Illumina version 5 chemistry. After alignment to the genome, read counts were normalized calculating miRNAs per million miRNA alignments. The list of significantly regulated miRNAs was created using a t-test analysis (p≤0.05) and fold change (≥1.5) cut-off between ATM-deficient cells compared to wild type cells.

### Agilent Whole Genome Array

Isolated total RNA was submitted to the NIEHS Microarray Core facility for microarray analysis. Gene expression analysis was conducted using Agilent Whole Human Genome 4×44 multiplex format oligo arrays (Agilent Technologies, 014850) following the Agilent 1-color microarray-based gene expression analysis protocol. Starting with 500 ng of total RNA, Cy3 labeled cRNA was produced according to the manufacturer’s protocol. For each sample, 1.65 ug of Cy3 labeled cRNAs were fragmented and hybridized for 17 hours in a rotating hybridization oven. Slides were washed and then scanned with an Agilent Scanner. Data was obtained using the Agilent Feature Extraction software (v9.5), using the 1-color defaults for all parameters. The Agilent Feature Extraction Software performed error modeling, adjusting for additive and multiplicative noise. The resulting data were processed and analyzed using Partek Genomics Suite (Partek® Genomics Suite software, version 6.6beta, Copyright © 2009, Partek Inc., St. Louis, MO, USA). A t-test was performed between the wild type and ATM-deficient samples, generating associated *p*-values. Combined with a fold change of +/−1.5, a *p*-value of ≤0.05 was used to generate a list of differentially expressed genes.

### Integration of MicroRNA and Gene Expression Data

Using TargetScan 5.2 [14], [25], [26], a list of biologically predicted targets of our differentially expressed miRNAs was determined. That list of targets was then compared to our list of differentially expressed genes to determine possible miRNA – mRNA interactions.

### Functions Analyses in Ingenuity

Significantly regulated miRNAs and mRNAs were analyzed with Ingenuity Pathway Analysis (IPA) [25], [26]. Networks and functional analyses were generated based on the 40 significant miRNAs and 202 significant predicted gene targets annotated in IPA. A right‐tailed Fisher’s exact test was used to calculate a p‐value determining the probability that each biological function and/or disease assigned to that data set is due to chance alone. Functions with a *p*-value of less than 0.01 were considered significant.

### Data Access

Data from microarrays and Small RNA sequencing used in this study have been archived at the Gene Expression Omnibus (GEO) database (http://www.ncbi.nlm.nih.gov/geo/).

The GEO accession number for the small RNA sequencing is GSE36267 and can be reviewed at http://www.ncbi.nlm.nih.gov/geo/query/acc.cgi?token=jxezrakumucekru&acc=GSE36267.

The GEO accession number for the Agilent whole genome expression array data is GSE36082 ad can be reviewed at http://www.ncbi.nlm.nih.gov/geo/query/acc.cgi?token=jvixzkwsaqiqwps&acc=GSE36082.

Accompanying Supplemental data consists of 7 tables that can be found online.

## Results

### Sequencing and Data Analysis

**Figure 1:**
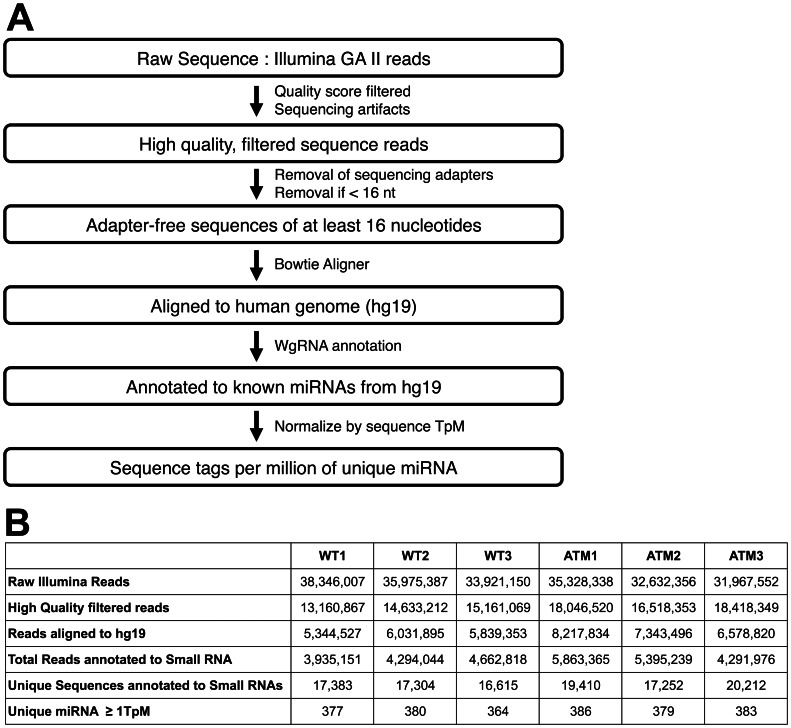
Process Map and Summary of Next Gen Sequencing data. A) Small RNA Sequencing pipeline overview. B) Summary statistics of Small RNA sequencing data at different stages of data analysis.

Illumina deep sequencing was used to generate small RNA reads from 3 biological replicates each of wild type and ATM-deficient non-cancerous HME-CCs. Filtering based on quality scores and sequencing artifacts allowed for selection of only robust sequence reads (Figure 1A). Using the FastX tool kit [27], the adaptor sequences were clipped from the 3′ end of the sequence reads and sequences with no adaptor sequence or with less than 16 nucleotides remaining after adaptor removal were excluded from further analysis. Clipped reads were aligned using Bowtie [28] to the hg19 human genome build. We allowed no mismatches in the 15bp seed region of the sequence reads and allowed up to 3 alignments per sequence read. Alignments were annotated with the UCSC small genome track (WgRNA) on hg19. Read counts were normalized by calculating tags per million miRNA alignments (TpM). Sequence filtering, adaptor clipping, and Bowtie alignment were performed using a combination of command line programs and the open web based platform Galaxy [29], [30], [31]. Summary statistics of all 6 samples were tracked to ensure similarity in the sequencing runs, alignments, and annotations (Figure1B).

### Composition of miRNA in Wild Type and ATM-deficient Cells

Next generation deep sequencing allows for a large dynamic range in detecting miRNAs (Table S1). When considering all of our samples, we identified 390 miRNAs present in two out of three samples in either the wild type or ATM-deficient samples with at least 1 TpM (Figure 1B). To restrict our analysis to only the most robust miRNAs, we considered only miRNAs with at least 10 TpM in two out of three samples in either the wild type or ATM-deficient samples in further analysis. 259 miRNAs were considered present in all samples and moved to the next stage of analysis (Table S2). Sequence tags per million miRNA alignments (TpM) were imported into Partek Genomics Suite for further analysis (Partek® Genomics Suite software, version 6.6beta, Copyright © 2009 Partek Inc., St. Louis, MO, USA). A precursory look at the expression levels of the 259 miRNAs highlights the broad dynamic range of expression detected by next generation deep sequencing. We were able to detect miRNAs with only a few transcripts as well as miRNAs with robust expression, such as hsa-miR-21, which has an average WT expression of 113,949 TpM. A high level review of the preliminary miRNA profile reveals specific effects of the depletion of ATM on expression. We observed not only miRNAs who’s expression was affected by the loss of ATM, but we were also able to identify miRNAs that appear to be unaffected by ATM depletion.

### Changes in Expression Profiles of miRNAs after Depletion of ATM

**Figure 2:**
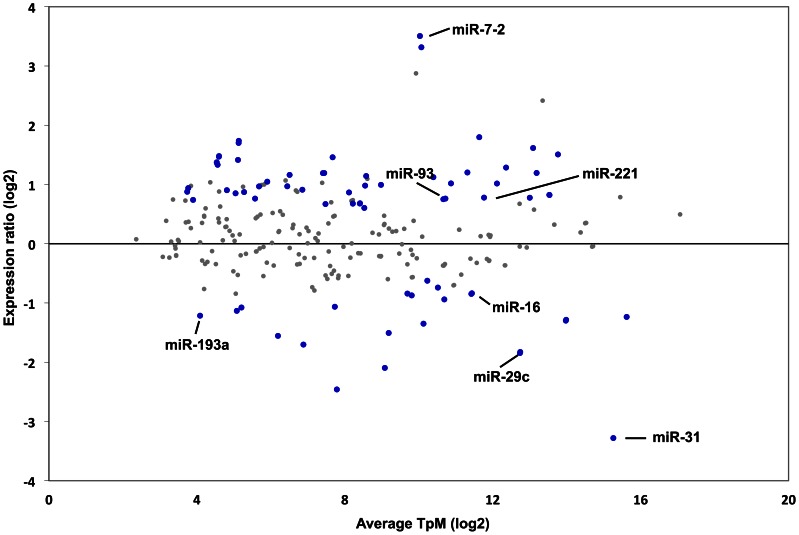
Differential expression of 259 present miRNAs in both wild type and ATM-deficient HME-CCs. Differential miRNA expression between wild type and ATM-deficient HME-CCs obtained from three independent replicates of each. The *y*-axis displays the ATM-deficient to WT expression ratio, the *x*-axis displays the average expression of each miRNA; both axes are in logarithmic scale. Differentially expressed miRNAs of *p*-value ≤0.05 and at least 1.5 fold change are blue. Representative significant miRNAs are labelled. Each sample had a separately generated sequencing library and was run in an individual sequencing lane.

**Figure 3:**
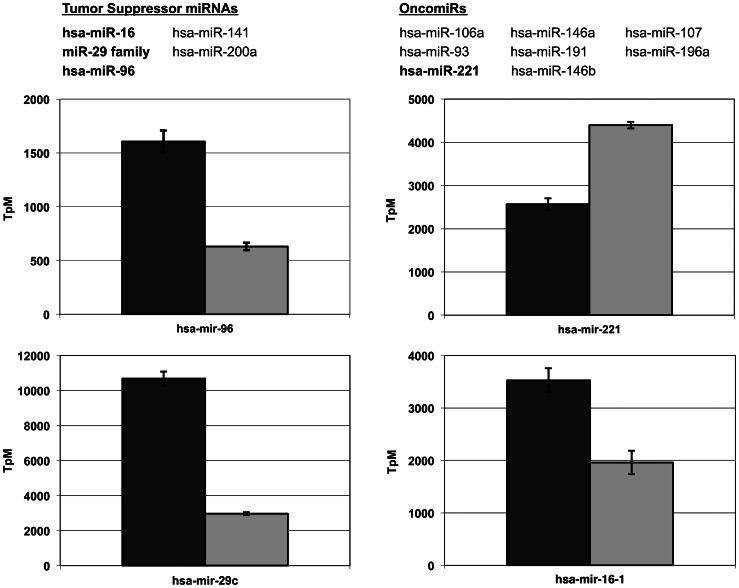
Depletion of ATM leads to deregulation of miRNAs important in cancer formation. A) List of 4 known tumor suppressors and 8 oncomirs with significant expression changes after the depletion of ATM. B) Expression levels, tags per million (TpM), of four examples of deregulated miRNAs. Dark gray bars represent wild type expression and light gray bars represent expression in ATM-deficient cells. Error bars are standard error from the expression of 3 independent replicates of each genotype.

To evaluate the magnitude and significance of the change in miRNA expression after the depletion of ATM, we conducted a t-test on 259 miRNAs considered present in both the wild type and ATM-deficient samples. Associated *p-*values were calculated and combined with a fold change to generate a list of significantly differentially regulated (*p*≤0.05; fold change ≥1.5) miRNAs. This identified 81 miRNAs as significantly differentially expressed between the two HME-CC genotypes (Figure 2 & Table S3), thus more than 30% of the miRNA considered present in our analysis are significantly differentially regulated after the depletion of ATM. We found several well characterized miRNAs that were statistically unchanged after the depletion of ATM, including the let-7 family and hsa-miR-21. Among the 81 significantly regulated miRNAs, there are 55 miRNAs with higher levels of expression and 26 with lower levels of expression as compared to the wild type HME-CCs. Interestingly, these regulated miRNAs include several that have been implicated in cancer formation or metastasis. Utilizing the TAM tool for annotating miRNAs [32] and the human miRNA disease database [33], we identified 4 repressed miRNA tumor suppressors and 7 over-expressed oncomirs [21], [22], [34] in the ATM-deficient cells (for examples, see Figure 3). This observation of deregulation of miRNAs important in cancer formation or suppression suggests ATM-dependent miRNA expression changes may alter biological pathways and functionalities that promote cancer formation. Based on miRNA cancer profiling projects, several ATM-dependent miRNAs merited closer examination (Figure 3). For example, loss of ATM results in a decreased expression of the tumor suppressive miRNAs, miR- 96, which is known to target KRAS [35], and the miR-29 family, which includes miR-29b-1, miR-29b-2, and miR-29c. A decrease in expression of all three members of the miR-29 family has been implicated in many types of cancer [36] and has a correlation with prognosis [37]. Additionally, loss of ATM results in an increase in expression of miR-10b, which is highly expressed in metastatic breast cancers [38], [39] and miR-221, which can induce proliferation and cell survival [40]. In order to further understand the biological effects of the miRNA expression changes, we undertook whole genome gene expression analysis.

### Expression of Genes Predicted to be miRNA Targets

Using the Agilent human whole genome microarray, we gathered gene expression data from the same WT and ATM-deficient HME-CC samples. With a combination of *p*-value (≤0.05) and fold change (≥1.5) cut-offs, we identified 1086 significantly changed probes representing 988 genes or genetic areas (Table S4). In order to identify genes possibly targeted by changes in miRNA expression, we utilized TargetScan 5.2 to predict mRNA targets of the 259 significantly regulated miRNAs. We limited the predicted mRNA targets, first based on genes significantly regulated in our gene expression analysis and second on targets either experimentally proven or highly predicted by TargetScan. Additionally, based on the knowledge that miRNAs predominately regulate gene expression through an inhibition of expression, we only retained miRNA-mRNA combinations that are inversely correlated, recognizing we may be excluding functionally important regulatory interactions of mRNAs. Beginning with our 81 significantly regulated miRNAs, we identified 40 that had highly predicted mRNA targets with significant changes in associated gene expression. We identified 202 significantly regulated mRNA targets that were inversely correlated with the miRNAs predicted to target them (Table S5). Many of the miRNAs are predicted to target multiple significantly regulated mRNAs and many of the mRNAs are potentially targeted by more than one ATM-dependent miRNA. These phenomena might suggest functional redundancy in the miRNA regulation of target genes.

**Figure 4:**
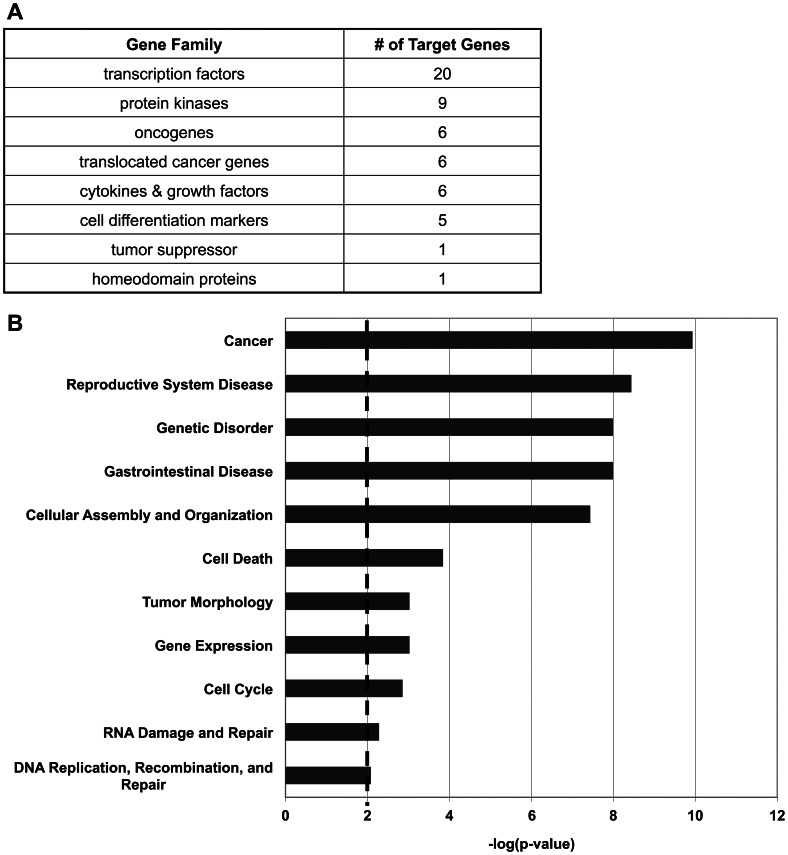
Depletion of ATM in non-cancerous cells reveals effects on miRNAs and target mRNAs that suggest an early event in transformation to cancer. A) Gene Families representing the 202 significantly regulated genes determined using GSEA to give a functional overview of the types of genes affected by changes in miRNA expression. B) Top Functions analysis of ATM-dependent correlated miRNAs and possible mRNA targets by IPA. Only selected significant functional groups are depicted. The dashed line indicates a *p*-value of 0.01.

Using Gene Set Enrichment Analysis (GSEA) [41], we determined the gene families represented by the genes predicted to be targeted by miRNAs in an ATM-dependent manner (Figure 4A). This approach gives a functional overview of the genes in our list using the Molecular Signatures Database [41]. Gene families share common features such as biochemical activity and homology. Interestingly, many of the predicted target genes of the significantly regulated miRNAs are transcription factors (shown in Table S6), suggesting that many of the ATM-dependent miRNAs may control a large phenotypic response based on the regulation of transcription factor expression. The involvement of miRNA regulation of transcription factors has been predicted and modeled in several different types of cancer [42], [43], [44]. Our findings show changes in miRNAs as well as the alteration of transcription factor expression that is occurring after the loss of ATM. Additionally, our findings suggest this alteration of transcription factors may be occurring before the cells become malignant (Table S6). For example, increased expression of oncogenic transcription factors such as MAF and CEBPA indicate miRNAs may regulate large networks of genes involved in cancer formation. Furthermore, 6 oncogenes and one known tumor suppressor, SOCS1, are predicted to be miRNA targets. This is consistent with previous reports suggesting miRNAs can play a critical role in the formation and progression in cancer. Understanding the early effects of miRNAs in the regulation of transcription factors and cancer formation may lead to insight into prognostic and preventative treatments.

### Pathway/functional Analysis of Predicted Targets

In order to gain additional insight into the biological functions and networks being affected by the ATM–dependent changes in miRNA expression, we utilized Ingenuity Pathway Analysis (IPA) [45]. Functional analysis and network generation analysis through IPA allows us to examine all the potential roles of significantly changed miRNAs and their predicted gene targets by mining the IPA Knowledge Base to identify connectivity and relationships between our genes and miRNAs of interest. Five networks were significantly affected after the depletion of ATM, including cellular functions of morphology, cell cycle, cell growth and proliferation. At least 76 large functions groups were significantly (p<0.01) affected after the depletion of ATM, with several cancer and tumor specific functions being highly affected (Figure 4B). The most significantly affected biological function was cancer (*p*<1×10̂10), with other significantly affected functions including tumor morphology, DNA damage and repair, and cell death. (Table S7 shows representative genes in several of these functional groups.) The deregulation of these biological functions in non-cancerous cells suggests the loss of ATM, with associated changes in miRNA expression levels, may predispose or drive the early stages of cancer formation.

## Discussion

Through genome-wide deep sequencing we have gained information about essentially all the small RNAs present in both wild type and ATM-deficient normal human mammary epithelial cells, not just those that are highly expressed. We identified 259 robustly expressed miRNAs present in both wild type and ATM-deficient normal human mammary epithelial cells. While ATM has been implicated in the biogenesis of miRNAs after DNA damage [46], a full investigation of the effect of ATM depletion in HME-CCs reveals that the depletion of ATM itself has both the expected down-regulation of miRNAs as well as a significant number of up-regulated miRNAs. Only one-third of the significantly regulated miRNAs have decreased expression after the depletion of ATM. Based on the presence of up-regulated miRNAs following ATM depletion, we speculate that ATM’s role in effecting miRNA expression is not limited to miRNA biogenesis and could be occurring via multiple mechanisms. Additionally, loss of ATM has been associated with an increased level of oxidative stress [47], [48]. We speculate the higher level of oxidative stress triggered by the constitutive loss of ATM may have an effect on the expression levels of many miRNAs. The depletion, or loss, of ATM may be driving changes in miRNA expression through the increase in oxidative stress. Several miRNAs seen to have altered expression after the depletion of ATM have also been shown to be regulated by elevated oxidative stress, such as hsa-miR-200c [49]. Additional studies are needed to determine whether reactive oxygen species scavengers can mitigate the miRNA changes or phenotype changes seen with the depletion or loss of ATM.

When we compared the miRNA expression between the wild type and ATM-deficient HME-CCs we discovered that the miRNAs dis-regulated after the depletion of ATM show an unusually high correlation with miRNAs implicated in cancer. Of the 81 significantly regulated miRNAs, the depletion of ATM drove the repression of 4 known miRNA tumor suppressors and the over-expression of 7 known oncomirs. This initial observation suggests a means by which the loss of ATM might predispose cells to cancer or increase cancer-susceptibility.

To further investigate the role of the miRNAs in the ATM-deficient cells, we combined the significantly regulated miRNA and gene expression data to predict and evaluate possible miRNA-mRNA target combinations. Approximately half of the significantly regulated miRNAs had predicted targets that were also differentially regulated, as seen in our gene expression analysis. When we further investigated these miRNA and possible targets we highlighted additional evidence of a cancer-susceptibility profile in ATM-deficient human mammary epithelial cells. This includes the increased regulation of oncogenes MAF and CEPBA as well as the decreased regulation of the tumor suppressor SOCS1.

Understanding the genome-wide miRNA profiles of normal and ATM-deficient non-cancerous mammary epithelial cells can help us gain a better understanding of the roles of ATM and miRNAs in the tumorigenesis of breast cancer and the transition from non-cancerous to malignant breast tissue.

Of particular interest is the possibility of utilizing miRNA expression levels to predict cancer susceptibility or formation before it can be detected by conventional diagnostic methods. The cells in our study are normal human mammary epithelial cells that are not derived from cancerous cells. Therefore, the increased expression of oncomirs and the repression of some tumor suppressor miRNAs in ATM-deficient cells suggest that it may be possible to develop biomarkers for breast cancer predisposition. Utilizing miRNA biomarker profiles consistent with breast cancer predisposition would allow early identification of patients who are at risk and perhaps lead to earlier monitoring, detection, and treatment.

### Conclusion

In conclusion, 81 miRNAs with ATM-dependent expression have been identified. These miRNAs, along with their putative mRNA targets, suggest they may play a role in the early stages of transition from normal to cancerous mammary epithelial cells. This study provides the preliminary data to suggest that combining microRNA and mRNA expression can be of value for developing a biomarker for predicting predisposition to breast cancer. By identifying cells and patients with an increased potential for breast cancer formation, recommendations can be made involving monitoring and early intervention.

## Supporting Information

Table S1 **Sequence count for all 939 annotated miRNAs.** Tags per million (TpM) sequence count for all 939 microRNAs annotated in the hg19 WgRNA track of the UCSC Genome browser.
(PDF)

Table S2 **Sequence count for 259 present miRNAs.** Sequence count for the 259 miRNAs deemed present (above 10 TpM) in either 2 out of 3 wild type replicates or 2 out of 3 ATM-deficient replicates.
(PDF)

Table S3 **81 ATM-dependent miRNAs.** 81 microRNAs determined to have a significant change in expression in the ATM-deficient cells compared to wild type controls. T-test *p*≤0.05; Fold Change +/−1.5 or greater.
(PDF)

Table S4 **1086 ATM-dependent mRNAs.** 1086 mRNA probes determined to have significant expression changes in ATM-deficient cells compared to wild-type control cells. T-test *p*≤0.05; Fold Change +/−1.5 or greater.
(PDF)

Table S5 **40 miRNA and 202 mRNA targets.** List of 40 significantly regulated miRNAs with negatively correlated predicted mRNA targets and the 202 mRNA predicted targets with significant changes in gene expression. The direction of change (increase or decrease) of the ATM-deficient cells compared to the WT cells is indicated for both the miRNAs (column 2) and the gene (mRNA) targets (column 4).
(PDF)

Table S6 **Potential target transcription factors.** Transcription Factors identified as possible targets of significantly regulated miRNAs using the Molecular Signatures Database of GSEA.
(PDF)

Table S7 **Representative genes in specific functional categories.** Examples of target genes implicated in Cancer, Cell Cycle Regulation and DNA Replication, Recombination and Repair based on Ingenuity Pathway Analysis.
(PDF)
