# Supplementary material for: Genome-Wide Small RNA Sequencing and Gene Expression Analysis Reveals a microRNA Profile of Cancer Susceptibility in ATM-Deficient Human Mammary Epithelial Cells
Source: PLoS One. 2013 May 31;8(5):e64779. doi: 10.1371/journal.pone.0064779 (PMC3669333; doi:10.1371/journal.pone.0064779)
Supplement: Table S1 — Sequence count for all 939 annotated miRNAs. Tags per million (TpM) sequence count for all 939 microRNAs annotated in the hg19 WgRNA track of the UCSC Genome browser. (PDF) [file pone.0064779.s001.pdf]

| miRNA ID | WT1-TpM | WT2- TpM | WT3- TpM | ATM1- TpM | ATM2- TpM | ATM3- TpM |
|---|---|---|---|---|---|---|
| hsa-let-7a-1 | 21315 | 20001 | 20740 | 26721 | 27131 | 25236 |
| hsa-let-7a-2 | 21109 | 19815 | 20514 | 26499 | 26938 | 24799 |
| hsa-let-7a-3 | 21371 | 20045 | 20774 | 26817 | 27194 | 25335 |
| hsa-let-7b | 21264 | 19975 | 20654 | 26757 | 27102 | 24962 |
| hsa-let-7c | 21011 | 19761 | 20450 | 26440 | 26902 | 24473 |
| hsa-let-7d | 1079 | 1125 | 1009 | 968 | 1121 | 620 |
| hsa-let-7e | 1260 | 1108 | 774 | 1219 | 1552 | 641 |
| hsa-let-7f-1 | 20941 | 20208 | 18522 | 22124 | 29249 | 16706 |
| hsa-let-7f-2 | 20903 | 20183 | 18486 | 22093 | 29241 | 16649 |
| hsa-let-7g | 5775 | 6073 | 5625 | 4827 | 5460 | 3276 |
| hsa-let-7i | 5730 | 6061 | 5584 | 4832 | 5468 | 3188 |
| hsa-mir-1-1 | 0 | 0 | 0 | 0 | 0 | 0 |
| hsa-mir-1-2 | 0 | 0 | 0 | 1 | 0 | 0 |
| hsa-mir-100 | 4103 | 3753 | 3364 | 4165 | 2613 | 5374 |
| hsa-mir-101-1 | 5597 | 5718 | 5707 | 12808 | 12117 | 14022 |
| hsa-mir-101-2 | 5595 | 5716 | 5705 | 12807 | 12116 | 14022 |
| hsa-mir-103-1 | 9863 | 8154 | 7631 | 14930 | 14362 | 16131 |
| hsa-mir-103-1-as | 9863 | 8154 | 7631 | 14930 | 14362 | 16131 |
| hsa-mir-103-2 | 9899 | 8193 | 7643 | 14952 | 14385 | 16147 |
| hsa-mir-103-2-as | 9878 | 8170 | 7638 | 14944 | 14376 | 16141 |
| hsa-mir-105-1 | 0 | 0 | 0 | 0 | 0 | 0 |
| hsa-mir-105-2 | 0 | 0 | 0 | 0 | 0 | 0 |
| hsa-mir-106a | 19 | 19 | 19 | 50 | 44 | 57 |
| hsa-mir-106b | 3827 | 3089 | 2445 | 3195 | 2240 | 4787 |
| hsa-mir-107 | 9857 | 8157 | 7624 | 14928 | 14363 | 16078 |
| hsa-mir-10a | 129 | 122 | 132 | 136 | 85 | 164 |
| hsa-mir-10b | 58 | 57 | 62 | 124 | 121 | 102 |
| hsa-mir-1178 | 0 | 0 | 0 | 0 | 0 | 0 |
| hsa-mir-1179 | 0 | 0 | 0 | 0 | 0 | 0 |
| hsa-mir-1180 | 3 | 6 | 6 | 7 | 8 | 10 |
| hsa-mir-1181 | 0 | 0 | 0 | 0 | 0 | 0 |
| hsa-mir-1182 | 0 | 0 | 0 | 0 | 0 | 0 |
| hsa-mir-1183 | 0 | 0 | 0 | 0 | 0 | 0 |
| hsa-mir-1184-1 | 0 | 0 | 0 | 0 | 0 | 0 |
| hsa-mir-1184-2 | 0 | 0 | 0 | 0 | 0 | 0 |
| hsa-mir-1184-3 | 0 | 0 | 0 | 0 | 0 | 0 |
| hsa-mir-1185-1 | 1 | 0 | 0 | 0 | 0 | 0 |
| hsa-mir-1185-2 | 1 | 0 | 0 | 0 | 0 | 0 |
| hsa-mir-1193 | 0 | 0 | 0 | 0 | 0 | 0 |
| hsa-mir-1197 | 0 | 0 | 0 | 0 | 0 | 0 |
| hsa-mir-1200 | 0 | 0 | 0 | 0 | 0 | 0 |
| hsa-mir-1201 | 31 | 38 | 37 | 57 | 47 | 75 |
| hsa-mir-1202 | 0 | 0 | 0 | 0 | 0 | 0 |
| hsa-mir-1203 | 0 | 0 | 0 | 0 | 0 | 0 |
| hsa-mir-1204 | 0 | 0 | 0 | 0 | 0 | 0 |
| hsa-mir-1205 | 0 | 0 | 0 | 0 | 0 | 0 |
| hsa-mir-1206 | 0 | 0 | 0 | 0 | 0 | 0 |
| hsa-mir-1207 | 0 | 0 | 0 | 0 | 0 | 0 |
| hsa-mir-1208 | 0 | 0 | 0 | 0 | 0 | 0 |
| hsa-mir-122 | 0 | 0 | 0 | 0 | 0 | 0 |
| hsa-mir-1224 | 0 | 0 | 0 | 0 | 0 | 0 |
| hsa-mir-1225 | 0 | 0 | 0 | 0 | 0 | 0 |

| | | | | | | |
|---|---|---|---|---|---|---|
| hsa-mir-1226 | 2 | 3 | 2 | 3 | 2 | 2 |
| hsa-mir-1227 | 0 | 1 | 1 | 1 | 1 | 0 |
| hsa-mir-1228 | 0 | 0 | 0 | 0 | 1 | 0 |
| hsa-mir-1229 | 0 | 0 | 0 | 0 | 1 | 0 |
| hsa-mir-1231 | 0 | 0 | 0 | 0 | 0 | 0 |
| hsa-mir-1233-1 | 0 | 0 | 0 | 0 | 0 | 0 |
| hsa-mir-1233-2 | 0 | 0 | 0 | 0 | 0 | 0 |
| hsa-mir-1234 | 0 | 0 | 0 | 0 | 0 | 0 |
| hsa-mir-1236 | 0 | 0 | 0 | 0 | 0 | 0 |
| hsa-mir-1237 | 0 | 1 | 0 | 1 | 1 | 1 |
| hsa-mir-1238 | 0 | 0 | 0 | 0 | 0 | 0 |
| hsa-mir-124-1 | 1 | 0 | 0 | 0 | 0 | 0 |
| hsa-mir-124-2 | 1 | 0 | 0 | 0 | 0 | 0 |
| hsa-mir-124-3 | 1 | 0 | 0 | 0 | 0 | 0 |
| hsa-mir-1243 | 0 | 0 | 0 | 0 | 0 | 0 |
| hsa-mir-1244-1 | 0 | 0 | 0 | 1 | 1 | 0 |
| hsa-mir-1244-2 | 0 | 0 | 0 | 1 | 1 | 0 |
| hsa-mir-1244-3 | 0 | 0 | 0 | 1 | 1 | 0 |
| hsa-mir-1245 | 0 | 0 | 0 | 0 | 0 | 0 |
| hsa-mir-1246 | 83 | 55 | 26 | 56 | 92 | 86 |
| hsa-mir-1247 | 0 | 0 | 0 | 0 | 0 | 0 |
| hsa-mir-1248 | 2 | 2 | 1 | 6 | 4 | 4 |
| hsa-mir-1249 | 1 | 2 | 3 | 2 | 4 | 3 |
| hsa-mir-1250 | 0 | 0 | 0 | 0 | 0 | 0 |
| hsa-mir-1251 | 0 | 0 | 0 | 0 | 0 | 0 |
| hsa-mir-1252 | 2 | 1 | 1 | 1 | 2 | 2 |
| hsa-mir-1253 | 0 | 0 | 0 | 0 | 0 | 0 |
| hsa-mir-1254 | 0 | 1 | 0 | 1 | 1 | 0 |
| hsa-mir-1255a | 13 | 8 | 8 | 11 | 13 | 8 |
| hsa-mir-1255b-1 | 1 | 0 | 0 | 1 | 1 | 0 |
| hsa-mir-1255b-2 | 1 | 0 | 0 | 0 | 1 | 0 |
| hsa-mir-1256 | 2 | 2 | 1 | 1 | 1 | 0 |
| hsa-mir-1257 | 0 | 0 | 0 | 1 | 1 | 0 |
| hsa-mir-1258 | 0 | 0 | 0 | 0 | 0 | 0 |
| hsa-mir-1259 | 223 | 214 | 197 | 124 | 76 | 237 |
| hsa-mir-125a | 206 | 336 | 331 | 297 | 266 | 313 |
| hsa-mir-125b-1 | 435 | 673 | 636 | 616 | 549 | 842 |
| hsa-mir-125b-2 | 378 | 611 | 612 | 589 | 514 | 808 |
| hsa-mir-126 | 23 | 27 | 27 | 18 | 17 | 25 |
| hsa-mir-1260 | 0 | 0 | 0 | 0 | 0 | 0 |
| hsa-mir-1260b | 35 | 48 | 43 | 48 | 39 | 86 |
| hsa-mir-1261 | 0 | 0 | 0 | 0 | 0 | 0 |
| hsa-mir-1262 | 2 | 2 | 2 | 5 | 7 | 3 |
| hsa-mir-1263 | 0 | 0 | 0 | 0 | 0 | 0 |
| hsa-mir-1264 | 0 | 0 | 0 | 0 | 0 | 0 |
| hsa-mir-1265 | 0 | 0 | 0 | 0 | 0 | 0 |
| hsa-mir-1266 | 2 | 2 | 2 | 7 | 5 | 5 |
| hsa-mir-1267 | 0 | 0 | 0 | 0 | 0 | 0 |
| hsa-mir-1268 | 19 | 32 | 10 | 22 | 35 | 26 |
| hsa-mir-1269 | 0 | 0 | 0 | 0 | 0 | 0 |
| hsa-mir-127 | 3 | 4 | 2 | 2 | 2 | 2 |
| hsa-mir-1270-1 | 3 | 3 | 2 | 4 | 4 | 4 |
| hsa-mir-1270-2 | 3 | 3 | 2 | 4 | 4 | 4 |

| | | | | | | |
|---|---|---|---|---|---|---|
| hsa-mir-1271 | 3 | 5 | 3 | 2 | 1 | 1 |
| hsa-mir-1272 | 0 | 0 | 0 | 0 | 0 | 0 |
| hsa-mir-1273 | 0 | 0 | 0 | 0 | 0 | 0 |
| hsa-mir-1273c | 2 | 1 | 1 | 1 | 1 | 1 |
| hsa-mir-1273d | 0 | 1 | 0 | 2 | 1 | 1 |
| hsa-mir-1274a | 12 | 8 | 6 | 4 | 2 | 6 |
| hsa-mir-1274b | 51 | 52 | 25 | 20 | 17 | 34 |
| hsa-mir-1275 | 47 | 38 | 38 | 56 | 39 | 72 |
| hsa-mir-1276 | 4 | 2 | 3 | 6 | 4 | 9 |
| hsa-mir-1277 | 118 | 128 | 82 | 30 | 34 | 48 |
| hsa-mir-1278 | 1 | 1 | 1 | 2 | 1 | 1 |
| hsa-mir-1279 | 0 | 0 | 0 | 0 | 0 | 0 |
| hsa-mir-128-1 | 77 | 72 | 59 | 84 | 81 | 76 |
| hsa-mir-128-2 | 77 | 72 | 59 | 83 | 79 | 75 |
| hsa-mir-1280 | 45 | 65 | 50 | 23 | 24 | 105 |
| hsa-mir-1281 | 0 | 0 | 0 | 0 | 0 | 0 |
| hsa-mir-1282 | 0 | 0 | 0 | 0 | 1 | 0 |
| hsa-mir-1283-1 | 0 | 0 | 0 | 0 | 0 | 0 |
| hsa-mir-1283-2 | 0 | 0 | 0 | 0 | 0 | 0 |
| hsa-mir-1284 | 1 | 1 | 1 | 0 | 1 | 1 |
| hsa-mir-1285-1 | 74 | 85 | 63 | 162 | 162 | 84 |
| hsa-mir-1285-2 | 71 | 83 | 61 | 159 | 160 | 81 |
| hsa-mir-1286 | 1 | 1 | 1 | 1 | 1 | 1 |
| hsa-mir-1287 | 0 | 1 | 0 | 1 | 1 | 1 |
| hsa-mir-1288 | 0 | 0 | 0 | 0 | 0 | 1 |
| hsa-mir-1289-1 | 0 | 0 | 0 | 1 | 0 | 1 |
| hsa-mir-1289-2 | 0 | 0 | 0 | 1 | 0 | 0 |
| hsa-mir-129-1 | 11 | 14 | 14 | 32 | 31 | 44 |
| hsa-mir-129-2 | 11 | 14 | 13 | 32 | 31 | 44 |
| hsa-mir-1290 | 0 | 0 | 0 | 0 | 0 | 0 |
| hsa-mir-1291 | 2 | 2 | 1 | 9 | 2 | 7 |
| hsa-mir-1292 | 11 | 13 | 9 | 24 | 16 | 17 |
| hsa-mir-1293 | 16 | 19 | 9 | 25 | 24 | 19 |
| hsa-mir-1294 | 1 | 1 | 1 | 1 | 1 | 0 |
| hsa-mir-1295 | 0 | 0 | 0 | 0 | 0 | 0 |
| hsa-mir-1296 | 28 | 56 | 39 | 33 | 32 | 20 |
| hsa-mir-1297 | 1136 | 1139 | 949 | 681 | 881 | 613 |
| hsa-mir-1298 | 0 | 0 | 0 | 0 | 0 | 0 |
| hsa-mir-1299 | 0 | 1 | 0 | 1 | 1 | 1 |
| hsa-mir-1301 | 2 | 3 | 1 | 5 | 4 | 3 |
| hsa-mir-1302-1 | 0 | 0 | 0 | 0 | 0 | 0 |
| hsa-mir-1302-10 | 0 | 0 | 0 | 0 | 0 | 0 |
| hsa-mir-1302-11 | 0 | 0 | 0 | 0 | 0 | 0 |
| hsa-mir-1302-2 | 0 | 0 | 0 | 0 | 0 | 0 |
| hsa-mir-1302-3 | 0 | 0 | 0 | 0 | 0 | 0 |
| hsa-mir-1302-4 | 0 | 0 | 0 | 0 | 0 | 0 |
| hsa-mir-1302-5 | 0 | 0 | 0 | 0 | 0 | 0 |
| hsa-mir-1302-6 | 0 | 0 | 0 | 0 | 0 | 0 |
| hsa-mir-1302-7 | 0 | 0 | 0 | 0 | 0 | 0 |
| hsa-mir-1302-8 | 0 | 0 | 0 | 0 | 0 | 0 |
| hsa-mir-1302-9 | 0 | 0 | 0 | 0 | 0 | 0 |
| hsa-mir-1303 | 6 | 6 | 3 | 5 | 5 | 4 |
| hsa-mir-1304 | 8 | 10 | 10 | 3 | 3 | 2 |

| | | | | | | |
|---|---|---|---|---|---|---|
| hsa-mir-1305 | 11 | 11 | 9 | 9 | 7 | 32 |
| hsa-mir-1306 | 7 | 8 | 6 | 4 | 4 | 3 |
| hsa-mir-1307 | 165 | 333 | 248 | 459 | 330 | 806 |
| hsa-mir-1308 | 1422 | 7661 | 804 | 13756 | 38192 | 797 |
| hsa-mir-130a | 1321 | 1670 | 1383 | 855 | 1132 | 851 |
| hsa-mir-130b | 328 | 382 | 264 | 180 | 356 | 132 |
| hsa-mir-132 | 31 | 42 | 23 | 38 | 30 | 32 |
| hsa-mir-1321 | 0 | 0 | 0 | 0 | 0 | 0 |
| hsa-mir-1322 | 0 | 0 | 0 | 0 | 0 | 0 |
| hsa-mir-1323 | 0 | 0 | 0 | 0 | 0 | 0 |
| hsa-mir-1324 | 0 | 0 | 0 | 0 | 0 | 0 |
| hsa-mir-133a-1 | 0 | 0 | 0 | 0 | 0 | 0 |
| hsa-mir-133a-2 | 0 | 0 | 0 | 0 | 0 | 0 |
| hsa-mir-133b | 0 | 0 | 0 | 0 | 0 | 0 |
| hsa-mir-134 | 1 | 1 | 1 | 1 | 2 | 2 |
| hsa-mir-135a-1 | 1 | 0 | 0 | 0 | 0 | 0 |
| hsa-mir-135a-2 | 1 | 0 | 0 | 0 | 0 | 0 |
| hsa-mir-135b | 1546 | 2136 | 1821 | 1119 | 1409 | 765 |
| hsa-mir-136 | 1 | 1 | 1 | 0 | 0 | 0 |
| hsa-mir-137 | 292 | 242 | 279 | 169 | 95 | 279 |
| hsa-mir-138-1 | 234 | 280 | 212 | 192 | 170 | 166 |
| hsa-mir-138-2 | 211 | 254 | 193 | 184 | 163 | 160 |
| hsa-mir-139 | 11 | 14 | 10 | 13 | 9 | 7 |
| hsa-mir-140 | 732 | 710 | 694 | 938 | 699 | 906 |
| hsa-mir-141 | 24560 | 23336 | 21130 | 8148 | 8210 | 11984 |
| hsa-mir-142 | 13 | 12 | 11 | 6 | 5 | 5 |
| hsa-mir-143 | 15 | 15 | 16 | 19 | 27 | 14 |
| hsa-mir-144 | 0 | 1 | 0 | 0 | 0 | 0 |
| hsa-mir-145 | 0 | 1 | 0 | 1 | 0 | 0 |
| hsa-mir-1468 | 0 | 0 | 0 | 0 | 0 | 0 |
| hsa-mir-1469 | 0 | 0 | 0 | 0 | 0 | 0 |
| hsa-mir-146a | 296 | 253 | 234 | 460 | 370 | 423 |
| hsa-mir-146b | 297 | 253 | 234 | 462 | 372 | 425 |
| hsa-mir-147 | 0 | 0 | 0 | 0 | 0 | 0 |
| hsa-mir-1470 | 0 | 0 | 0 | 0 | 0 | 0 |
| hsa-mir-1471 | 0 | 0 | 0 | 0 | 0 | 0 |
| hsa-mir-147b | 1 | 1 | 1 | 1 | 0 | 0 |
| hsa-mir-148a | 4112 | 3795 | 4989 | 13646 | 11379 | 14487 |
| hsa-mir-148b | 1474 | 1520 | 1272 | 5618 | 4907 | 4310 |
| hsa-mir-149 | 87 | 120 | 74 | 118 | 111 | 38 |
| hsa-mir-150 | 0 | 0 | 0 | 0 | 0 | 0 |
| hsa-mir-151 | 3156 | 2960 | 2693 | 6983 | 4951 | 5879 |
| hsa-mir-152 | 216 | 209 | 262 | 358 | 298 | 443 |
| hsa-mir-153-1 | 0 | 0 | 1 | 0 | 0 | 0 |
| hsa-mir-153-2 | 0 | 0 | 0 | 0 | 0 | 0 |
| hsa-mir-1537 | 0 | 0 | 0 | 0 | 0 | 0 |
| hsa-mir-1538 | 0 | 0 | 0 | 0 | 0 | 0 |
| hsa-mir-1539 | 0 | 0 | 0 | 0 | 0 | 0 |
| hsa-mir-154 | 1 | 1 | 1 | 0 | 0 | 1 |
| hsa-mir-155 | 1 | 1 | 1 | 1 | 0 | 1 |
| hsa-mir-15a | 1018 | 1068 | 828 | 762 | 819 | 977 |
| hsa-mir-15b | 676 | 868 | 711 | 680 | 827 | 502 |
| hsa-mir-16-1 | 2888 | 4248 | 3444 | 1932 | 2643 | 1306 |

| | | | | | | |
|---|---|---|---|---|---|---|
| hsa-mir-16-2 | 2918 | 4280 | 3467 | 1962 | 2676 | 1331 |
| hsa-mir-17 | 4238 | 4280 | 3488 | 3626 | 3013 | 3404 |
| hsa-mir-181a-1 | 438 | 485 | 508 | 611 | 431 | 760 |
| hsa-mir-181a-2 | 420 | 482 | 524 | 600 | 455 | 721 |
| hsa-mir-181b-1 | 223 | 241 | 206 | 332 | 271 | 513 |
| hsa-mir-181b-2 | 216 | 235 | 205 | 317 | 261 | 507 |
| hsa-mir-181c | 71 | 59 | 34 | 146 | 78 | 120 |
| hsa-mir-181d | 3 | 4 | 3 | 7 | 4 | 8 |
| hsa-mir-182 | 4673 | 5149 | 5857 | 11123 | 7641 | 6243 |
| hsa-mir-1825 | 0 | 0 | 0 | 0 | 0 | 0 |
| hsa-mir-1826 | 2675 | 3559 | 3891 | 2250 | 1903 | 3937 |
| hsa-mir-1827 | 5 | 5 | 4 | 8 | 10 | 7 |
| hsa-mir-183 | 821 | 880 | 843 | 2244 | 1716 | 1574 |
| hsa-mir-184 | 1 | 1 | 1 | 2 | 3 | 3 |
| hsa-mir-185 | 157 | 124 | 87 | 92 | 108 | 92 |
| hsa-mir-186 | 507 | 465 | 374 | 613 | 398 | 854 |
| hsa-mir-187 | 2 | 2 | 3 | 7 | 7 | 8 |
| hsa-mir-188 | 18 | 20 | 20 | 12 | 12 | 23 |
| hsa-mir-18a | 1997 | 1976 | 1545 | 1420 | 1095 | 1754 |
| hsa-mir-18b | 1977 | 1951 | 1529 | 1407 | 1079 | 1740 |
| hsa-mir-190 | 30 | 26 | 20 | 31 | 31 | 30 |
| hsa-mir-1908 | 0 | 1 | 1 | 0 | 0 | 0 |
| hsa-mir-1909 | 0 | 0 | 0 | 0 | 0 | 0 |
| hsa-mir-190b | 29 | 26 | 19 | 31 | 30 | 30 |
| hsa-mir-191 | 1327 | 1336 | 1094 | 2316 | 1980 | 2073 |
| hsa-mir-1910 | 6 | 5 | 3 | 2 | 3 | 2 |
| hsa-mir-1911 | 0 | 0 | 0 | 0 | 0 | 0 |
| hsa-mir-1912 | 0 | 0 | 0 | 0 | 0 | 0 |
| hsa-mir-1913 | 0 | 0 | 0 | 0 | 0 | 0 |
| hsa-mir-1914 | 0 | 0 | 0 | 0 | 0 | 0 |
| hsa-mir-1915 | 0 | 0 | 0 | 0 | 0 | 0 |
| hsa-mir-192 | 118 | 102 | 106 | 288 | 245 | 364 |
| hsa-mir-193a | 15 | 31 | 27 | 8 | 12 | 8 |
| hsa-mir-193b | 673 | 1048 | 1059 | 766 | 633 | 786 |
| hsa-mir-194-1 | 59 | 45 | 48 | 43 | 34 | 61 |
| hsa-mir-194-2 | 60 | 46 | 48 | 44 | 34 | 62 |
| hsa-mir-195 | 2 | 3 | 2 | 1 | 2 | 1 |
| hsa-mir-196a-1 | 113 | 124 | 123 | 137 | 132 | 149 |
| hsa-mir-196a-2 | 138 | 154 | 150 | 164 | 156 | 178 |
| hsa-mir-196b | 64 | 65 | 57 | 88 | 88 | 96 |
| hsa-mir-197 | 119 | 177 | 172 | 164 | 143 | 90 |
| hsa-mir-1972-1 | 1 | 2 | 1 | 1 | 1 | 1 |
| hsa-mir-1972-2 | 2 | 2 | 1 | 1 | 1 | 1 |
| hsa-mir-1973 | 5 | 15 | 1 | 5 | 7 | 2 |
| hsa-mir-1975 | 302 | 473 | 314 | 347 | 259 | 367 |
| hsa-mir-1976 | 1 | 2 | 2 | 2 | 1 | 2 |
| hsa-mir-1979 | 10 | 21 | 8 | 34 | 41 | 10 |
| hsa-mir-198 | 0 | 0 | 0 | 0 | 0 | 0 |
| hsa-mir-199a-1 | 1 | 2 | 1 | 1 | 1 | 1 |
| hsa-mir-199a-2 | 1 | 2 | 1 | 1 | 1 | 1 |
| hsa-mir-199b | 1 | 1 | 2 | 2 | 1 | 3 |
| hsa-mir-19a | 885 | 975 | 727 | 287 | 341 | 282 |
| hsa-mir-19b-1 | 2270 | 3016 | 2079 | 1599 | 1836 | 1105 |

| | | | | | | |
|---|---|---|---|---|---|---|
| hsa-mir-19b-2 | 2241 | 2986 | 2073 | 1589 | 1829 | 1066 |
| hsa-mir-200a | 24447 | 23238 | 21066 | 8036 | 8103 | 11810 |
| hsa-mir-200b | 142 | 129 | 143 | 223 | 190 | 244 |
| hsa-mir-200c | 6619 | 5811 | 5712 | 9377 | 9201 | 12495 |
| hsa-mir-202 | 0 | 1 | 0 | 0 | 0 | 0 |
| hsa-mir-203 | 191 | 160 | 350 | 961 | 528 | 3645 |
| hsa-mir-204 | 0 | 0 | 0 | 0 | 0 | 0 |
| hsa-mir-205 | 4914 | 7561 | 8260 | 6065 | 6156 | 7833 |
| hsa-mir-2052 | 0 | 0 | 0 | 0 | 0 | 0 |
| hsa-mir-2053 | 0 | 0 | 0 | 0 | 0 | 0 |
| hsa-mir-2054 | 9 | 7 | 8 | 5 | 4 | 14 |
| hsa-mir-206 | 0 | 0 | 0 | 0 | 0 | 0 |
| hsa-mir-208a | 0 | 0 | 0 | 0 | 0 | 0 |
| hsa-mir-208b | 0 | 0 | 0 | 0 | 0 | 0 |
| hsa-mir-20a | 8701 | 7874 | 7190 | 7470 | 6064 | 9208 |
| hsa-mir-20b | 0 | 0 | 0 | 0 | 0 | 0 |
| hsa-mir-21 | 108466 | 104005 | 129377 | 145234 | 102139 | 234270 |
| hsa-mir-210 | 602 | 897 | 748 | 647 | 669 | 917 |
| hsa-mir-211 | 0 | 0 | 0 | 0 | 0 | 0 |
| hsa-mir-2110 | 4 | 6 | 5 | 13 | 14 | 13 |
| hsa-mir-2113 | 0 | 0 | 0 | 0 | 0 | 0 |
| hsa-mir-2114 | 0 | 0 | 0 | 0 | 0 | 0 |
| hsa-mir-2115 | 0 | 0 | 0 | 0 | 0 | 0 |
| hsa-mir-2116 | 3 | 3 | 3 | 3 | 3 | 4 |
| hsa-mir-2117 | 0 | 0 | 0 | 0 | 1 | 0 |
| hsa-mir-212 | 2 | 1 | 1 | 1 | 1 | 1 |
| hsa-mir-214 | 0 | 1 | 1 | 0 | 1 | 0 |
| hsa-mir-215 | 50 | 41 | 27 | 83 | 82 | 78 |
| hsa-mir-216a | 0 | 0 | 0 | 0 | 0 | 0 |
| hsa-mir-216b | 0 | 0 | 0 | 0 | 0 | 0 |
| hsa-mir-217 | 0 | 0 | 0 | 0 | 0 | 1 |
| hsa-mir-218-1 | 14 | 19 | 15 | 55 | 66 | 41 |
| hsa-mir-218-2 | 15 | 20 | 15 | 54 | 65 | 41 |
| hsa-mir-219-1 | 5 | 4 | 5 | 5 | 3 | 5 |
| hsa-mir-219-2 | 4 | 4 | 4 | 3 | 3 | 3 |
| hsa-mir-22 | 779 | 950 | 1021 | 672 | 694 | 1194 |
| hsa-mir-220a | 0 | 0 | 0 | 0 | 0 | 0 |
| hsa-mir-220b | 0 | 0 | 0 | 0 | 0 | 0 |
| hsa-mir-220c | 0 | 0 | 0 | 0 | 0 | 0 |
| hsa-mir-221 | 2412 | 3020 | 2265 | 4663 | 4237 | 4295 |
| hsa-mir-222 | 2748 | 3156 | 2123 | 1999 | 1470 | 2122 |
| hsa-mir-223 | 0 | 0 | 0 | 0 | 0 | 0 |
| hsa-mir-224 | 120 | 140 | 85 | 121 | 100 | 84 |
| hsa-mir-2276 | 2 | 2 | 2 | 1 | 1 | 1 |
| hsa-mir-2277 | 6 | 7 | 9 | 2 | 2 | 3 |
| hsa-mir-2278 | 1 | 1 | 1 | 0 | 1 | 1 |
| hsa-mir-2355 | 25 | 25 | 14 | 29 | 23 | 30 |
| hsa-mir-23a | 13117 | 11145 | 10395 | 17573 | 13841 | 11918 |
| hsa-mir-23b | 13088 | 11122 | 10383 | 17542 | 13811 | 11879 |
| hsa-mir-24-1 | 72404 | 63966 | 75998 | 27885 | 29248 | 33051 |
| hsa-mir-24-2 | 72400 | 63966 | 75996 | 27885 | 29251 | 33051 |
| hsa-mir-25 | 384 | 437 | 389 | 476 | 462 | 436 |
| hsa-mir-26a-1 | 1839 | 1811 | 1933 | 1345 | 1789 | 1235 |

| | | | | | | |
|---|---|---|---|---|---|---|
| hsa-mir-26a-2 | 1848 | 1820 | 1945 | 1357 | 1814 | 1254 |
| hsa-mir-26b | 1150 | 1157 | 963 | 692 | 894 | 626 |
| hsa-mir-27a | 26063 | 27539 | 28150 | 25368 | 23722 | 30321 |
| hsa-mir-27b | 25691 | 27069 | 27856 | 24868 | 23300 | 29664 |
| hsa-mir-28 | 444 | 492 | 442 | 547 | 526 | 464 |
| hsa-mir-2861 | 0 | 0 | 0 | 0 | 0 | 0 |
| hsa-mir-2909 | 0 | 0 | 0 | 0 | 0 | 0 |
| hsa-mir-296 | 3 | 3 | 2 | 3 | 3 | 3 |
| hsa-mir-297 | 0 | 0 | 0 | 0 | 0 | 0 |
| hsa-mir-298 | 0 | 0 | 0 | 0 | 0 | 0 |
| hsa-mir-299 | 1 | 1 | 1 | 1 | 1 | 1 |
| hsa-mir-29a | 2718 | 3289 | 2796 | 2248 | 1872 | 3267 |
| hsa-mir-29b-1 | 9417 | 11816 | 10889 | 2824 | 3297 | 2929 |
| hsa-mir-29b-2 | 9391 | 11791 | 10877 | 2807 | 3281 | 2850 |
| hsa-mir-29c | 9387 | 11792 | 10858 | 2809 | 3283 | 2819 |
| hsa-mir-300 | 0 | 0 | 0 | 0 | 0 | 0 |
| hsa-mir-301a | 167 | 232 | 151 | 96 | 165 | 58 |
| hsa-mir-301b | 19 | 33 | 20 | 10 | 22 | 7 |
| hsa-mir-302a | 0 | 0 | 0 | 0 | 0 | 0 |
| hsa-mir-302b | 0 | 0 | 0 | 0 | 0 | 0 |
| hsa-mir-302c | 0 | 0 | 0 | 0 | 0 | 0 |
| hsa-mir-302d | 0 | 0 | 0 | 0 | 0 | 0 |
| hsa-mir-302e | 0 | 0 | 0 | 0 | 0 | 0 |
| hsa-mir-302f | 0 | 0 | 0 | 0 | 0 | 0 |
| hsa-mir-3065 | 39 | 37 | 37 | 71 | 53 | 102 |
| hsa-mir-3074 | 72404 | 63966 | 75998 | 27885 | 29248 | 33051 |
| hsa-mir-30a | 7206 | 6762 | 7761 | 23491 | 16662 | 21598 |
| hsa-mir-30b | 3965 | 4494 | 4163 | 3730 | 3663 | 2932 |
| hsa-mir-30c-1 | 3958 | 4489 | 4161 | 3729 | 3661 | 2930 |
| hsa-mir-30c-2 | 3972 | 4500 | 4171 | 3760 | 3680 | 2954 |
| hsa-mir-30d | 3071 | 3002 | 3110 | 8232 | 6210 | 7937 |
| hsa-mir-30e | 7059 | 6337 | 8005 | 10059 | 8490 | 13333 |
| hsa-mir-31 | 62522 | 77945 | 73235 | 8066 | 9364 | 4568 |
| hsa-mir-3115 | 0 | 0 | 0 | 0 | 0 | 0 |
| hsa-mir-3116-1 | 0 | 0 | 1 | 0 | 0 | 0 |
| hsa-mir-3116-2 | 0 | 0 | 1 | 0 | 0 | 0 |
| hsa-mir-3117 | 0 | 0 | 0 | 0 | 0 | 0 |
| hsa-mir-3118-1 | 0 | 0 | 0 | 0 | 0 | 0 |
| hsa-mir-3118-2 | 0 | 0 | 0 | 0 | 0 | 0 |
| hsa-mir-3118-3 | 0 | 0 | 0 | 0 | 0 | 0 |
| hsa-mir-3118-4 | 0 | 0 | 0 | 0 | 0 | 0 |
| hsa-mir-3118-5 | 0 | 0 | 0 | 0 | 0 | 0 |
| hsa-mir-3118-6 | 0 | 0 | 0 | 0 | 0 | 0 |
| hsa-mir-3119-1 | 0 | 0 | 0 | 0 | 0 | 0 |
| hsa-mir-3119-2 | 0 | 0 | 0 | 0 | 0 | 0 |
| hsa-mir-3120 | 0 | 1 | 1 | 0 | 1 | 0 |
| hsa-mir-3121 | 1 | 0 | 0 | 1 | 2 | 0 |
| hsa-mir-3122 | 0 | 0 | 0 | 0 | 0 | 0 |
| hsa-mir-3123 | 37 | 82 | 13 | 48 | 111 | 25 |
| hsa-mir-3124 | 0 | 0 | 0 | 0 | 0 | 0 |
| hsa-mir-3125 | 0 | 0 | 0 | 0 | 1 | 0 |
| hsa-mir-3126 | 0 | 0 | 0 | 0 | 0 | 0 |
| hsa-mir-3127 | 0 | 0 | 0 | 0 | 0 | 0 |

| | | | | | | |
|---|---|---|---|---|---|---|
| hsa-mir-3128 | 1 | 0 | 0 | 2 | 1 | 1 |
| hsa-mir-3129 | 0 | 0 | 0 | 0 | 0 | 0 |
| hsa-mir-3130-1 | 2 | 1 | 3 | 3 | 2 | 2 |
| hsa-mir-3130-2 | 2 | 1 | 3 | 3 | 2 | 2 |
| hsa-mir-3130-3 | 2 | 1 | 3 | 3 | 2 | 2 |
| hsa-mir-3130-4 | 2 | 1 | 3 | 3 | 2 | 2 |
| hsa-mir-3131 | 0 | 0 | 0 | 0 | 0 | 0 |
| hsa-mir-3132 | 0 | 0 | 0 | 0 | 0 | 0 |
| hsa-mir-3133 | 0 | 0 | 0 | 1 | 0 | 1 |
| hsa-mir-3134 | 1 | 0 | 1 | 1 | 0 | 0 |
| hsa-mir-3135 | 0 | 0 | 0 | 1 | 1 | 0 |
| hsa-mir-3136 | 0 | 1 | 1 | 2 | 1 | 1 |
| hsa-mir-3137 | 0 | 0 | 0 | 0 | 0 | 0 |
| hsa-mir-3138 | 1 | 1 | 1 | 1 | 1 | 1 |
| hsa-mir-3139 | 0 | 0 | 0 | 0 | 0 | 0 |
| hsa-mir-3140 | 0 | 0 | 0 | 0 | 0 | 0 |
| hsa-mir-3141 | 0 | 0 | 0 | 0 | 0 | 0 |
| hsa-mir-3142 | 0 | 0 | 0 | 1 | 0 | 0 |
| hsa-mir-3143 | 0 | 0 | 0 | 0 | 0 | 0 |
| hsa-mir-3144 | 0 | 0 | 0 | 0 | 0 | 0 |
| hsa-mir-3145 | 0 | 0 | 1 | 1 | 1 | 1 |
| hsa-mir-3146 | 0 | 0 | 0 | 0 | 1 | 0 |
| hsa-mir-3147 | 0 | 0 | 0 | 0 | 0 | 0 |
| hsa-mir-3148 | 0 | 0 | 0 | 0 | 0 | 0 |
| hsa-mir-3149 | 0 | 0 | 0 | 0 | 0 | 0 |
| hsa-mir-3150 | 0 | 1 | 0 | 0 | 1 | 0 |
| hsa-mir-3151 | 0 | 0 | 0 | 0 | 0 | 0 |
| hsa-mir-3152 | 0 | 0 | 0 | 0 | 0 | 0 |
| hsa-mir-3153 | 0 | 0 | 0 | 0 | 0 | 0 |
| hsa-mir-3154 | 0 | 0 | 0 | 0 | 0 | 0 |
| hsa-mir-3155 | 0 | 0 | 0 | 0 | 0 | 0 |
| hsa-mir-3156-1 | 0 | 0 | 0 | 0 | 0 | 0 |
| hsa-mir-3156-2 | 0 | 0 | 0 | 0 | 0 | 0 |
| hsa-mir-3156-3 | 0 | 0 | 0 | 0 | 0 | 0 |
| hsa-mir-3157 | 0 | 1 | 0 | 1 | 1 | 0 |
| hsa-mir-3158-1 | 22 | 19 | 19 | 33 | 33 | 44 |
| hsa-mir-3158-2 | 22 | 19 | 19 | 33 | 33 | 44 |
| hsa-mir-3159 | 0 | 0 | 0 | 0 | 0 | 0 |
| hsa-mir-3160-1 | 0 | 0 | 0 | 0 | 0 | 0 |
| hsa-mir-3160-2 | 0 | 0 | 0 | 0 | 0 | 0 |
| hsa-mir-3161 | 0 | 0 | 0 | 0 | 0 | 0 |
| hsa-mir-3162 | 0 | 0 | 0 | 0 | 0 | 0 |
| hsa-mir-3163 | 0 | 1 | 0 | 1 | 1 | 1 |
| hsa-mir-3164 | 1 | 1 | 2 | 1 | 1 | 1 |
| hsa-mir-3165 | 0 | 0 | 0 | 0 | 0 | 0 |
| hsa-mir-3166 | 1 | 1 | 1 | 2 | 1 | 1 |
| hsa-mir-3167 | 0 | 0 | 1 | 0 | 0 | 0 |
| hsa-mir-3168 | 0 | 0 | 0 | 0 | 0 | 0 |
| hsa-mir-3169 | 0 | 0 | 0 | 0 | 0 | 0 |
| hsa-mir-3170 | 0 | 0 | 0 | 0 | 0 | 0 |
| hsa-mir-3171 | 0 | 0 | 0 | 0 | 0 | 0 |
| hsa-mir-3172 | 14 | 28 | 7 | 38 | 29 | 29 |
| hsa-mir-3173 | 1 | 1 | 0 | 1 | 1 | 1 |

| | | | | | | |
|---|---|---|---|---|---|---|
| hsa-mir-3174 | 1 | 1 | 1 | 0 | 1 | 0 |
| hsa-mir-3175 | 0 | 0 | 0 | 0 | 0 | 0 |
| hsa-mir-3176 | 6 | 6 | 6 | 7 | 5 | 10 |
| hsa-mir-3177 | 1 | 0 | 1 | 1 | 2 | 1 |
| hsa-mir-3178 | 19 | 25 | 20 | 4 | 3 | 78 |
| hsa-mir-3179-1 | 1 | 0 | 0 | 1 | 0 | 0 |
| hsa-mir-3179-2 | 1 | 0 | 0 | 1 | 0 | 0 |
| hsa-mir-3179-3 | 1 | 0 | 0 | 1 | 0 | 0 |
| hsa-mir-3180-1 | 0 | 0 | 0 | 0 | 0 | 0 |
| hsa-mir-3180-2 | 0 | 0 | 0 | 0 | 0 | 0 |
| hsa-mir-3180-3 | 0 | 0 | 0 | 0 | 0 | 0 |
| hsa-mir-3181 | 0 | 0 | 0 | 0 | 0 | 0 |
| hsa-mir-3182 | 27 | 29 | 26 | 50 | 44 | 56 |
| hsa-mir-3183 | 0 | 0 | 0 | 0 | 0 | 0 |
| hsa-mir-3184 | 829 | 855 | 735 | 1008 | 1263 | 891 |
| hsa-mir-3185 | 0 | 0 | 0 | 0 | 0 | 0 |
| hsa-mir-3186 | 0 | 0 | 0 | 0 | 0 | 0 |
| hsa-mir-3187 | 1 | 3 | 1 | 2 | 1 | 2 |
| hsa-mir-3188 | 0 | 1 | 0 | 0 | 1 | 0 |
| hsa-mir-3189 | 0 | 0 | 0 | 0 | 0 | 0 |
| hsa-mir-3190 | 0 | 0 | 0 | 0 | 0 | 0 |
| hsa-mir-3191 | 0 | 0 | 0 | 0 | 0 | 0 |
| hsa-mir-3192 | 0 | 0 | 0 | 0 | 0 | 0 |
| hsa-mir-3193 | 0 | 0 | 0 | 0 | 0 | 0 |
| hsa-mir-3194 | 0 | 0 | 0 | 0 | 0 | 0 |
| hsa-mir-3195 | 17 | 43 | 16 | 34 | 24 | 22 |
| hsa-mir-3196 | 2 | 3 | 1 | 1 | 1 | 2 |
| hsa-mir-3197 | 0 | 0 | 0 | 0 | 0 | 0 |
| hsa-mir-3198 | 0 | 0 | 0 | 0 | 0 | 0 |
| hsa-mir-3199-1 | 2 | 2 | 2 | 1 | 1 | 2 |
| hsa-mir-3199-2 | 2 | 2 | 2 | 1 | 1 | 2 |
| hsa-mir-32 | 560 | 933 | 582 | 405 | 703 | 261 |
| hsa-mir-3200 | 7 | 9 | 6 | 13 | 7 | 7 |
| hsa-mir-3201 | 0 | 0 | 0 | 0 | 0 | 0 |
| hsa-mir-3202-1 | 0 | 0 | 0 | 0 | 0 | 0 |
| hsa-mir-3202-2 | 0 | 0 | 0 | 0 | 0 | 0 |
| hsa-mir-320a | 596 | 582 | 445 | 461 | 589 | 354 |
| hsa-mir-320b-1 | 594 | 580 | 443 | 459 | 587 | 347 |
| hsa-mir-320b-2 | 594 | 580 | 443 | 458 | 587 | 347 |
| hsa-mir-320c-1 | 594 | 580 | 442 | 458 | 587 | 346 |
| hsa-mir-320c-2 | 594 | 580 | 442 | 458 | 587 | 346 |
| hsa-mir-320d-1 | 587 | 577 | 438 | 457 | 586 | 340 |
| hsa-mir-320d-2 | 587 | 577 | 438 | 457 | 586 | 340 |
| hsa-mir-320e | 575 | 565 | 430 | 451 | 573 | 334 |
| hsa-mir-323 | 0 | 1 | 0 | 0 | 0 | 1 |
| hsa-mir-323b | 0 | 0 | 0 | 0 | 0 | 0 |
| hsa-mir-324 | 59 | 72 | 67 | 59 | 47 | 53 |
| hsa-mir-325 | 0 | 0 | 0 | 0 | 0 | 0 |
| hsa-mir-326 | 0 | 0 | 0 | 0 | 0 | 0 |
| hsa-mir-328 | 3 | 5 | 6 | 4 | 3 | 4 |
| hsa-mir-329-1 | 1 | 0 | 0 | 1 | 1 | 0 |
| hsa-mir-329-2 | 1 | 0 | 0 | 1 | 1 | 0 |
| hsa-mir-330 | 196 | 179 | 167 | 60 | 55 | 51 |

| | | | | | | |
|---|---|---|---|---|---|---|
| hsa-mir-331 | 148 | 156 | 151 | 155 | 133 | 182 |
| hsa-mir-335 | 86 | 87 | 68 | 144 | 148 | 162 |
| hsa-mir-337 | 0 | 0 | 0 | 0 | 0 | 0 |
| hsa-mir-338 | 39 | 37 | 37 | 71 | 53 | 102 |
| hsa-mir-339 | 202 | 243 | 261 | 172 | 145 | 179 |
| hsa-mir-33a | 795 | 942 | 898 | 170 | 177 | 269 |
| hsa-mir-33b | 269 | 398 | 453 | 50 | 54 | 99 |
| hsa-mir-340 | 168 | 135 | 89 | 102 | 153 | 77 |
| hsa-mir-342 | 169 | 258 | 239 | 164 | 140 | 138 |
| hsa-mir-345 | 61 | 49 | 41 | 30 | 18 | 23 |
| hsa-mir-346 | 0 | 0 | 0 | 0 | 0 | 0 |
| hsa-mir-34a | 565 | 579 | 719 | 645 | 694 | 821 |
| hsa-mir-34b | 67 | 72 | 59 | 46 | 44 | 45 |
| hsa-mir-34c | 3631 | 4145 | 3437 | 4564 | 5094 | 2699 |
| hsa-mir-361 | 162 | 128 | 138 | 146 | 127 | 184 |
| hsa-mir-362 | 20 | 25 | 20 | 17 | 18 | 18 |
| hsa-mir-363 | 0 | 0 | 0 | 0 | 0 | 0 |
| hsa-mir-365-1 | 179 | 178 | 175 | 215 | 214 | 308 |
| hsa-mir-365-2 | 176 | 173 | 173 | 209 | 207 | 302 |
| hsa-mir-367 | 0 | 0 | 0 | 0 | 0 | 0 |
| hsa-mir-369 | 2 | 1 | 1 | 1 | 2 | 4 |
| hsa-mir-370 | 0 | 0 | 0 | 0 | 0 | 0 |
| hsa-mir-371 | 0 | 0 | 0 | 0 | 0 | 0 |
| hsa-mir-372 | 0 | 0 | 0 | 0 | 0 | 0 |
| hsa-mir-373 | 0 | 0 | 0 | 1 | 0 | 0 |
| hsa-mir-374a | 1878 | 1733 | 1021 | 4066 | 4178 | 2416 |
| hsa-mir-374b | 326 | 275 | 209 | 207 | 204 | 146 |
| hsa-mir-375 | 2 | 2 | 3 | 4 | 5 | 4 |
| hsa-mir-376a-1 | 13 | 11 | 12 | 9 | 6 | 17 |
| hsa-mir-376a-2 | 6 | 6 | 7 | 4 | 4 | 4 |
| hsa-mir-376b | 4 | 4 | 4 | 3 | 2 | 3 |
| hsa-mir-376c | 9 | 8 | 9 | 4 | 4 | 3 |
| hsa-mir-377 | 2 | 2 | 1 | 1 | 0 | 1 |
| hsa-mir-378 | 35123 | 38004 | 25346 | 64406 | 80185 | 25212 |
| hsa-mir-378b | 4 | 1 | 0 | 3 | 1 | 1 |
| hsa-mir-378c | 35117 | 37996 | 25337 | 64398 | 80177 | 25205 |
| hsa-mir-379 | 7 | 9 | 9 | 17 | 20 | 18 |
| hsa-mir-380 | 0 | 0 | 1 | 1 | 1 | 1 |
| hsa-mir-381 | 11 | 10 | 15 | 15 | 14 | 18 |
| hsa-mir-382 | 1 | 0 | 1 | 1 | 1 | 1 |
| hsa-mir-383 | 0 | 0 | 0 | 0 | 0 | 0 |
| hsa-mir-384 | 0 | 0 | 0 | 0 | 0 | 0 |
| hsa-mir-409 | 1 | 1 | 1 | 1 | 1 | 1 |
| hsa-mir-410 | 1 | 0 | 1 | 1 | 1 | 1 |
| hsa-mir-411 | 6 | 8 | 7 | 3 | 3 | 2 |
| hsa-mir-412 | 0 | 0 | 0 | 0 | 0 | 0 |
| hsa-mir-421 | 23 | 24 | 15 | 17 | 20 | 12 |
| hsa-mir-422a | 0 | 0 | 0 | 0 | 0 | 0 |
| hsa-mir-423 | 829 | 855 | 735 | 1008 | 1263 | 891 |
| hsa-mir-424 | 845 | 1107 | 1237 | 441 | 820 | 518 |
| hsa-mir-425 | 2030 | 2460 | 2033 | 1405 | 1234 | 761 |
| hsa-mir-4251 | 0 | 0 | 0 | 0 | 0 | 0 |
| hsa-mir-4252 | 0 | 0 | 0 | 0 | 0 | 0 |

| | | | | | | |
|---|---|---|---|---|---|---|
| hsa-mir-4253 | 0 | 0 | 0 | 0 | 0 | 0 |
| hsa-mir-4254 | 0 | 0 | 0 | 0 | 0 | 0 |
| hsa-mir-4255 | 0 | 0 | 0 | 0 | 0 | 0 |
| hsa-mir-4256 | 0 | 0 | 0 | 0 | 0 | 0 |
| hsa-mir-4257 | 0 | 0 | 0 | 0 | 0 | 0 |
| hsa-mir-4258 | 0 | 0 | 0 | 0 | 0 | 0 |
| hsa-mir-4259 | 0 | 0 | 0 | 0 | 0 | 0 |
| hsa-mir-4260 | 0 | 0 | 0 | 0 | 0 | 0 |
| hsa-mir-4261 | 0 | 0 | 0 | 0 | 0 | 0 |
| hsa-mir-4262 | 0 | 0 | 0 | 0 | 0 | 0 |
| hsa-mir-4263 | 0 | 0 | 0 | 0 | 0 | 0 |
| hsa-mir-4264 | 0 | 0 | 0 | 0 | 0 | 0 |
| hsa-mir-4265 | 0 | 0 | 0 | 0 | 0 | 0 |
| hsa-mir-4266 | 0 | 0 | 0 | 0 | 0 | 0 |
| hsa-mir-4267 | 0 | 0 | 0 | 0 | 0 | 0 |
| hsa-mir-4268 | 0 | 0 | 0 | 0 | 0 | 0 |
| hsa-mir-4269 | 0 | 0 | 0 | 0 | 0 | 0 |
| hsa-mir-4270 | 0 | 0 | 0 | 0 | 0 | 0 |
| hsa-mir-4271 | 0 | 0 | 0 | 0 | 0 | 0 |
| hsa-mir-4272 | 0 | 0 | 0 | 0 | 0 | 0 |
| hsa-mir-4273 | 0 | 0 | 0 | 0 | 0 | 0 |
| hsa-mir-4274 | 0 | 0 | 0 | 0 | 0 | 0 |
| hsa-mir-4275 | 0 | 0 | 0 | 0 | 0 | 0 |
| hsa-mir-4276 | 0 | 0 | 0 | 0 | 0 | 0 |
| hsa-mir-4277 | 0 | 0 | 0 | 0 | 0 | 0 |
| hsa-mir-4278 | 0 | 0 | 0 | 0 | 0 | 0 |
| hsa-mir-4279 | 0 | 0 | 0 | 0 | 0 | 0 |
| hsa-mir-4280 | 0 | 0 | 0 | 0 | 0 | 0 |
| hsa-mir-4281 | 0 | 0 | 0 | 0 | 0 | 0 |
| hsa-mir-4282 | 0 | 0 | 0 | 0 | 0 | 0 |
| hsa-mir-4283-1 | 0 | 0 | 0 | 0 | 0 | 0 |
| hsa-mir-4283-2 | 0 | 0 | 0 | 0 | 0 | 0 |
| hsa-mir-4284 | 21 | 37 | 9 | 27 | 22 | 13 |
| hsa-mir-4285 | 0 | 0 | 0 | 0 | 0 | 0 |
| hsa-mir-4286 | 1 | 1 | 1 | 1 | 1 | 1 |
| hsa-mir-4287 | 0 | 0 | 0 | 0 | 0 | 0 |
| hsa-mir-4288 | 0 | 0 | 0 | 0 | 0 | 0 |
| hsa-mir-4289 | 0 | 0 | 0 | 0 | 0 | 0 |
| hsa-mir-429 | 38 | 34 | 35 | 67 | 50 | 98 |
| hsa-mir-4290 | 0 | 0 | 0 | 0 | 0 | 0 |
| hsa-mir-4291 | 0 | 0 | 0 | 0 | 0 | 0 |
| hsa-mir-4292 | 0 | 0 | 0 | 0 | 0 | 0 |
| hsa-mir-4293 | 0 | 0 | 0 | 0 | 0 | 0 |
| hsa-mir-4294 | 0 | 0 | 0 | 0 | 0 | 0 |
| hsa-mir-4295 | 0 | 0 | 0 | 0 | 0 | 0 |
| hsa-mir-4296 | 0 | 0 | 0 | 0 | 0 | 0 |
| hsa-mir-4297 | 0 | 0 | 0 | 0 | 0 | 0 |
| hsa-mir-4298 | 0 | 0 | 0 | 0 | 0 | 0 |
| hsa-mir-4299 | 0 | 0 | 0 | 0 | 0 | 0 |
| hsa-mir-4300 | 0 | 0 | 0 | 0 | 0 | 0 |
| hsa-mir-4301 | 0 | 0 | 0 | 0 | 0 | 0 |
| hsa-mir-4302 | 0 | 0 | 0 | 0 | 0 | 0 |
| hsa-mir-4303 | 0 | 0 | 0 | 0 | 0 | 0 |

| | | | | | | |
|---|---|---|---|---|---|---|
| hsa-mir-4304 | 0 | 0 | 0 | 0 | 0 | 0 |
| hsa-mir-4305 | 0 | 0 | 0 | 0 | 0 | 0 |
| hsa-mir-4306 | 153 | 117 | 83 | 88 | 104 | 85 |
| hsa-mir-4307 | 0 | 0 | 0 | 0 | 0 | 0 |
| hsa-mir-4308 | 0 | 0 | 0 | 0 | 0 | 0 |
| hsa-mir-4309 | 0 | 0 | 0 | 0 | 0 | 0 |
| hsa-mir-431 | 0 | 0 | 0 | 0 | 0 | 0 |
| hsa-mir-4310 | 0 | 0 | 0 | 0 | 0 | 0 |
| hsa-mir-4311 | 0 | 0 | 0 | 0 | 0 | 0 |
| hsa-mir-4312 | 0 | 0 | 0 | 0 | 0 | 0 |
| hsa-mir-4313 | 0 | 0 | 0 | 0 | 0 | 0 |
| hsa-mir-4314 | 0 | 0 | 0 | 0 | 0 | 0 |
| hsa-mir-4315-1 | 0 | 0 | 0 | 0 | 0 | 0 |
| hsa-mir-4315-2 | 0 | 0 | 0 | 0 | 0 | 0 |
| hsa-mir-4316 | 0 | 0 | 0 | 0 | 0 | 0 |
| hsa-mir-4317 | 0 | 0 | 0 | 0 | 0 | 0 |
| hsa-mir-4318 | 0 | 0 | 0 | 0 | 0 | 0 |
| hsa-mir-4319 | 0 | 0 | 0 | 0 | 0 | 0 |
| hsa-mir-432 | 0 | 0 | 0 | 0 | 0 | 0 |
| hsa-mir-4320 | 0 | 0 | 0 | 0 | 0 | 0 |
| hsa-mir-4321 | 0 | 0 | 0 | 0 | 0 | 0 |
| hsa-mir-4322 | 0 | 0 | 0 | 0 | 0 | 0 |
| hsa-mir-4323 | 0 | 0 | 0 | 0 | 0 | 0 |
| hsa-mir-4324 | 1 | 1 | 0 | 1 | 1 | 1 |
| hsa-mir-4325 | 0 | 0 | 0 | 0 | 0 | 0 |
| hsa-mir-4326 | 2 | 3 | 2 | 5 | 4 | 3 |
| hsa-mir-4327 | 0 | 0 | 0 | 0 | 0 | 0 |
| hsa-mir-4328 | 0 | 0 | 0 | 0 | 0 | 0 |
| hsa-mir-4329 | 0 | 0 | 0 | 0 | 0 | 0 |
| hsa-mir-433 | 0 | 0 | 0 | 0 | 0 | 0 |
| hsa-mir-4330 | 0 | 0 | 0 | 0 | 0 | 0 |
| hsa-mir-448 | 0 | 0 | 0 | 0 | 0 | 0 |
| hsa-mir-449a | 1 | 2 | 1 | 1 | 2 | 1 |
| hsa-mir-449b | 1 | 1 | 1 | 0 | 0 | 0 |
| hsa-mir-449c | 1 | 1 | 1 | 2 | 1 | 1 |
| hsa-mir-450a-1 | 12 | 18 | 17 | 20 | 19 | 25 |
| hsa-mir-450a-2 | 12 | 18 | 17 | 20 | 19 | 25 |
| hsa-mir-450b | 8 | 9 | 10 | 14 | 11 | 34 |
| hsa-mir-451 | 1 | 0 | 0 | 0 | 0 | 0 |
| hsa-mir-452 | 365 | 309 | 205 | 407 | 498 | 430 |
| hsa-mir-454 | 108 | 134 | 98 | 140 | 158 | 112 |
| hsa-mir-455 | 250 | 310 | 203 | 301 | 268 | 167 |
| hsa-mir-466 | 0 | 0 | 0 | 0 | 0 | 0 |
| hsa-mir-483 | 0 | 0 | 0 | 0 | 0 | 0 |
| hsa-mir-484 | 37 | 50 | 41 | 43 | 39 | 32 |
| hsa-mir-485 | 0 | 1 | 1 | 1 | 1 | 1 |
| hsa-mir-486 | 1 | 1 | 0 | 2 | 2 | 2 |
| hsa-mir-487a | 0 | 1 | 1 | 0 | 0 | 0 |
| hsa-mir-487b | 0 | 1 | 0 | 0 | 0 | 0 |
| hsa-mir-488 | 0 | 0 | 0 | 0 | 0 | 0 |
| hsa-mir-489 | 1 | 1 | 0 | 0 | 0 | 0 |
| hsa-mir-490 | 0 | 0 | 0 | 0 | 0 | 0 |
| hsa-mir-491 | 4 | 4 | 5 | 5 | 3 | 4 |

| | | | | | | |
|---|---|---|---|---|---|---|
| hsa-mir-492 | 0 | 0 | 0 | 0 | 0 | 0 |
| hsa-mir-493 | 0 | 0 | 0 | 0 | 0 | 1 |
| hsa-mir-494 | 5 | 6 | 6 | 3 | 3 | 5 |
| hsa-mir-495 | 2 | 2 | 2 | 2 | 2 | 2 |
| hsa-mir-496 | 0 | 0 | 0 | 1 | 0 | 0 |
| hsa-mir-497 | 2 | 1 | 2 | 2 | 2 | 3 |
| hsa-mir-498 | 0 | 0 | 0 | 0 | 0 | 0 |
| hsa-mir-499 | 2 | 3 | 2 | 1 | 2 | 1 |
| hsa-mir-500 | 30 | 45 | 27 | 41 | 35 | 37 |
| hsa-mir-500b | 7 | 9 | 7 | 11 | 7 | 8 |
| hsa-mir-501 | 5 | 6 | 4 | 7 | 7 | 9 |
| hsa-mir-502 | 25 | 41 | 23 | 35 | 32 | 32 |
| hsa-mir-503 | 257 | 293 | 372 | 224 | 255 | 304 |
| hsa-mir-504 | 0 | 0 | 0 | 0 | 0 | 0 |
| hsa-mir-505 | 37 | 46 | 34 | 45 | 33 | 24 |
| hsa-mir-506 | 0 | 0 | 0 | 0 | 0 | 0 |
| hsa-mir-507 | 0 | 0 | 0 | 0 | 0 | 0 |
| hsa-mir-508 | 0 | 0 | 0 | 0 | 0 | 0 |
| hsa-mir-509-1 | 0 | 0 | 0 | 0 | 0 | 0 |
| hsa-mir-509-2 | 0 | 0 | 0 | 0 | 0 | 0 |
| hsa-mir-509-3 | 0 | 0 | 0 | 0 | 0 | 0 |
| hsa-mir-510 | 0 | 0 | 0 | 0 | 0 | 0 |
| hsa-mir-511-1 | 0 | 1 | 0 | 0 | 1 | 0 |
| hsa-mir-511-2 | 0 | 1 | 0 | 0 | 1 | 0 |
| hsa-mir-512-1 | 0 | 0 | 0 | 0 | 0 | 0 |
| hsa-mir-512-2 | 0 | 0 | 0 | 0 | 0 | 0 |
| hsa-mir-513a-1 | 0 | 0 | 0 | 0 | 0 | 0 |
| hsa-mir-513a-2 | 0 | 0 | 0 | 0 | 0 | 0 |
| hsa-mir-513b | 0 | 0 | 0 | 0 | 0 | 0 |
| hsa-mir-513c | 0 | 0 | 0 | 0 | 0 | 0 |
| hsa-mir-514-1 | 0 | 0 | 0 | 0 | 0 | 1 |
| hsa-mir-514-2 | 0 | 0 | 0 | 0 | 0 | 1 |
| hsa-mir-514-3 | 0 | 0 | 0 | 0 | 0 | 1 |
| hsa-mir-514b | 0 | 0 | 0 | 0 | 0 | 0 |
| hsa-mir-515-1 | 0 | 0 | 0 | 0 | 0 | 0 |
| hsa-mir-515-2 | 0 | 0 | 0 | 0 | 0 | 0 |
| hsa-mir-516a-1 | 0 | 0 | 0 | 0 | 0 | 0 |
| hsa-mir-516a-2 | 0 | 0 | 0 | 0 | 0 | 0 |
| hsa-mir-516b-1 | 0 | 0 | 0 | 0 | 0 | 0 |
| hsa-mir-516b-2 | 0 | 0 | 0 | 0 | 0 | 0 |
| hsa-mir-517a | 0 | 0 | 0 | 0 | 0 | 0 |
| hsa-mir-517b | 0 | 0 | 0 | 0 | 0 | 0 |
| hsa-mir-517c | 0 | 0 | 0 | 0 | 0 | 0 |
| hsa-mir-518a-1 | 0 | 0 | 0 | 0 | 0 | 0 |
| hsa-mir-518a-2 | 0 | 0 | 0 | 0 | 0 | 0 |
| hsa-mir-518b | 0 | 0 | 0 | 0 | 0 | 0 |
| hsa-mir-518c | 0 | 0 | 0 | 0 | 0 | 0 |
| hsa-mir-518d | 0 | 0 | 0 | 0 | 0 | 0 |
| hsa-mir-518e | 0 | 0 | 0 | 0 | 0 | 0 |
| hsa-mir-518f | 0 | 0 | 0 | 0 | 0 | 0 |
| hsa-mir-519a-1 | 0 | 0 | 0 | 0 | 0 | 0 |
| hsa-mir-519a-2 | 0 | 0 | 0 | 0 | 0 | 0 |
| hsa-mir-519b | 0 | 0 | 0 | 0 | 0 | 0 |

| | | | | | | |
|---|---|---|---|---|---|---|
| hsa-mir-519c | 0 | 0 | 0 | 0 | 1 | 0 |
| hsa-mir-519d | 0 | 0 | 0 | 0 | 0 | 0 |
| hsa-mir-519e | 0 | 0 | 0 | 0 | 0 | 0 |
| hsa-mir-520a | 0 | 0 | 0 | 0 | 0 | 0 |
| hsa-mir-520b | 0 | 0 | 0 | 0 | 0 | 0 |
| hsa-mir-520c | 0 | 0 | 0 | 0 | 0 | 0 |
| hsa-mir-520d | 0 | 0 | 0 | 0 | 0 | 0 |
| hsa-mir-520e | 0 | 0 | 0 | 0 | 0 | 0 |
| hsa-mir-520f | 0 | 0 | 0 | 0 | 0 | 0 |
| hsa-mir-520g | 0 | 0 | 0 | 0 | 0 | 0 |
| hsa-mir-520h | 0 | 0 | 0 | 0 | 0 | 0 |
| hsa-mir-521-1 | 0 | 0 | 0 | 0 | 0 | 0 |
| hsa-mir-521-2 | 0 | 0 | 0 | 0 | 0 | 0 |
| hsa-mir-522 | 0 | 0 | 0 | 0 | 0 | 0 |
| hsa-mir-523 | 0 | 0 | 0 | 0 | 0 | 0 |
| hsa-mir-524 | 0 | 0 | 0 | 0 | 0 | 0 |
| hsa-mir-525 | 0 | 0 | 0 | 0 | 0 | 0 |
| hsa-mir-526a-1 | 0 | 0 | 0 | 0 | 0 | 0 |
| hsa-mir-526a-2 | 0 | 0 | 0 | 0 | 0 | 0 |
| hsa-mir-526b | 0 | 0 | 0 | 0 | 0 | 0 |
| hsa-mir-527 | 0 | 0 | 0 | 0 | 0 | 0 |
| hsa-mir-532 | 77 | 90 | 95 | 104 | 86 | 125 |
| hsa-mir-539 | 1 | 1 | 1 | 0 | 1 | 1 |
| hsa-mir-541 | 0 | 0 | 0 | 0 | 0 | 0 |
| hsa-mir-542 | 170 | 139 | 145 | 210 | 214 | 313 |
| hsa-mir-543 | 0 | 0 | 1 | 0 | 0 | 0 |
| hsa-mir-544 | 0 | 0 | 0 | 0 | 0 | 0 |
| hsa-mir-544b | 0 | 0 | 0 | 0 | 0 | 0 |
| hsa-mir-545 | 12 | 10 | 7 | 4 | 4 | 3 |
| hsa-mir-548a-1 | 3 | 2 | 2 | 1 | 2 | 3 |
| hsa-mir-548a-2 | 1 | 1 | 1 | 1 | 1 | 1 |
| hsa-mir-548a-3 | 2 | 2 | 1 | 1 | 2 | 2 |
| hsa-mir-548b | 0 | 0 | 0 | 0 | 1 | 0 |
| hsa-mir-548c | 1 | 3 | 2 | 2 | 2 | 1 |
| hsa-mir-548d-1 | 5 | 5 | 4 | 4 | 5 | 5 |
| hsa-mir-548d-2 | 7 | 9 | 6 | 5 | 7 | 4 |
| hsa-mir-548e | 9 | 9 | 6 | 16 | 23 | 19 |
| hsa-mir-548f-1 | 44 | 37 | 23 | 72 | 66 | 65 |
| hsa-mir-548f-2 | 16 | 16 | 6 | 41 | 41 | 22 |
| hsa-mir-548f-3 | 15 | 15 | 6 | 41 | 41 | 21 |
| hsa-mir-548f-4 | 15 | 15 | 6 | 41 | 41 | 21 |
| hsa-mir-548f-5 | 2 | 1 | 1 | 10 | 10 | 5 |
| hsa-mir-548g | 2 | 1 | 1 | 11 | 11 | 5 |
| hsa-mir-548h-1 | 3 | 2 | 2 | 4 | 3 | 8 |
| hsa-mir-548h-2 | 3 | 2 | 2 | 4 | 3 | 8 |
| hsa-mir-548h-3 | 9 | 8 | 7 | 7 | 7 | 11 |
| hsa-mir-548h-4 | 3 | 2 | 2 | 4 | 3 | 8 |
| hsa-mir-548i-1 | 0 | 0 | 0 | 0 | 0 | 0 |
| hsa-mir-548i-2 | 0 | 0 | 0 | 0 | 0 | 0 |
| hsa-mir-548i-3 | 0 | 0 | 0 | 0 | 0 | 0 |
| hsa-mir-548i-4 | 0 | 0 | 0 | 0 | 0 | 0 |
| hsa-mir-548j | 3 | 2 | 2 | 4 | 4 | 7 |
| hsa-mir-548k | 3 | 3 | 3 | 6 | 4 | 6 |

| | | | | | | |
|---|---|---|---|---|---|---|
| hsa-mir-548l | 2 | 1 | 1 | 1 | 2 | 1 |
| hsa-mir-548m | 0 | 0 | 0 | 0 | 0 | 0 |
| hsa-mir-548n | 1 | 1 | 1 | 1 | 1 | 2 |
| hsa-mir-548o | 11 | 13 | 10 | 15 | 17 | 12 |
| hsa-mir-548p | 0 | 0 | 1 | 1 | 2 | 1 |
| hsa-mir-548q | 1 | 1 | 1 | 1 | 0 | 0 |
| hsa-mir-548s | 13 | 15 | 11 | 16 | 17 | 13 |
| hsa-mir-548t | 2 | 1 | 1 | 1 | 2 | 2 |
| hsa-mir-548u | 1 | 2 | 1 | 1 | 3 | 1 |
| hsa-mir-548v | 2 | 3 | 2 | 1 | 1 | 2 |
| hsa-mir-548w | 0 | 0 | 1 | 0 | 0 | 0 |
| hsa-mir-548x | 1 | 1 | 1 | 1 | 1 | 0 |
| hsa-mir-549 | 2 | 0 | 1 | 1 | 0 | 0 |
| hsa-mir-550-1 | 11 | 14 | 11 | 11 | 10 | 9 |
| hsa-mir-550-2 | 11 | 14 | 11 | 11 | 10 | 9 |
| hsa-mir-551a | 0 | 0 | 0 | 0 | 0 | 0 |
| hsa-mir-551b | 0 | 0 | 0 | 0 | 0 | 0 |
| hsa-mir-552 | 0 | 0 | 0 | 0 | 0 | 0 |
| hsa-mir-553 | 0 | 0 | 0 | 0 | 0 | 0 |
| hsa-mir-554 | 0 | 0 | 0 | 0 | 0 | 0 |
| hsa-mir-555 | 0 | 0 | 0 | 0 | 0 | 0 |
| hsa-mir-556 | 3 | 3 | 1 | 2 | 2 | 2 |
| hsa-mir-557 | 0 | 0 | 0 | 0 | 0 | 0 |
| hsa-mir-558 | 0 | 0 | 0 | 0 | 0 | 0 |
| hsa-mir-559 | 1 | 1 | 1 | 1 | 1 | 2 |
| hsa-mir-561 | 1 | 1 | 0 | 1 | 1 | 1 |
| hsa-mir-562 | 0 | 0 | 0 | 0 | 0 | 0 |
| hsa-mir-563 | 0 | 0 | 0 | 0 | 0 | 0 |
| hsa-mir-564 | 0 | 0 | 0 | 0 | 0 | 0 |
| hsa-mir-566 | 0 | 0 | 0 | 0 | 0 | 0 |
| hsa-mir-567 | 0 | 0 | 0 | 0 | 0 | 0 |
| hsa-mir-568 | 0 | 0 | 0 | 0 | 0 | 0 |
| hsa-mir-569 | 0 | 0 | 0 | 0 | 0 | 0 |
| hsa-mir-570 | 7 | 7 | 6 | 5 | 6 | 4 |
| hsa-mir-571 | 0 | 0 | 0 | 0 | 0 | 0 |
| hsa-mir-572 | 0 | 0 | 0 | 0 | 0 | 0 |
| hsa-mir-573 | 10 | 9 | 0 | 27 | 13 | 3 |
| hsa-mir-574 | 123 | 190 | 208 | 104 | 88 | 121 |
| hsa-mir-575 | 0 | 0 | 0 | 0 | 0 | 0 |
| hsa-mir-576 | 19 | 17 | 15 | 18 | 21 | 12 |
| hsa-mir-577 | 0 | 0 | 0 | 0 | 0 | 0 |
| hsa-mir-578 | 0 | 0 | 0 | 0 | 0 | 0 |
| hsa-mir-579 | 6 | 6 | 4 | 6 | 5 | 11 |
| hsa-mir-580 | 3 | 2 | 3 | 4 | 3 | 6 |
| hsa-mir-581 | 0 | 0 | 0 | 0 | 0 | 1 |
| hsa-mir-582 | 287 | 276 | 194 | 606 | 465 | 423 |
| hsa-mir-583 | 1 | 1 | 0 | 0 | 1 | 0 |
| hsa-mir-584 | 121 | 107 | 103 | 212 | 145 | 317 |
| hsa-mir-585 | 3 | 5 | 3 | 6 | 4 | 6 |
| hsa-mir-586 | 0 | 0 | 0 | 0 | 0 | 0 |
| hsa-mir-587 | 0 | 0 | 0 | 0 | 0 | 0 |
| hsa-mir-588 | 0 | 0 | 0 | 0 | 0 | 0 |
| hsa-mir-589 | 74 | 88 | 99 | 118 | 83 | 125 |

| | | | | | | |
|---|---|---|---|---|---|---|
| hsa-mir-590 | 312 | 300 | 251 | 125 | 145 | 143 |
| hsa-mir-591 | 0 | 0 | 0 | 0 | 0 | 0 |
| hsa-mir-592 | 0 | 0 | 0 | 0 | 0 | 0 |
| hsa-mir-593 | 0 | 0 | 0 | 0 | 0 | 0 |
| hsa-mir-595 | 0 | 0 | 0 | 0 | 0 | 0 |
| hsa-mir-596 | 0 | 0 | 0 | 0 | 0 | 0 |
| hsa-mir-597 | 2 | 3 | 2 | 1 | 1 | 2 |
| hsa-mir-598 | 208 | 214 | 167 | 436 | 279 | 359 |
| hsa-mir-599 | 0 | 0 | 0 | 0 | 0 | 0 |
| hsa-mir-600 | 0 | 0 | 0 | 0 | 0 | 0 |
| hsa-mir-601 | 0 | 0 | 0 | 0 | 0 | 0 |
| hsa-mir-602 | 0 | 0 | 0 | 0 | 0 | 0 |
| hsa-mir-603 | 1 | 0 | 0 | 1 | 0 | 0 |
| hsa-mir-604 | 0 | 0 | 0 | 0 | 0 | 0 |
| hsa-mir-605 | 0 | 0 | 0 | 0 | 0 | 0 |
| hsa-mir-606 | 0 | 0 | 0 | 0 | 0 | 0 |
| hsa-mir-607 | 0 | 0 | 0 | 1 | 1 | 1 |
| hsa-mir-608 | 0 | 0 | 0 | 0 | 0 | 0 |
| hsa-mir-609 | 0 | 0 | 0 | 0 | 0 | 0 |
| hsa-mir-610 | 0 | 0 | 0 | 0 | 0 | 0 |
| hsa-mir-611 | 0 | 0 | 0 | 0 | 0 | 0 |
| hsa-mir-612 | 0 | 1 | 0 | 1 | 1 | 1 |
| hsa-mir-613 | 0 | 0 | 0 | 0 | 0 | 0 |
| hsa-mir-614 | 0 | 0 | 0 | 0 | 0 | 0 |
| hsa-mir-615 | 30 | 48 | 32 | 30 | 32 | 18 |
| hsa-mir-616 | 1 | 1 | 1 | 1 | 1 | 1 |
| hsa-mir-617 | 0 | 0 | 0 | 0 | 0 | 0 |
| hsa-mir-618 | 0 | 0 | 0 | 0 | 0 | 0 |
| hsa-mir-619 | 0 | 0 | 0 | 0 | 0 | 0 |
| hsa-mir-620 | 0 | 0 | 0 | 0 | 0 | 0 |
| hsa-mir-621 | 0 | 0 | 0 | 0 | 0 | 0 |
| hsa-mir-622 | 0 | 0 | 0 | 0 | 0 | 0 |
| hsa-mir-623 | 0 | 0 | 0 | 0 | 0 | 0 |
| hsa-mir-624 | 12 | 11 | 12 | 8 | 7 | 7 |
| hsa-mir-625 | 50 | 48 | 41 | 26 | 20 | 19 |
| hsa-mir-626 | 0 | 0 | 0 | 0 | 0 | 0 |
| hsa-mir-627 | 85 | 57 | 58 | 77 | 38 | 165 |
| hsa-mir-628 | 3 | 4 | 3 | 5 | 5 | 6 |
| hsa-mir-629 | 24 | 28 | 20 | 37 | 32 | 27 |
| hsa-mir-630 | 0 | 0 | 0 | 0 | 0 | 0 |
| hsa-mir-631 | 0 | 0 | 0 | 0 | 0 | 0 |
| hsa-mir-632 | 0 | 0 | 0 | 0 | 0 | 0 |
| hsa-mir-633 | 0 | 0 | 0 | 0 | 0 | 0 |
| hsa-mir-634 | 0 | 0 | 0 | 0 | 0 | 0 |
| hsa-mir-635 | 0 | 0 | 0 | 0 | 0 | 0 |
| hsa-mir-636 | 1 | 1 | 0 | 0 | 0 | 0 |
| hsa-mir-637 | 0 | 0 | 0 | 0 | 0 | 0 |
| hsa-mir-638 | 0 | 0 | 0 | 0 | 0 | 0 |
| hsa-mir-639 | 0 | 0 | 0 | 0 | 0 | 0 |
| hsa-mir-640 | 0 | 0 | 0 | 0 | 0 | 0 |
| hsa-mir-641 | 4 | 6 | 4 | 2 | 3 | 1 |
| hsa-mir-642 | 2 | 2 | 4 | 1 | 2 | 2 |
| hsa-mir-643 | 2 | 2 | 1 | 2 | 1 | 1 |

| | | | | | | |
|---|---|---|---|---|---|---|
| hsa-mir-644 | 0 | 0 | 0 | 0 | 0 | 0 |
| hsa-mir-645 | 0 | 0 | 0 | 0 | 0 | 0 |
| hsa-mir-646 | 0 | 0 | 0 | 0 | 0 | 0 |
| hsa-mir-647 | 0 | 0 | 0 | 0 | 0 | 0 |
| hsa-mir-648 | 0 | 0 | 0 | 0 | 0 | 0 |
| hsa-mir-649 | 0 | 0 | 0 | 0 | 0 | 0 |
| hsa-mir-650 | 0 | 0 | 0 | 0 | 0 | 0 |
| hsa-mir-651 | 24 | 27 | 19 | 41 | 42 | 45 |
| hsa-mir-652 | 61 | 89 | 78 | 64 | 57 | 56 |
| hsa-mir-653 | 0 | 1 | 0 | 0 | 0 | 1 |
| hsa-mir-654 | 4 | 5 | 3 | 4 | 5 | 4 |
| hsa-mir-655 | 2 | 3 | 1 | 2 | 2 | 1 |
| hsa-mir-656 | 0 | 0 | 0 | 0 | 0 | 0 |
| hsa-mir-657 | 0 | 0 | 0 | 0 | 0 | 0 |
| hsa-mir-658 | 0 | 0 | 0 | 0 | 0 | 0 |
| hsa-mir-659 | 2 | 1 | 1 | 3 | 2 | 4 |
| hsa-mir-660 | 25 | 28 | 29 | 28 | 30 | 38 |
| hsa-mir-661 | 0 | 0 | 0 | 0 | 0 | 0 |
| hsa-mir-662 | 0 | 0 | 0 | 0 | 0 | 0 |
| hsa-mir-663 | 2 | 4 | 1 | 3 | 2 | 6 |
| hsa-mir-663b | 2 | 3 | 1 | 4 | 2 | 1 |
| hsa-mir-664 | 11 | 11 | 12 | 11 | 12 | 13 |
| hsa-mir-665 | 0 | 0 | 0 | 0 | 0 | 0 |
| hsa-mir-668 | 0 | 0 | 0 | 0 | 0 | 0 |
| hsa-mir-670 | 0 | 0 | 0 | 0 | 0 | 0 |
| hsa-mir-671 | 27 | 23 | 27 | 28 | 44 | 79 |
| hsa-mir-675 | 0 | 0 | 0 | 0 | 0 | 0 |
| hsa-mir-7-1 | 196 | 242 | 151 | 1825 | 2960 | 1080 |
| hsa-mir-7-2 | 169 | 207 | 132 | 1790 | 2924 | 1059 |
| hsa-mir-7-3 | 169 | 207 | 132 | 1789 | 2923 | 1059 |
| hsa-mir-708 | 1236 | 1100 | 1145 | 582 | 584 | 736 |
| hsa-mir-711 | 0 | 0 | 0 | 0 | 0 | 0 |
| hsa-mir-718 | 0 | 0 | 0 | 0 | 0 | 0 |
| hsa-mir-720 | 54 | 75 | 42 | 43 | 42 | 79 |
| hsa-mir-744 | 170 | 218 | 206 | 188 | 156 | 199 |
| hsa-mir-758 | 0 | 1 | 0 | 0 | 0 | 0 |
| hsa-mir-759 | 0 | 0 | 0 | 0 | 0 | 0 |
| hsa-mir-760 | 1 | 1 | 1 | 1 | 1 | 1 |
| hsa-mir-761 | 0 | 0 | 0 | 0 | 0 | 0 |
| hsa-mir-762 | 0 | 0 | 0 | 0 | 0 | 0 |
| hsa-mir-764 | 0 | 0 | 0 | 0 | 0 | 0 |
| hsa-mir-765 | 0 | 0 | 0 | 0 | 0 | 0 |
| hsa-mir-766 | 2 | 4 | 3 | 6 | 5 | 4 |
| hsa-mir-767 | 0 | 0 | 0 | 0 | 0 | 0 |
| hsa-mir-769 | 172 | 162 | 164 | 206 | 217 | 209 |
| hsa-mir-770 | 0 | 0 | 0 | 0 | 0 | 0 |
| hsa-mir-802 | 0 | 0 | 0 | 0 | 0 | 0 |
| hsa-mir-873 | 0 | 0 | 0 | 1 | 1 | 1 |
| hsa-mir-874 | 6 | 13 | 11 | 12 | 11 | 10 |
| hsa-mir-875 | 0 | 0 | 0 | 0 | 0 | 0 |
| hsa-mir-876 | 0 | 0 | 0 | 1 | 0 | 0 |
| hsa-mir-877 | 119 | 129 | 63 | 133 | 110 | 105 |
| hsa-mir-885 | 0 | 0 | 0 | 0 | 0 | 0 |

| | | | | | | |
|---|---|---|---|---|---|---|
| hsa-mir-886 | 329 | 373 | 310 | 781 | 719 | 515 |
| hsa-mir-887 | 1 | 1 | 0 | 1 | 0 | 1 |
| hsa-mir-888 | 0 | 0 | 0 | 0 | 0 | 0 |
| hsa-mir-889 | 1 | 1 | 1 | 2 | 1 | 4 |
| hsa-mir-890 | 0 | 0 | 0 | 0 | 0 | 0 |
| hsa-mir-891a | 0 | 0 | 0 | 1 | 0 | 1 |
| hsa-mir-891b | 0 | 0 | 0 | 0 | 0 | 0 |
| hsa-mir-892a | 0 | 0 | 0 | 0 | 0 | 0 |
| hsa-mir-892b | 0 | 0 | 0 | 0 | 0 | 0 |
| hsa-mir-9-1 | 4 | 4 | 3 | 5 | 6 | 9 |
| hsa-mir-9-2 | 4 | 4 | 3 | 5 | 6 | 9 |
| hsa-mir-9-3 | 4 | 4 | 3 | 6 | 6 | 9 |
| hsa-mir-920 | 0 | 0 | 0 | 0 | 0 | 0 |
| hsa-mir-921 | 0 | 0 | 0 | 0 | 0 | 0 |
| hsa-mir-922 | 0 | 0 | 0 | 0 | 0 | 0 |
| hsa-mir-924 | 0 | 0 | 0 | 0 | 0 | 0 |
| hsa-mir-92a-1 | 1648 | 2168 | 2211 | 2420 | 2149 | 2530 |
| hsa-mir-92a-2 | 1643 | 2159 | 2208 | 2412 | 2142 | 2526 |
| hsa-mir-92b | 50 | 79 | 67 | 71 | 63 | 63 |
| hsa-mir-93 | 1194 | 1360 | 1076 | 2168 | 1739 | 2206 |
| hsa-mir-933 | 0 | 0 | 0 | 0 | 0 | 0 |
| hsa-mir-934 | 0 | 0 | 1 | 5 | 5 | 9 |
| hsa-mir-935 | 0 | 0 | 0 | 0 | 0 | 0 |
| hsa-mir-936 | 1 | 1 | 0 | 1 | 1 | 0 |
| hsa-mir-937 | 0 | 0 | 0 | 1 | 1 | 0 |
| hsa-mir-938 | 0 | 0 | 0 | 0 | 0 | 0 |
| hsa-mir-939 | 0 | 1 | 0 | 1 | 0 | 1 |
| hsa-mir-940 | 5 | 10 | 6 | 4 | 5 | 4 |
| hsa-mir-941-1 | 48 | 66 | 55 | 146 | 140 | 92 |
| hsa-mir-941-2 | 89 | 123 | 106 | 279 | 270 | 178 |
| hsa-mir-941-3 | 85 | 119 | 106 | 273 | 261 | 175 |
| hsa-mir-942 | 5 | 8 | 4 | 9 | 5 | 4 |
| hsa-mir-943 | 0 | 0 | 0 | 0 | 0 | 0 |
| hsa-mir-944 | 288 | 239 | 188 | 545 | 429 | 604 |
| hsa-mir-95 | 1 | 0 | 1 | 1 | 1 | 1 |
| hsa-mir-96 | 1267 | 1872 | 1682 | 664 | 709 | 518 |
| hsa-mir-98 | 353 | 399 | 292 | 290 | 422 | 223 |
| hsa-mir-99a | 3903 | 3588 | 3309 | 4113 | 2552 | 5307 |
| hsa-mir-99b | 1123 | 1384 | 1210 | 3017 | 2113 | 2397 |
