# Supplementary material for: Genome-Wide Small RNA Sequencing and Gene Expression Analysis Reveals a microRNA Profile of Cancer Susceptibility in ATM-Deficient Human Mammary Epithelial Cells
Source: PLoS One. 2013 May 31;8(5):e64779. doi: 10.1371/journal.pone.0064779 (PMC3669333; doi:10.1371/journal.pone.0064779)
Supplement: Table S2 — Sequence count for 259 present miRNAs. Sequence count for the 259 miRNAs deemed present (above 10 TpM) in either 2 out of 3 wild type replicates or 2 out of 3 ATM-deficient replicates. (PDF) [file pone.0064779.s002.pdf]

miRNA ID	WT1-TpM	WT2- TpM	WT3- TpM	ATM1- TpM	ATM2- TpM	ATM3- TpM
hsa-mir-338	39	37	37	71	53	102
hsa-mir-720	54	75	42	43	42	79
hsa-mir-196b	64	65	57	88	88	96
hsa-mir-181c	71	59	34	146	78	120
hsa-mir-1285-2	71	83	61	159	160	81
hsa-mir-1285-1	74	85	63	162	162	84
hsa-mir-589	74	88	99	118	83	125
hsa-mir-532	77	90	95	104	86	125
hsa-mir-627	85	57	58	77	38	165
hsa-mir-941-3	85	119	106	273	261	175
hsa-mir-335	86	87	68	144	148	162
hsa-mir-941-2	89	123	106	279	270	178
hsa-mir-454	108	134	98	140	158	112
hsa-mir-196a-1	113	124	123	137	132	149
hsa-mir-1277	118	128	82	30	34	48
hsa-mir-192	118	102	106	288	245	364
hsa-mir-197	119	177	172	164	143	90
hsa-mir-877	119	129	63	133	110	105
hsa-mir-224	120	140	85	121	100	84
hsa-mir-584	121	107	103	212	145	317
hsa-mir-574	123	190	208	104	88	121
hsa-mir-10a	129	122	132	136	85	164
hsa-mir-196a-2	138	154	150	164	156	178
hsa-mir-200b	142	129	143	223	190	244
hsa-mir-331	148	156	151	155	133	182
hsa-mir-4306	153	117	83	88	104	85
hsa-mir-185	157	124	87	92	108	92
hsa-mir-361	162	128	138	146	127	184
hsa-mir-1307	165	333	248	459	330	806
hsa-mir-301a	167	232	151	96	165	58
hsa-mir-340	168	135	89	102	153	77
hsa-mir-7-2	169	207	132	1790	2924	1059
hsa-mir-7-3	169	207	132	1789	2923	1059
hsa-mir-342	169	258	239	164	140	138
hsa-mir-542	170	139	145	210	214	313
hsa-mir-744	170	218	206	188	156	199
hsa-mir-769	172	162	164	206	217	209
hsa-mir-365-2	176	173	173	209	207	302
hsa-mir-365-1	179	178	175	215	214	308
hsa-mir-203	191	160	350	961	528	3645
hsa-mir-330	196	179	167	60	55	51
hsa-mir-7-1	196	242	151	1825	2960	1080
hsa-mir-339	202	243	261	172	145	179
hsa-mir-125a	206	336	331	297	266	313
hsa-mir-598	208	214	167	436	279	359
hsa-mir-138-2	211	254	193	184	163	160
hsa-mir-152	216	209	262	358	298	443
hsa-mir-181b-2	216	235	205	317	261	507
hsa-mir-1259	223	214	197	124	76	237
hsa-mir-181b-1	223	241	206	332	271	513
hsa-mir-138-1	234	280	212	192	170	166
hsa-mir-455	250	310	203	301	268	167
hsa-mir-503	257	293	372	224	255	304
hsa-mir-582	287	276	194	606	465	423

hsa-mir-944	288	239	188	545	429	604
hsa-mir-137	292	242	279	169	95	279
hsa-mir-146a	296	253	234	460	370	423
hsa-mir-146b	297	253	234	462	372	425
hsa-mir-1975	302	473	314	347	259	367
hsa-mir-590	312	300	251	125	145	143
hsa-mir-374b	326	275	209	207	204	146
hsa-mir-130b	328	382	264	180	356	132
hsa-mir-886	329	373	310	781	719	515
hsa-mir-98	353	399	292	290	422	223
hsa-mir-452	365	309	205	407	498	430
hsa-mir-125b-2	378	611	612	589	514	808
hsa-mir-25	384	437	389	476	462	436
hsa-mir-181a-2	420	482	524	600	455	721
hsa-mir-125b-1	435	673	636	616	549	842
hsa-mir-181a-1	438	485	508	611	431	760
hsa-mir-28	444	492	442	547	526	464
hsa-mir-186	507	465	374	613	398	854
hsa-mir-32	560	933	582	405	703	261
hsa-mir-34a	565	579	719	645	694	821
hsa-mir-320e	575	565	430	451	573	334
hsa-mir-320d-1	587	577	438	457	586	340
hsa-mir-320d-2	587	577	438	457	586	340
hsa-mir-320c-2	594	580	442	458	587	346
hsa-mir-320c-1	594	580	442	458	587	346
hsa-mir-320b-1	594	580	443	459	587	347
hsa-mir-320b-2	594	580	443	458	587	347
hsa-mir-320a	596	582	445	461	589	354
hsa-mir-210	602	897	748	647	669	917
hsa-mir-193b	673	1048	1059	766	633	786
hsa-mir-15b	676	868	711	680	827	502
hsa-mir-140	732	710	694	938	699	906
hsa-mir-22	779	950	1021	672	694	1194
hsa-mir-33a	795	942	898	170	177	269
hsa-mir-183	821	880	843	2244	1716	1574
hsa-mir-3184	829	855	735	1008	1263	891
hsa-mir-423	829	855	735	1008	1263	891
hsa-mir-424	845	1107	1237	441	820	518
hsa-mir-19a	885	975	727	287	341	282
hsa-mir-15a	1018	1068	828	762	819	977
hsa-let-7d	1079	1125	1009	968	1121	620
hsa-mir-99b	1123	1384	1210	3017	2113	2397
hsa-mir-1297	1136	1139	949	681	881	613
hsa-mir-26b	1150	1157	963	692	894	626
hsa-mir-93	1194	1360	1076	2168	1739	2206
hsa-mir-708	1236	1100	1145	582	584	736
hsa-let-7e	1260	1108	774	1219	1552	641
hsa-mir-96	1267	1872	1682	664	709	518
hsa-mir-130a	1321	1670	1383	855	1132	851
hsa-mir-191	1327	1336	1094	2316	1980	2073
hsa-mir-1308	1422	7661	804	13756	38192	797
hsa-mir-148b	1474	1520	1272	5618	4907	4310
hsa-mir-135b	1546	2136	1821	1119	1409	765
hsa-mir-92a-2	1643	2159	2208	2412	2142	2526
hsa-mir-92a-1	1648	2168	2211	2420	2149	2530

hsa-mir-26a-1	1839	1811	1933	1345	1789	1235
hsa-mir-26a-2	1848	1820	1945	1357	1814	1254
hsa-mir-374a	1878	1733	1021	4066	4178	2416
hsa-mir-18b	1977	1951	1529	1407	1079	1740
hsa-mir-18a	1997	1976	1545	1420	1095	1754
hsa-mir-425	2030	2460	2033	1405	1234	761
hsa-mir-19b-2	2241	2986	2073	1589	1829	1066
hsa-mir-19b-1	2270	3016	2079	1599	1836	1105
hsa-mir-221	2412	3020	2265	4663	4237	4295
hsa-mir-1826	2675	3559	3891	2250	1903	3937
hsa-mir-29a	2718	3289	2796	2248	1872	3267
hsa-mir-222	2748	3156	2123	1999	1470	2122
hsa-mir-16-1	2888	4248	3444	1932	2643	1306
hsa-mir-16-2	2918	4280	3467	1962	2676	1331
hsa-mir-30d	3071	3002	3110	8232	6210	7937
hsa-mir-151	3156	2960	2693	6983	4951	5879
hsa-mir-34c	3631	4145	3437	4564	5094	2699
hsa-mir-106b	3827	3089	2445	3195	2240	4787
hsa-mir-99a	3903	3588	3309	4113	2552	5307
hsa-mir-30c-1	3958	4489	4161	3729	3661	2930
hsa-mir-30b	3965	4494	4163	3730	3663	2932
hsa-mir-30c-2	3972	4500	4171	3760	3680	2954
hsa-mir-100	4103	3753	3364	4165	2613	5374
hsa-mir-148a	4112	3795	4989	13646	11379	14487
hsa-mir-17	4238	4280	3488	3626	3013	3404
hsa-mir-182	4673	5149	5857	11123	7641	6243
hsa-mir-205	4914	7561	8260	6065	6156	7833
hsa-mir-101-2	5595	5716	5705	12807	12116	14022
hsa-mir-101-1	5597	5718	5707	12808	12117	14022
hsa-let-7i	5730	6061	5584	4832	5468	3188
hsa-let-7g	5775	6073	5625	4827	5460	3276
hsa-mir-200c	6619	5811	5712	9377	9201	12495
hsa-mir-30e	7059	6337	8005	10059	8490	13333
hsa-mir-30a	7206	6762	7761	23491	16662	21598
hsa-mir-20a	8701	7874	7190	7470	6064	9208
hsa-mir-29c	9387	11792	10858	2809	3283	2819
hsa-mir-29b-2	9391	11791	10877	2807	3281	2850
hsa-mir-29b-1	9417	11816	10889	2824	3297	2929
hsa-mir-107	9857	8157	7624	14928	14363	16078
hsa-mir-103-1	9863	8154	7631	14930	14362	16131
hsa-mir-103-1-as	9863	8154	7631	14930	14362	16131
hsa-mir-103-2-as	9878	8170	7638	14944	14376	16141
hsa-mir-103-2	9899	8193	7643	14952	14385	16147
hsa-mir-23b	13088	11122	10383	17542	13811	11879
hsa-mir-23a	13117	11145	10395	17573	13841	11918
hsa-let-7f-2	20903	20183	18486	22093	29241	16649
hsa-let-7f-1	20941	20208	18522	22124	29249	16706
hsa-let-7c	21011	19761	20450	26440	26902	24473
hsa-let-7a-2	21109	19815	20514	26499	26938	24799
hsa-let-7b	21264	19975	20654	26757	27102	24962
hsa-let-7a-1	21315	20001	20740	26721	27131	25236
hsa-let-7a-3	21371	20045	20774	26817	27194	25335
hsa-mir-200a	24447	23238	21066	8036	8103	11810
hsa-mir-141	24560	23336	21130	8148	8210	11984
hsa-mir-27b	25691	27069	27856	24868	23300	29664

hsa-mir-27a	26063	27539	28150	25368	23722	30321
hsa-mir-378c	35117	37996	25337	64398	80177	25205
hsa-mir-378	35123	38004	25346	64406	80185	25212
hsa-mir-31	62522	77945	73235	8066	9364	4568
hsa-mir-24-2	72400	63966	75996	27885	29251	33051
hsa-mir-24-1	72404	63966	75998	27885	29248	33051
hsa-mir-3074	72404	63966	75998	27885	29248	33051
hsa-mir-21	108466	104005	129377	145234	102139	234270
hsa-mir-1979	10	21	8	34	41	10
hsa-mir-2110	4	6	5	13	14	13
hsa-mir-379	7	9	9	17	20	18
hsa-mir-450b	8	9	10	14	11	34
hsa-mir-548e	9	9	6	16	23	19
hsa-mir-548g	2	1	1	11	11	5
hsa-mir-573	10	9	0	27	13	3
hsa-mir-874	6	13	11	12	11	10
hsa-mir-129-1	11	14	14	32	31	44
hsa-mir-1305	11	11	9	9	7	32
hsa-mir-129-2	11	14	13	32	31	44
hsa-mir-550-1	11	14	11	11	10	9
hsa-mir-550-2	11	14	11	11	10	9
hsa-mir-1292	11	13	9	24	16	17
hsa-mir-139	11	14	10	13	9	7
hsa-mir-381	11	10	15	15	14	18
hsa-mir-548o	11	13	10	15	17	12
hsa-mir-664	11	11	12	11	12	13
hsa-mir-450a-2	12	18	17	20	19	25
hsa-mir-450a-1	12	18	17	20	19	25
hsa-mir-624	12	11	12	8	7	7
hsa-mir-142	13	12	11	6	5	5
hsa-mir-376a-1	13	11	12	9	6	17
hsa-mir-1255a	13	8	8	11	13	8
hsa-mir-548s	13	15	11	16	17	13
hsa-mir-3172	14	28	7	38	29	29
hsa-mir-218-1	14	19	15	55	66	41
hsa-mir-218-2	15	20	15	54	65	41
hsa-mir-143	15	15	16	19	27	14
hsa-mir-193a	15	31	27	8	12	8
hsa-mir-548f-3	15	15	6	41	41	21
hsa-mir-548f-4	15	15	6	41	41	21
hsa-mir-548f-2	16	16	6	41	41	22
hsa-mir-1293	16	19	9	25	24	19
hsa-mir-3195	17	43	16	34	24	22
hsa-mir-188	18	20	20	12	12	23
hsa-mir-301b	19	33	20	10	22	7
hsa-mir-3178	19	25	20	4	3	78
hsa-mir-106a	19	19	19	50	44	57
hsa-mir-1268	19	32	10	22	35	26
hsa-mir-576	19	17	15	18	21	12
hsa-mir-362	20	25	20	17	18	18
hsa-mir-4284	21	37	9	27	22	13
hsa-mir-3158-1	22	19	19	33	33	44
hsa-mir-3158-2	22	19	19	33	33	44
hsa-mir-126	23	27	27	18	17	25
hsa-mir-421	23	24	15	17	20	12

hsa-mir-629	24	28	20	37	32	27
hsa-mir-651	24	27	19	41	42	45
hsa-mir-2355	25	25	14	29	23	30
hsa-mir-660	25	28	29	28	30	38
hsa-mir-502	25	41	23	35	32	32
hsa-mir-3182	27	29	26	50	44	56
hsa-mir-671	27	23	27	28	44	79
hsa-mir-1296	28	56	39	33	32	20
hsa-mir-190b	29	26	19	31	30	30
hsa-mir-190	30	26	20	31	31	30
hsa-mir-500	30	45	27	41	35	37
hsa-mir-615	30	48	32	30	32	18
hsa-mir-1201	31	38	37	57	47	75
hsa-mir-132	31	42	23	38	30	32
hsa-mir-1260b	35	48	43	48	39	86
hsa-mir-484	37	50	41	43	39	32
hsa-mir-3123	37	82	13	48	111	25
hsa-mir-505	37	46	34	45	33	24
hsa-mir-429	38	34	35	67	50	98
hsa-mir-3065	39	37	37	71	53	102
hsa-mir-548f-1	44	37	23	72	66	65
hsa-mir-1280	45	65	50	23	24	105
hsa-mir-1275	47	38	38	56	39	72
hsa-mir-941-1	48	66	55	146	140	92
hsa-mir-215	50	41	27	83	82	78
hsa-mir-92b	50	79	67	71	63	63
hsa-mir-625	50	48	41	26	20	19
hsa-mir-1274b	51	52	25	20	17	34
hsa-mir-10b	58	57	62	124	121	102
hsa-mir-324	59	72	67	59	47	53
hsa-mir-194-1	59	45	48	43	34	61
hsa-mir-194-2	60	46	48	44	34	62
hsa-mir-345	61	49	41	30	18	23
hsa-mir-652	61	89	78	64	57	56
hsa-mir-34b	67	72	59	46	44	45
hsa-mir-128-2	77	72	59	83	79	75
hsa-mir-128-1	77	72	59	84	81	76
hsa-mir-1246	83	55	26	56	92	86
hsa-mir-149	87	120	74	118	111	38
hsa-mir-33b	269	398	453	50	54	99
