# Supplementary material for: Genome-Wide Small RNA Sequencing and Gene Expression Analysis Reveals a microRNA Profile of Cancer Susceptibility in ATM-Deficient Human Mammary Epithelial Cells
Source: PLoS One. 2013 May 31;8(5):e64779. doi: 10.1371/journal.pone.0064779 (PMC3669333; doi:10.1371/journal.pone.0064779)
Supplement: Table S3 — 81 ATM-dependent miRNAs. 81 microRNAs determined to have a significant change in expression in the ATM-deficient cells compared to wild type controls. T-test p≤0.05; Fold Change +/−1.5 or greater. (PDF) [file pone.0064779.s003.pdf]

miRNA ID	Average WT TpM	Average ATM TpM	p-value	Fold Change
hsa-mir-101-1	5674	12982	0.0002	2.29
hsa-mir-101-2	5672	12982	0.0002	2.29
hsa-mir-103-1	8549	15141	0.0015	1.77
hsa-mir-103-1-as	8549	15141	0.0015	1.77
hsa-mir-103-2	8578	15161	0.0015	1.77
hsa-mir-103-2-as	8562	15153	0.0015	1.77
hsa-mir-106a	19	50	0.0013	2.66
hsa-mir-107	8546	15123	0.0014	1.77
hsa-mir-10b	59	115	0.0014	1.96
hsa-mir-1201	35	60	0.0432	1.70
hsa-mir-1277	109	37	0.0091	-2.94
hsa-mir-129-1	13	36	0.0055	2.77
hsa-mir-129-2	13	36	0.0050	2.79
hsa-mir-1292	11	19	0.0466	1.73
hsa-mir-130a	1458	946	0.0227	-1.54
hsa-mir-135b	1834	1098	0.0435	-1.67
hsa-mir-141	23009	9447	0.0011	-2.44
hsa-mir-146a	261	418	0.0081	1.60
hsa-mir-146b	262	420	0.0078	1.60
hsa-mir-148a	4299	13171	0.0009	3.06
hsa-mir-148b	1422	4945	0.0008	3.48
hsa-mir-151	2936	5937	0.0076	2.02
hsa-mir-152	229	366	0.0387	1.60
hsa-mir-16-1	3527	1960	0.0470	-1.80
hsa-mir-16-2	3555	1990	0.0477	-1.79
hsa-mir-183	848	1845	0.0082	2.18
hsa-mir-191	1252	2123	0.0024	1.70
hsa-mir-192	109	299	0.0057	2.75
hsa-mir-193a	25	9	0.0392	-2.63
hsa-mir-19a	862	304	0.0017	-2.84
hsa-mir-200a	22917	9316	0.0010	-2.46
hsa-mir-200b	138	219	0.0076	1.59
hsa-mir-200c	6047	10358	0.0177	1.71
hsa-mir-215	39	81	0.0037	2.07
hsa-mir-218-1	16	54	0.0068	3.33
hsa-mir-218-2	16	54	0.0070	3.25
hsa-mir-221	2565	4398	0.0024	1.71
hsa-mir-24-1	70789	30061	0.0005	-2.35
hsa-mir-24-2	70787	30062	0.0005	-2.35
hsa-mir-29b-1	10707	3016	0.0004	-3.55
hsa-mir-29b-2	10687	2979	0.0004	-3.59
hsa-mir-29c	10679	2970	0.0004	-3.60
hsa-mir-3074	70789	30061	0.0005	-2.35
hsa-mir-30a	7243	20584	0.0029	2.84
hsa-mir-30d	3061	7460	0.0022	2.44
hsa-mir-31	71234	7333	0.0002	-9.71
hsa-mir-3158-1	20	37	0.0108	1.87
hsa-mir-3158-2	20	37	0.0108	1.87

hsa-mir-3182	27	50	0.0032	1.83
hsa-mir-330	180	55	0.0001	-3.25
hsa-mir-335	80	151	0.0010	1.88
hsa-mir-33a	879	205	0.0002	-4.28
hsa-mir-33b	373	68	0.0058	-5.50
hsa-mir-345	50	24	0.0187	-2.11
hsa-mir-374a	1544	3553	0.0330	2.30
hsa-mir-379	8	18	0.0005	2.20
hsa-mir-424	1063	593	0.0451	-1.79
hsa-mir-425	2174	1133	0.0122	-1.92
hsa-mir-452	293	445	0.0484	1.52
hsa-mir-548e	8	19	0.0090	2.38
hsa-mir-548f-1	35	68	0.0067	1.95
hsa-mir-548f-2	13	35	0.0378	2.77
hsa-mir-548f-3	12	34	0.0351	2.88
hsa-mir-548f-4	12	34	0.0356	2.86
hsa-mir-582	252	498	0.0173	1.97
hsa-mir-590	287	137	0.0016	-2.09
hsa-mir-598	196	358	0.0276	1.82
hsa-mir-625	46	21	0.0018	-2.19
hsa-mir-651	24	43	0.0016	1.80
hsa-mir-7-1	196	1955	0.0325	9.96
hsa-mir-7-2	169	1924	0.0319	11.36
hsa-mir-7-3	169	1924	0.0319	11.36
hsa-mir-708	1160	634	0.0012	-1.83
hsa-mir-886	337	672	0.0154	1.99
hsa-mir-93	1210	2037	0.0084	1.68
hsa-mir-941-1	56	126	0.0181	2.24
hsa-mir-941-2	106	242	0.0154	2.29
hsa-mir-941-3	104	237	0.0146	2.28
hsa-mir-944	238	526	0.0082	2.21
hsa-mir-96	1607	630	0.0065	-2.55
hsa-mir-99b	1239	2509	0.0103	2.02
