# Supplementary material for: Genome-Wide Small RNA Sequencing and Gene Expression Analysis Reveals a microRNA Profile of Cancer Susceptibility in ATM-Deficient Human Mammary Epithelial Cells
Source: PLoS One. 2013 May 31;8(5):e64779. doi: 10.1371/journal.pone.0064779 (PMC3669333; doi:10.1371/journal.pone.0064779)
Supplement: Table S4 — 1086 ATM-dependent mRNAs. 1086 mRNA probes determined to have significant expression changes in ATM-deficient cells compared to wild-type control cells. T-test p≤0.05; Fold Change +/−1.5 or greater. (PDF) [file pone.0064779.s004.pdf]

Probeset ID	GeneName	p-value	Fold Change
A_32_P29140	AA344632	0.0146	-2.90
A_32_P40424	AA630774	0.0024	-1.55
A_23_P152505	ABAT	0.0098	2.11
A_23_P158976	ABCC2	0.0495	1.77
A_24_P142141	ABHD2	0.0098	-3.22
A_23_P305759	ABHD3	0.0367	-2.07
A_23_P256205	ABLIM3	0.0311	1.60
A_23_P107166	ACBD4	0.0305	1.51
A_23_P316381	ACOX3	0.0271	1.53
A_23_P120594	ACSS1	0.0333	3.35
A_24_P276947	ACTR2	0.0127	-1.79
A_23_P137786	ADAMTSL4	0.0114	2.21
A_23_P391938	ADAMTSL4	0.0146	2.51
A_24_P416177	ADCY7	0.0339	-1.89
A_24_P302406	AF086286	0.0107	-1.53
A_24_P652510	AF086529	0.0348	-1.71
A_24_P915361	AF086536	0.0191	1.66
A_23_P256903	AF086546	0.0079	-1.95
A_24_P933138	AF087999	0.0289	1.61
A_32_P99171	AF131762	0.0177	-2.48
A_24_P298495	AF289590	0.0028	1.75
A_23_P355289	AFG3L1	0.0393	1.58
A_32_P167705	AGBL2	0.0079	1.99
A_23_P356466	AGPAT3	0.0037	1.98
A_23_P344400	AHRR	0.0121	-2.39
A_32_P190222	AI167420	0.0129	-1.52
A_32_P180538	AI354226	0.0036	-1.62
A_24_P683905	AK001829	0.0267	1.71
A_24_P269006	AK021800	0.0121	1.53
A_24_P642758	AK022479	0.0059	1.98
A_32_P83256	AK023663	0.0007	-3.39
A_32_P387905	AK024224	0.0022	-2.52
A_24_P84370	AK024470	0.0438	2.39
A_24_P133171	AK026078	0.0427	-1.83
A_32_P20703	AK026418	0.0484	2.09
A_23_P399292	AK027091	0.0246	2.40
A_24_P606239	AK056073	0.0370	1.84
A_32_P190049	AK056809	0.0320	-1.62
A_32_P92281	AK091784	0.0090	1.60
A_24_P256404	AK093202	0.0334	1.65
A_32_P24741	AK093729	0.0051	2.12

A_32_P41375	AK093729	0.0081	2.28
A_32_P50406	AK093729	0.0132	2.13
A_32_P204330	AK093982	0.0071	1.64
A_24_P397903	AK094296	0.0083	-1.83
A_32_P213615	AK096778	0.0466	2.41
A_23_P315252	AK097322	0.0058	1.54
A_32_P51518	AK098220	0.0461	2.48
A_24_P367576	AK125170	0.0220	1.56
A_24_P714134	AK126814	0.0408	-1.92
A_32_P188752	AK129584	0.0193	1.53
A_23_P418477	AKAP11	0.0340	1.72
A_24_P164337	AKAP13	0.0265	-1.54
A_24_P312578	AKR1C1	0.0024	-1.53
A_32_P155841	AL079294	0.0237	1.70
A_23_P205959	ALDH1A3	0.0462	-1.85
A_23_P207213	ALDH3A1	0.0180	-1.78
A_23_P137116	AMMECR1	0.0126	-2.02
A_23_P257164	AMT	0.0244	1.59
A_24_P220771	ANKRD17	0.0212	-1.51
A_23_P161428	ANKRD22	0.0357	2.51
A_32_P51237	ANKRD38	0.0363	3.93
A_24_P345846	ANTXR2	0.0192	-1.95
A_32_P148345	ANXA2	0.0004	-1.55
A_23_P395054	ANXA8	0.0148	1.80
A_23_P103617	ANXA9	0.0276	2.04
A_23_P154037	AOX1	0.0079	-1.57
A_24_P190804	AP1S2	0.0153	-2.01
A_23_P217384	AP1S2	0.0371	-1.83
A_23_P29237	APOL3	0.0304	2.98
A_23_P95050	ARFIP1	0.0105	-1.93
A_24_P920207	ARHGAP23	0.0397	1.81
A_23_P332326	ARHGEF19	0.0454	2.19
A_23_P98252	ARL2	0.0112	-1.70
A_23_P129466	ATF7IP2	0.0355	2.34
A_23_P35916	ATM	0.0005	-2.98
A_23_P157478	ATP6V1H	0.0437	-1.79
A_32_P127501	AV753543	0.0234	1.91
A_32_P42895	AW138903	0.0170	2.34
A_32_P106615	AW268902	0.0157	3.93
A_32_P146169	AW673984	0.0004	-1.50
A_32_P128588	AW851396	0.0145	-1.60
A_24_P810735	AX721128	0.0296	-1.66

A_23_P136857	A_23_P136857	0.0141	-1.65
A_23_P140454	A_23_P140454	0.0438	1.51
A_23_P251196	A_23_P251196	0.0115	1.69
A_23_P300563	A_23_P300563	0.0056	-1.53
A_23_P75129	A_23_P75129	0.0021	-1.77
A_24_P101211	A_24_P101211	0.0223	-1.58
A_24_P110201	A_24_P110201	0.0197	-1.59
A_24_P118411	A_24_P118411	0.0038	-2.45
A_24_P118953	A_24_P118953	0.0175	-1.97
A_24_P127042	A_24_P127042	0.0319	-1.87
A_24_P127362	A_24_P127362	0.0306	-1.54
A_24_P136155	A_24_P136155	0.0047	1.91
A_24_P144314	A_24_P144314	0.0286	-1.93
A_24_P144337	A_24_P144337	0.0473	-1.52
A_24_P152278	A_24_P152278	0.0407	-1.79
A_24_P169903	A_24_P169903	0.0250	-2.07
A_24_P196019	A_24_P196019	0.0098	-1.56
A_24_P196134	A_24_P196134	0.0499	-1.75
A_24_P213256	A_24_P213256	0.0332	-1.69
A_24_P221475	A_24_P221475	0.0239	-1.77
A_24_P230388	A_24_P230388	0.0050	-1.95
A_24_P234871	A_24_P234871	0.0006	2.41
A_24_P237820	A_24_P237820	0.0332	-2.14
A_24_P247576	A_24_P247576	0.0062	-1.55
A_24_P24890	A_24_P24890	0.0371	-1.74
A_24_P255123	A_24_P255123	0.0173	-1.95
A_24_P256050	A_24_P256050	0.0092	-2.13
A_24_P272653	A_24_P272653	0.0296	-1.61
A_24_P289043	A_24_P289043	0.0073	-1.72
A_24_P289504	A_24_P289504	0.0179	-1.70
A_24_P290188	A_24_P290188	0.0329	-1.53
A_24_P298099	A_24_P298099	0.0091	-1.58
A_24_P298616	A_24_P298616	0.0204	-1.65
A_24_P321184	A_24_P321184	0.0176	-1.76
A_24_P324506	A_24_P324506	0.0224	-1.57
A_24_P324538	A_24_P324538	0.0043	-1.77
A_24_P332721	A_24_P332721	0.0331	-2.13
A_24_P333052	A_24_P333052	0.0146	-2.31
A_24_P341731	A_24_P341731	0.0115	-1.51
A_24_P358337	A_24_P358337	0.0481	-1.55
A_24_P366465	A_24_P366465	0.0148	-2.09
A_24_P366768	A_24_P366768	0.0270	-1.73

A_24_P367249	A_24_P367249	0.0239	-1.53
A_24_P375573	A_24_P375573	0.0048	-1.77
A_24_P383802	A_24_P383802	0.0308	-1.60
A_24_P384196	A_24_P384196	0.0072	-1.52
A_24_P392622	A_24_P392622	0.0490	-1.92
A_24_P400616	A_24_P400616	0.0345	-1.53
A_24_P409440	A_24_P409440	0.0044	-1.64
A_24_P41662	A_24_P41662	0.0233	-1.51
A_24_P50281	A_24_P50281	0.0178	-2.14
A_24_P50381	A_24_P50381	0.0217	-2.17
A_24_P518369	A_24_P518369	0.0141	-2.31
A_24_P521544	A_24_P521544	0.0205	-1.67
A_24_P524164	A_24_P524164	0.0368	-3.18
A_24_P530900	A_24_P530900	0.0297	-2.48
A_24_P541482	A_24_P541482	0.0120	-2.20
A_24_P541483	A_24_P541483	0.0032	-1.54
A_24_P565898	A_24_P565898	0.0408	-1.56
A_24_P585660	A_24_P585660	0.0364	-2.80
A_24_P596251	A_24_P596251	0.0219	-3.18
A_24_P607195	A_24_P607195	0.0006	1.79
A_24_P625898	A_24_P625898	0.0114	-1.66
A_24_P67378	A_24_P67378	0.0300	-2.56
A_24_P701814	A_24_P701814	0.0222	-1.96
A_24_P7040	A_24_P7040	0.0371	1.54
A_24_P713893	A_24_P713893	0.0027	-1.92
A_24_P739582	A_24_P739582	0.0131	-1.73
A_24_P75708	A_24_P75708	0.0233	-2.41
A_24_P7820	A_24_P7820	0.0049	-2.16
A_24_P786713	A_24_P786713	0.0207	-2.29
A_24_P793228	A_24_P793228	0.0137	-1.54
A_24_P832737	A_24_P832737	0.0103	-1.52
A_24_P834646	A_24_P834646	0.0468	-1.58
A_24_P84482	A_24_P84482	0.0265	-1.63
A_24_P872359	A_24_P872359	0.0025	-1.61
A_24_P882666	A_24_P882666	0.0013	-2.91
A_24_P891265	A_24_P891265	0.0101	-1.52
A_24_P900721	A_24_P900721	0.0186	-1.60
A_24_P915675	A_24_P915675	0.0048	-2.13
A_24_P92973	A_24_P92973	0.0020	-1.73
A_24_P942151	A_24_P942151	0.0350	-1.84
A_32_P112401	A_32_P112401	0.0463	-1.80
A_32_P127412	A_32_P127412	0.0280	-2.03

A_32_P148047	A_32_P148047	0.0384	1.66
A_32_P152696	A_32_P152696	0.0207	1.71
A_32_P167723	A_32_P167723	0.0006	-1.58
A_32_P169383	A_32_P169383	0.0056	1.56
A_32_P171043	A_32_P171043	0.0314	-1.88
A_32_P192354	A_32_P192354	0.0053	-1.57
A_32_P194182	A_32_P194182	0.0180	2.21
A_32_P20717	A_32_P20717	0.0141	-1.78
A_32_P23187	A_32_P23187	0.0236	-2.75
A_32_P233769	A_32_P233769	0.0122	-1.91
A_32_P36835	A_32_P36835	0.0087	1.57
A_32_P41235	A_32_P41235	0.0210	-1.58
A_32_P51313	A_32_P51313	0.0158	-1.68
A_32_P55414	A_32_P55414	0.0256	1.88
A_32_P55438	A_32_P55438	0.0144	1.93
A_32_P58029	A_32_P58029	0.0422	1.90
A_32_P64025	A_32_P64025	0.0244	1.58
A_32_P64928	A_32_P64928	0.0278	-1.59
A_32_P69987	A_32_P69987	0.0165	-1.81
A_32_P725839	A_32_P725839	0.0096	-1.57
A_32_P79515	A_32_P79515	0.0490	1.55
A_32_P82119	A_32_P82119	0.0145	-1.84
A_23_P500956	B3GNT2	0.0086	-1.90
A_23_P118946	B4GALT6	0.0260	-2.53
A_24_P228228	B4GALT6	0.0402	-2.28
A_23_P52207	BAMBI	0.0254	-1.76
A_23_P127495	BBOX1	0.0193	21.04
A_24_P145103	BC001335	0.0420	1.76
A_23_P310295	BC002811	0.0151	-1.52
A_32_P114447	BC013799	0.0107	-1.53
A_32_P159574	BC028022	0.0072	1.50
A_32_P178635	BC033590	0.0094	2.18
A_24_P576219	BC035091	0.0048	2.36
A_24_P490857	BC037535	0.0128	-1.50
A_32_P182186	BC041955	0.0375	2.47
A_23_P300484	BC061909	0.0325	2.00
A_23_P359174	BC069659	0.0083	2.85
A_32_P7516	BC071773	0.0120	2.37
A_32_P91107	BC089156	0.0136	1.62
A_24_P161725	BC089388	0.0174	2.18
A_32_P166760	BE074871	0.0113	-1.53
A_32_P103474	BE144057	0.0170	-1.93

A_32_P54987	BE881987	0.0406	-2.08
A_23_P76480	BF213738	0.0004	-3.26
A_32_P142827	BF370042	0.0279	1.85
A_24_P118341	BF869497	0.0368	-2.24
A_32_P171903	BG209623	0.0349	-2.15
A_32_P112452	BI026064	0.0285	-1.74
A_23_P404667	BIK	0.0078	-1.91
A_23_P165333	BIN1	0.0125	1.73
A_24_P156993	BIN1	0.0154	1.67
A_23_P98350	BIRC3	0.0006	-1.55
A_23_P310911	BLMH	0.0043	1.63
A_23_P141606	BLMH	0.0122	1.69
A_24_P119259	BLZF1	0.0051	-1.79
A_32_P76865	BM979049	0.0390	-1.91
A_24_P303989	BMI1	0.0283	-1.94
A_23_P143331	BMP2	0.0047	-3.23
A_23_P19624	BMP6	0.0464	-2.85
A_24_P118531	BP290435	0.0257	-1.89
A_32_P6323	BQ014459	0.0091	-1.50
A_32_P128960	BQ014494	0.0293	-1.56
A_32_P207789	BQ017638	0.0216	1.81
A_24_P180849	BSPRY	0.0199	1.56
A_23_P39465	BST2	0.0435	1.94
A_32_P57013	BU540282	0.0291	1.72
A_32_P85495	BU742669	0.0212	-1.63
A_32_P23517	BU753102	0.0265	-1.50
A_32_P124833	BX100088	0.0247	1.52
A_32_P228886	BX115350	0.0458	1.81
A_24_P924389	BZW1	0.0258	-2.18
A_24_P882914	C10orf46	0.0129	-1.81
A_23_P353726	C10orf46	0.0232	-1.73
A_23_P430902	C10orf83	0.0417	1.65
A_23_P1722	C11orf52	0.0171	1.67
A_32_P185701	C11orf67	0.0368	1.81
A_24_P398781	C14orf179	0.0196	1.51
A_23_P50674	C19orf36	0.0009	-1.78
A_23_P103433	C1orf102	0.0312	1.73
A_23_P256142	C1orf108	0.0006	-2.51
A_23_P256148	C1orf108	0.0014	-1.83
A_24_P233786	C1orf24	0.0269	2.76
A_23_P12147	C1orf74	0.0391	1.64
A_24_P323941	C20orf106	0.0003	-1.62

A_23_P303548	C20orf112	0.0192	-1.94
A_32_P32179	C20orf117	0.0269	1.57
A_24_P65941	C21orf96	0.0247	2.26
A_24_P6083	C22orf16	0.0242	1.94
A_23_P415511	C2orf15	0.0366	2.23
A_23_P108437	C2orf31	0.0076	1.55
A_23_P101407	C3	0.0038	-1.65
A_23_P319874	C3orf23	0.0330	-1.84
A_23_P144134	C3orf58	0.0050	-1.59
A_23_P326963	C3orf59	0.0108	-1.95
A_24_P363023	C4orf16	0.0174	-1.53
A_23_P501831	C5orf4	0.0263	-1.99
A_32_P95757	C6orf128	0.0436	1.55
A_32_P208136	C6orf167	0.0292	-1.87
A_24_P3804	C6orf26	0.0385	1.79
A_23_P257057	C8orf55	0.0368	1.58
A_24_P100517	C9orf140	0.0136	1.75
A_23_P422831	C9orf61	0.0457	3.31
A_23_P405873	C9orf72	0.0453	-1.73
A_23_P8913	CA2	0.0227	2.33
A_32_P205722	CA311162	0.0065	-1.72
A_32_P213103	CA414006	0.0479	2.25
A_23_P43197	CALB1	0.0153	3.92
A_23_P42882	CAMK2B	0.0422	1.83
A_23_P250347	CAMK4	0.0208	-2.21
A_23_P431933	CAMKK1	0.0009	1.61
A_32_P224666	CAPZA2	0.0264	-1.90
A_32_P34926	CARD14	0.0097	2.08
A_23_P207879	CARD14	0.0170	2.07
A_23_P320070	CARD14	0.0295	1.59
A_23_P38085	CARHSP1	0.0163	1.85
A_32_P162716	CB529149	0.0112	-1.77
A_32_P136800	CB984746	0.0394	1.52
A_24_P170593	CBWD3	0.0174	-1.84
A_24_P370372	CBX6	0.0428	1.87
A_23_P55544	CCBE1	0.0076	-2.09
A_24_P369232	CCDC3	0.0206	2.51
A_23_P164958	CCDC8	0.0490	1.67
A_23_P48414	CCNA1	0.0224	-1.67
A_32_P72822	CCNB2	0.0342	3.13
A_24_P80532	CCNG2	0.0297	-2.38
A_23_P331928	CD109	0.0004	-1.88

A_23_P393598	CD276	0.0264	-1.60
A_23_P34597	CDA	0.0337	-1.53
A_23_P66777	CDC27	0.0270	-1.59
A_23_P167767	CDC42SE2	0.0457	-1.77
A_23_P84118	CDH18	0.0134	1.56
A_23_P371865	CDYL2	0.0012	-1.78
A_23_P434118	CEACAM1	0.0304	-1.82
A_24_P382319	CEACAM1	0.0386	-2.93
A_24_P224727	CEBPA	0.0056	2.37
A_23_P375494	CEBPA	0.0111	2.42
A_23_P201570	CELSR2	0.0055	1.54
A_24_P182620	CELSR2	0.0087	1.72
A_23_P92093	CELSR3	0.0258	1.57
A_23_P49816	CENTA2	0.0058	3.00
A_24_P373562	CENTA2	0.0067	3.46
A_24_P36944	CEP170	0.0036	-1.70
A_23_P23151	CEP170	0.0162	-1.53
A_23_P348857	CEP170	0.0217	-1.62
A_32_P148710	CFL1	0.0427	-2.17
A_23_P35820	CFL1	0.0466	-2.11
A_23_P102743	CGI-09	0.0035	-2.49
A_24_P85317	CHD2	0.0351	-2.28
A_23_P116123	CHEK1	0.0311	-2.09
A_23_P382584	CHGB	0.0251	-2.46
A_23_P6321	CLDN5	0.0425	-5.58
A_24_P201702	CLEC2B	0.0002	-2.85
A_23_P53724	CLSTN3	0.0074	2.66
A_23_P252671	CLTB	0.0092	1.69
A_23_P215913	CLU	0.0115	1.70
A_24_P291680	CNOT6L	0.0425	-1.98
A_23_P211212	COL18A1	0.0156	1.66
A_23_P158096	COL27A1	0.0498	2.05
A_23_P55749	COL5A3	0.0059	2.43
A_23_P69030	COL8A1	0.0379	1.85
A_24_P920521	COMMD6	0.0446	-1.55
A_23_P144257	COPS8	0.0022	-1.79
A_24_P218151	CPA4	0.0360	3.26
A_23_P19754	CPA4	0.0380	3.06
A_24_P4170	CPA6	0.0045	-1.66
A_23_P67198	CPAMD8	0.0217	1.98
A_23_P207837	CPD	0.0232	-1.62
A_32_P122925	CR594732	0.0391	-1.56

A_32_P131633	CR598849	0.0204	-1.50
A_23_P398372	CR602592	0.0240	2.14
A_32_P27991	CR610181	0.0431	2.08
A_23_P208293	CR611629	0.0042	1.51
A_23_P115064	CRABP2	0.0220	2.44
A_23_P394986	CREG2	0.0018	-2.14
A_24_P278393	CREM	0.0297	-1.52
A_23_P51105	CRIM1	0.0324	-1.68
A_23_P74359	CSRP1	0.0428	-1.55
A_24_P225448	CSRP1	0.0477	-1.77
A_23_P146946	CST6	0.0151	5.48
A_24_P333326	CTAGE5	0.0343	-2.47
A_24_P287974	CUEDC1	0.0287	-2.00
A_24_P281908	CUTL2	0.0004	-2.55
A_32_P29632	CXADR	0.0014	2.73
A_23_P57268	CXADR	0.0077	2.40
A_24_P374943	CXADR	0.0251	1.95
A_24_P183150	CXCL3	0.0125	-3.23
A_24_P36745	CXorf38	0.0392	-1.87
A_23_P339582	CXorf38	0.0490	-1.95
A_23_P30995	CYB5R4	0.0085	-2.44
A_23_P103486	CYP2J2	0.0287	1.74
A_24_P391230	CYYR1	0.0304	-1.55
A_24_P940499	D80006	0.0282	-2.57
A_32_P158708	DB380247	0.0149	-1.62
A_23_P26439	DBNDD1	0.0058	2.05
A_23_P28772	DBNDD2	0.0241	1.94
A_23_P369994	DCAMKL1	0.0001	-2.41
A_24_P51061	DCBLD2	0.0330	-1.81
A_23_P64873	DCN	0.0087	-7.35
A_23_P313512	DCP1B	0.0042	-1.51
A_23_P216278	DDEF1	0.0061	-1.67
A_23_P44831	DDEF1	0.0117	-1.60
A_23_P71526	DDEF1	0.0362	-1.82
A_23_P211355	DGCR8	0.0390	1.53
A_23_P21644	DHRS8	0.0167	-1.87
A_23_P93818	DKFZP434A0131	0.0236	1.90
A_24_P171043	DKFZP547L112	0.0494	3.68
A_32_P198731	DKFZP761M1511	0.0196	2.10
A_24_P29686	DKFZp761I2123	0.0020	1.79
A_23_P413923	DMRTA1	0.0440	1.52
A_23_P327361	DMXL2	0.0194	-1.62

A_23_P206140	DNAJA4	0.0334	1.62
A_24_P63827	DNAJB6	0.0410	-1.50
A_23_P127220	DNAJC12	0.0261	2.00
A_23_P252052	DOC1	0.0117	2.29
A_23_P43988	DPYD	0.0233	-1.64
A_24_P38347	DPYSL2	0.0033	-1.80
A_23_P350824	DPYSL2	0.0346	-1.69
A_23_P38696	DSC1	0.0214	3.91
A_23_P109320	DSCR6	0.0173	2.10
A_23_P7896	DUSP22	0.0214	-1.67
A_23_P107750	EDG8	0.0037	2.14
A_23_P107744	EDG8	0.0043	2.60
A_23_P23443	EFHD2	0.0036	-3.17
A_23_P428139	EFNB2	0.0301	-1.54
A_23_P48561	EFS	0.0141	2.73
A_24_P754817	EFTUD1	0.0075	-2.41
A_23_P23171	EIF2C4	0.0040	-2.39
A_24_P943113	EIF4E3	0.0371	-1.55
A_24_P383850	EIF4G3	0.0475	-1.83
A_23_P94571	ELAVL2	0.0228	-1.63
A_23_P104188	ELF3	0.0279	2.47
A_23_P213424	ENC1	0.0198	1.67
A_23_P94338	ENPP2	0.0023	-3.78
A_32_P107746	ENSA	0.0451	1.89
A_24_P168574	ENST00000217126	0.0000	2.19
A_23_P117912	ENST00000220507	0.0003	4.72
A_24_P158718	ENST00000227451	0.0188	1.66
A_24_P66398	ENST00000239730	0.0027	-1.56
A_24_P860797	ENST00000244221	0.0197	1.61
A_24_P366082	ENST00000252134	0.0435	1.52
A_23_P410859	ENST00000252744	0.0176	-1.51
A_23_P89062	ENST00000254109	0.0478	1.73
A_23_P155185	ENST00000256031	0.0253	-1.50
A_24_P102080	ENST00000262646	0.0237	-1.77
A_23_P200043	ENST00000263739	0.0253	1.65
A_24_P930926	ENST00000287322	0.0425	-1.58
A_32_P168342	ENST00000299289	0.0268	1.85
A_24_P220984	ENST00000308269	0.0233	-2.01
A_24_P263803	ENST00000308819	0.0081	-1.51
A_24_P280897	ENST00000310822	0.0353	-2.00
A_23_P400794	ENST00000311630	0.0244	-1.58
A_24_P15586	ENST00000313516	0.0475	-1.98

A_24_P66932	ENST00000313594	0.0253	-1.68
A_24_P170095	ENST00000316131	0.0479	1.78
A_24_P341517	ENST00000317656	0.0337	-1.94
A_24_P349590	ENST00000318251	0.0353	-2.33
A_24_P323421	ENST00000320831	0.0136	-2.25
A_24_P44453	ENST00000322839	0.0417	1.70
A_24_P345290	ENST00000327299	0.0072	-1.56
A_32_P117127	ENST00000330336	0.0148	1.59
A_32_P65473	ENST00000330640	0.0137	1.60
A_32_P20573	ENST00000334464	0.0460	-1.83
A_24_P101541	ENST00000339991	0.0098	-1.99
A_24_P910262	ENST00000354586	0.0081	-1.99
A_23_P394246	ENST00000356987	0.0179	2.38
A_24_P923629	ENST00000357137	0.0121	-1.82
A_23_P208870	ENST00000358335	0.0169	1.76
A_24_P359267	ENST00000358335	0.0182	1.70
A_32_P64461	ENST00000360548	0.0441	-1.50
A_23_P409626	ENST00000360796	0.0357	-1.83
A_23_P406928	ENST00000361567	0.0178	1.70
A_24_P702813	ENST00000367590	0.0018	-1.69
A_32_P197870	ENST00000368847	0.0440	2.18
A_24_P687594	ENST00000369308	0.0396	-2.19
A_24_P673968	ENST00000371276	0.0278	1.59
A_32_P59302	ENST00000372583	0.0266	2.21
A_24_P123658	ENST00000372871	0.0429	1.67
A_24_P396489	ENST00000375590	0.0065	-1.65
A_24_P673786	ENST00000376573	0.0066	-1.84
A_24_P649624	ENST00000377093	0.0063	-1.65
A_23_P324011	ENST00000377538	0.0432	1.52
A_32_P140656	ENST00000381298	0.0008	-1.50
A_24_P269624	ENST00000381854	0.0092	-1.54
A_24_P358146	ENST00000382990	0.0044	-1.78
A_23_P93739	EPDR1	0.0016	-1.52
A_24_P166613	EPDR1	0.0400	-4.29
A_23_P200067	EPHB2	0.0132	1.75
A_23_P8834	EPHX2	0.0323	2.29
A_23_P136347	EPS8	0.0047	-1.69
A_23_P333227	ERGIC1	0.0318	-1.62
A_24_P407311	ERO1L	0.0115	-2.55
A_23_P106145	ERO1L	0.0123	-2.11
A_23_P9836	ETV5	0.0418	-1.68
A_24_P256830	EXOC5	0.0123	-1.58

A_24_P224219	EXOC5	0.0173	-1.52
A_24_P392265	EXOC5	0.0174	-1.51
A_23_P14464	EXOC5	0.0420	-1.54
A_32_P58280	EXOC6	0.0461	-1.73
A_23_P500421	EYA2	0.0105	2.20
A_23_P64404	FADS3	0.0183	1.62
A_23_P415061	FAM104B	0.0413	-1.79
A_23_P320878	FAM119B	0.0425	-1.75
A_23_P157365	FAM3C	0.0377	-2.00
A_24_P370042	FAM59A	0.0108	-1.76
A_23_P343963	FAM83F	0.0186	-1.68
A_23_P356101	FBXO11	0.0077	-1.52
A_24_P357536	FBXO11	0.0216	-1.72
A_24_P106522	FBXO9	0.0272	1.63
A_23_P212800	FGF5	0.0168	-1.69
A_23_P105803	FGF9	0.0235	-1.57
A_23_P79622	FKBP7	0.0259	-1.71
A_23_P38894	FLJ11286	0.0246	1.56
A_23_P25060	FLJ13769	0.0281	1.93
A_32_P148745	FLJ14712	0.0279	2.72
A_23_P147109	FLJ20245	0.0118	-2.55
A_23_P147106	FLJ20245	0.0229	-2.16
A_23_P21485	FLJ20701	0.0202	-1.91
A_23_P152583	FLJ21865	0.0251	1.51
A_24_P838448	FLJ22536	0.0107	1.81
A_23_P83835	FLJ23356	0.0128	-1.59
A_23_P149798	FLJ23556	0.0283	1.73
A_23_P350005	FLJ25801	0.0088	-1.67
A_24_P677642	FLJ31813	0.0304	-1.55
A_23_P370569	FLJ32549	0.0123	-1.54
A_24_P59471	FLJ36840	0.0413	2.49
A_23_P253677	FLJ38482	0.0188	-1.60
A_24_P20524	FLJ38482	0.0212	-1.50
A_23_P163467	FLJ43339	0.0325	2.00
A_23_P55319	FLOT2	0.0054	1.79
A_24_P40417	FMR1	0.0140	-1.98
A_23_P25503	FNDC3A	0.0484	-1.74
A_23_P38795	FPR1	0.0026	-2.71
A_24_P374741	FRMD4A	0.0085	2.08
A_23_P22352	FRMD4A	0.0259	2.34
A_23_P25888	FRMD6	0.0336	-1.64
A_23_P353125	FSIP1	0.0233	-1.92

A_23_P141362	FZD2	0.0168	1.65
A_23_P397999	FZD5	0.0014	1.54
A_24_P380132	G3BP2	0.0105	-1.54
A_23_P19102	GALNT10	0.0002	3.23
A_23_P7706	GALNT10	0.0356	1.62
A_24_P910923	GALNT10	0.0356	1.70
A_23_P304450	GATA6	0.0274	-1.66
A_23_P28485	GCA	0.0113	1.83
A_23_P9232	GCNT1	0.0004	-3.15
A_24_P393571	GDA	0.0027	-9.77
A_23_P26511	GDPD3	0.0038	1.75
A_23_P134910	GGH	0.0269	-1.73
A_23_P93591	GJA1	0.0062	-2.09
A_23_P96556	GK	0.0188	-1.62
A_23_P337141	GLI4	0.0417	1.56
A_23_P316612	GLIS1	0.0067	-3.06
A_24_P277657	GMPR	0.0265	1.84
A_24_P353912	GNAI3	0.0104	-1.78
A_23_P69918	GNL3L	0.0325	1.82
A_23_P104237	GPAM	0.0074	-2.22
A_23_P119812	GPD2	0.0037	-1.70
A_32_P220152	GPIAP1	0.0213	-1.76
A_23_P154150	GPR1	0.0120	-1.59
A_24_P942945	GPR126	0.0200	-1.51
A_23_P15692	GPR172B	0.0257	1.70
A_23_P253692	GPR64	0.0067	-7.48
A_23_P1056	GPR89A	0.0187	-1.59
A_24_P887553	GPR89A	0.0253	-1.86
A_23_P112103	GSDMDC1	0.0415	2.01
A_23_P94118	GTF2E2	0.0239	-1.52
A_23_P104617	GYLTL1B	0.0014	1.77
A_24_P353964	HADHA	0.0402	-1.68
A_32_P25253	HBLD2	0.0359	-1.59
A_24_P387609	HBLD2	0.0401	-1.64
A_24_P75543	HBLD2	0.0412	-1.81
A_32_P85500	HCP5	0.0036	1.70
A_24_P205137	HDAC6	0.0085	-1.54
A_23_P22526	HEPH	0.0064	3.14
A_23_P304716	HES2	0.0229	1.54
A_23_P300174	HEXDC	0.0055	1.53
A_24_P9321	HIST1H3I	0.0215	-1.57
A_24_P85099	HMGA2	0.0183	-1.82

A_23_P256107	HPSE	0.0294	-1.73
A_23_P116414	HRASLS3	0.0085	1.69
A_23_P200109	HS2ST1	0.0351	-1.60
A_23_P118158	HS3ST2	0.0110	-3.72
A_23_P14986	HSD11B2	0.0440	2.04
A_23_P118065	HSD17B2	0.0282	-12.23
A_23_P204967	HSMPP8	0.0116	5.61
A_24_P287403	HSMPP8	0.0145	5.60
A_23_P500381	HTR7	0.0334	-1.62
A_23_P156977	HUS1	0.0400	-1.53
A_23_P344988	ICK	0.0430	-1.73
A_23_P143143	ID2	0.0490	-2.08
A_23_P137381	ID3	0.0024	1.82
A_23_P153745	IFI30	0.0048	1.54
A_23_P23074	IFI44	0.0320	2.63
A_23_P201459	IFI6	0.0475	3.48
A_23_P72737	IFITM1	0.0001	4.44
A_23_P119943	IGFBP2	0.0325	1.84
A_23_P139912	IGFBP6	0.0004	-1.59
A_23_P138680	IL15RA	0.0043	2.07
A_23_P329152	ILF3	0.0042	1.58
A_24_P101200	IMPAD1	0.0057	-2.29
A_23_P383532	IMPAD1	0.0371	-1.73
A_24_P240732	IMPAD1	0.0461	-1.98
A_32_P120638	INTS4	0.0184	-1.67
A_23_P19852	IQCE	0.0356	2.01
A_23_P162300	IRAK3	0.0033	-2.75
A_24_P243329	ITGA2	0.0390	-2.14
A_23_P36562	ITGA5	0.0242	-1.84
A_23_P381992	ITGAV	0.0028	-1.99
A_24_P598836	ITGB1	0.0062	-1.70
A_23_P104199	ITGB1	0.0114	-2.12
A_23_P104193	ITGB1	0.0120	-1.62
A_23_P329573	ITGB2	0.0059	-3.00
A_23_P65918	ITPKA	0.0080	-1.97
A_23_P54055	JUB	0.0311	1.54
A_24_P924484	K03200	0.0002	-2.29
A_23_P86100	KARCA1	0.0359	2.12
A_32_P75284	KATNAL1	0.0397	-1.53
A_24_P31627	KCNB1	0.0306	-3.01
A_23_P210581	KCNG1	0.0306	1.95
A_32_P181222	KCNMA1	0.0055	-2.61

A_32_P160693	KCTD2	0.0240	-1.85
A_24_P82135	KIAA0256	0.0041	-2.56
A_32_P355396	KIAA0329	0.0227	1.81
A_23_P406025	KIAA0367	0.0006	-5.09
A_24_P320727	KIAA1324L	0.0218	-1.89
A_24_P350124	KIAA1618	0.0469	1.62
A_24_P130959	KIAA1804	0.0010	-1.66
A_24_P130952	KIAA1804	0.0224	-1.52
A_23_P43597	KIF24	0.0287	2.19
A_24_P93948	KIF5A	0.0006	2.20
A_24_P17719	KLHL5	0.0179	-1.85
A_23_P121527	KLHL5	0.0194	-1.90
A_24_P17710	KLHL5	0.0241	-2.23
A_23_P101505	KLK11	0.0058	3.07
A_23_P4856	KLK15	0.0033	1.71
A_23_P369343	KLK8	0.0270	1.86
A_24_P102539	KRIT1	0.0242	-1.62
A_23_P101054	KRT34	0.0240	-2.13
A_23_P116942	LAG3	0.0333	1.92
A_32_P81334	LARP4	0.0189	-1.56
A_23_P69179	LEPREL1	0.0061	-1.55
A_23_P15357	LGALS3BP	0.0092	1.66
A_24_P940979	LGALS8	0.0110	-1.64
A_32_P452655	LGALS9	0.0228	2.69
A_24_P397386	LIFR	0.0376	-1.68
A_23_P139277	LIN7C	0.0046	-1.87
A_23_P327698	LMBRD2	0.0223	-1.55
A_32_P113935	LNX2	0.0307	-1.53
A_24_P841622	LOC132241	0.0395	1.58
A_24_P273647	LOC146439	0.0401	2.88
A_23_P55846	LOC147804	0.0386	-2.29
A_24_P57170	LOC148137	0.0054	1.62
A_24_P203964	LOC153561	0.0419	1.76
A_24_P358245	LOC158381	0.0156	-1.78
A_32_P72553	LOC162073	0.0434	2.20
A_23_P392126	LOC201229	0.0403	1.96
A_32_P154911	LOC222171	0.0486	-2.58
A_32_P122323	LOC285749	0.0021	2.19
A_32_P151366	LOC285813	0.0253	1.66
A_32_P13370	LOC286161	0.0331	1.52
A_24_P135579	LOC344382	0.0450	-1.91
A_23_P138885	LOC387758	0.0142	1.93

A_32_P76992	LOC388114	0.0032	1.64
A_24_P734953	LOC388610	0.0238	1.61
A_24_P75979	LOC402643	0.0216	-2.45
A_24_P93703	LOC440104	0.0281	1.66
A_32_P170749	LOC442578	0.0390	1.75
A_23_P149441	LOC51152	0.0111	1.88
A_24_P825942	LOC541471	0.0332	-2.14
A_23_P349463	LOC63928	0.0039	3.54
A_24_P915710	LOC63929	0.0053	-2.12
A_24_P272352	LOC649125	0.0259	-1.55
A_32_P146898	LOC653188	0.0208	1.89
A_23_P168592	LOC90693	0.0022	-1.59
A_23_P168587	LOC90693	0.0420	-1.62
A_23_P111995	LOXL2	0.0078	-1.65
A_23_P151791	LTB4R	0.0002	1.99
A_23_P134764	LY6D	0.0380	1.61
A_24_P48495	LYPD3	0.0131	1.87
A_23_P39265	LYPD3	0.0387	1.94
A_23_P397376	MAF	0.0235	4.51
A_23_P99996	MAGEL2	0.0467	-2.26
A_23_P103601	MAN1C1	0.0276	1.51
A_24_P187921	MANEA	0.0359	-1.75
A_24_P123385	MAP1B	0.0216	-1.98
A_23_P102192	MAP4K4	0.0044	-1.69
A_23_P321511	03-Mar	0.0070	-2.77
A_23_P201988	MASTL	0.0367	-1.70
A_23_P81408	MAT2B	0.0199	-1.69
A_23_P71328	MATN2	0.0262	1.90
A_23_P142537	MBD5	0.0256	-2.34
A_23_P14946	MBTPS1	0.0008	-1.73
A_23_P14948	MBTPS1	0.0029	-1.84
A_24_P378368	MBTPS1	0.0042	-2.11
A_23_P329271	MC1R	0.0391	1.70
A_24_P103060	MCFD2	0.0144	-2.33
A_24_P319635	MCL1	0.0250	-1.55
A_32_P120567	MDFIC	0.0155	-1.73
A_24_P926960	MEGF6	0.0109	2.02
A_23_P77223	MESP1	0.0122	1.93
A_24_P339201	METRN	0.0011	2.19
A_23_P208069	METTL4	0.0230	-1.58
A_24_P202139	METTL9	0.0085	-1.65
A_23_P386320	MFI2	0.0009	-2.76

A_23_P103104	MFNG	0.0031	2.09
A_24_P224926	MFNG	0.0074	2.11
A_23_P16806	MGC10701	0.0193	1.62
A_23_P143102	MGC10955	0.0409	-2.15
A_24_P104649	MGC15523	0.0471	1.66
A_23_P328621	MGC20470	0.0374	1.78
A_23_P66719	MGC23280	0.0158	1.53
A_23_P256051	MGC70863	0.0289	-1.67
A_23_P403195	MICAL3	0.0341	1.55
A_23_P154400	MLPH	0.0040	-3.40
A_23_P165783	MLPH	0.0096	-3.35
A_23_P165778	MLPH	0.0164	-3.03
A_24_P260101	MME	0.0353	-1.72
A_23_P108922	MOBK1B	0.0164	-1.99
A_24_P333445	MORF4L2	0.0136	-1.65
A_23_P200685	MOSC2	0.0313	1.98
A_24_P126093	MOSPD2	0.0339	-2.07
A_24_P345123	MPZL1	0.0104	-1.64
A_23_P144453	MRFAP1	0.0159	-1.58
A_24_P212389	MSRB3	0.0227	-1.68
A_23_P206998	MTMR4	0.0070	-2.63
A_24_P382119	MTMR4	0.0119	-1.59
A_23_P25515	MTMR6	0.0154	-1.69
A_24_P261052	MTMR9	0.0104	-1.52
A_24_P77870	MUTED	0.0469	-1.83
A_23_P17663	MX1	0.0044	2.56
A_23_P124559	MXD3	0.0265	1.55
A_24_P256764	MYH10	0.0177	-1.80
A_23_P26865	MYH3	0.0373	1.64
A_24_P917123	MYLIP	0.0481	2.52
A_32_P217471	MYO1B	0.0003	-1.66
A_24_P7143	MYO1B	0.0014	-1.69
A_23_P432610	N4BP1	0.0142	1.72
A_23_P58747	N4BP3	0.0434	1.88
A_24_P221770	NAT12	0.0493	-1.56
A_23_P52727	NAV2	0.0467	1.92
A_23_P409168	NBEAL2	0.0099	1.65
A_23_P86424	NCOA4	0.0259	-1.51
A_23_P86421	NCOA4	0.0362	-1.53
A_24_P237661	NCOR1	0.0235	-1.65
A_23_P37205	NDRG2	0.0286	2.11
A_24_P936444	NFE2L2	0.0020	-1.61

A_24_P49731	NFYB	0.0378	-1.64
A_24_P338971	NIPA2	0.0343	-1.75
A_24_P399606	NOTCH3	0.0469	1.60
A_23_P254353	NOXA1	0.0160	2.79
A_23_P140256	NP	0.0012	-2.42
A_24_P252364	NRCAM	0.0189	-1.92
A_23_P59418	NRF1	0.0152	-1.50
A_23_P86390	NRP1	0.0201	-1.75
A_24_P50801	NRP2	0.0404	-1.72
A_23_P209669	NRP2	0.0419	-1.94
A_24_P218056	NUDCD3	0.0162	-1.71
A_32_P117723	NUDT4	0.0228	-1.60
A_24_P200761	NUP43	0.0499	-1.52
A_23_P63128	OBSCN	0.0320	1.68
A_23_P335695	OBSL1	0.0007	2.70
A_24_P220485	OLFML2A	0.0472	2.44
A_24_P11315	OLFML3	0.0342	-2.89
A_32_P24832	OLFML3	0.0462	-1.75
A_23_P74391	OPN3	0.0102	-1.65
A_24_P153511	OSBPL8	0.0383	-1.74
A_23_P81522	OSRF	0.0149	-2.08
A_24_P124875	OTUB2	0.0173	-1.52
A_23_P212974	OTUD4	0.0096	-1.88
A_23_P69637	OTUD4	0.0373	-2.41
A_23_P25069	OVOS2	0.0339	1.79
A_23_P64611	P2RY6	0.0139	2.71
A_24_P152398	P53AIP1	0.0402	1.87
A_23_P51718	PABPC4	0.0051	-1.56
A_23_P337875	PAQR3	0.0184	-2.17
A_23_P343837	PARP11	0.0027	-2.46
A_23_P305060	PBEF1	0.0163	-1.62
A_24_P408772	PBEF1	0.0251	-2.30
A_32_P79396	PBEF1	0.0266	-1.90
A_23_P41128	PCAF	0.0395	-2.45
A_24_P360206	PCDHA11	0.0104	-2.83
A_23_P218942	PCDHAC2	0.0345	-3.45
A_32_P142440	PCSK9	0.0250	-5.99
A_23_P33376	PCTK2	0.0490	-1.80
A_23_P56640	PCYOX1	0.0308	-1.52
A_23_P151198	PDAP1	0.0157	2.24
A_23_P123256	PDAP1	0.0366	1.72
A_23_P68730	PDXK	0.0037	1.53

A_23_P7402	PDZD2	0.0143	-2.61
A_24_P79403	PF4	0.0278	-1.81
A_23_P113311	PH-4	0.0070	1.84
A_23_P113317	PH-4	0.0083	1.76
A_23_P217399	PIGA	0.0449	-2.35
A_32_P167148	PIGW	0.0465	-1.63
A_23_P84189	PITPNC1	0.0459	-2.00
A_23_P58642	PITX1	0.0496	1.71
A_24_P133288	PKP2	0.0262	-1.63
A_23_P162466	PKP2	0.0361	-1.74
A_24_P379616	PLB1	0.0415	2.03
A_23_P205801	PLDN	0.0011	-2.24
A_23_P205808	PLDN	0.0019	-1.92
A_24_P27373	PLDN	0.0043	-1.74
A_23_P406889	PLDN	0.0116	-2.01
A_24_P13041	PLEKHK1	0.0074	2.54
A_23_P100711	PMP22	0.0015	-2.67
A_23_P215060	PODXL	0.0360	2.11
A_23_P159764	POF1B	0.0399	2.04
A_23_P215431	POM121	0.0044	1.51
A_23_P11071	PORCN	0.0447	-1.54
A_23_P59138	POU5F1	0.0017	1.68
A_23_P425704	PPARBP	0.0185	1.52
A_23_P150807	PPFIBP2	0.0003	2.13
A_23_P409623	PPFIBP2	0.0023	1.89
A_23_P409553	PPM1A	0.0019	-2.20
A_24_P183375	PPP2CA	0.0308	-1.59
A_23_P213620	PPP2R2B	0.0069	3.98
A_24_P3415	PPP2R2C	0.0027	-2.97
A_23_P108592	PPP3R1	0.0001	-2.32
A_23_P331908	PRDM11	0.0441	1.74
A_23_P314760	PRKAG2	0.0050	-1.66
A_23_P44366	PRKAG2	0.0299	-1.52
A_24_P356592	PRKAR1A	0.0464	-1.90
A_23_P106016	PRKD1	0.0102	-1.89
A_23_P139740	PRMT8	0.0017	-2.46
A_24_P119141	PROS1	0.0017	2.14
A_23_P73114	PROS1	0.0047	2.12
A_23_P60537	PRPF4	0.0012	-1.57
A_23_P129602	PRSS21	0.0148	-2.45
A_24_P937405	PRSS23	0.0284	1.60
A_24_P263623	PTGES3	0.0345	-1.78

A_23_P216966	PTGS1	0.0387	-1.63
A_24_P178273	PTK9	0.0016	-1.61
A_23_P99920	PTPLAD1	0.0499	-1.69
A_23_P338890	PTPN1	0.0271	-1.75
A_23_P101642	PTPRH	0.0152	-3.18
A_23_P141376	PTRF	0.0293	1.58
A_23_P80759	PVRL3	0.0075	-1.72
A_23_P80763	PVRL3	0.0197	-1.58
A_24_P941322	QKI	0.0020	-2.03
A_24_P96234	QTRT1	0.0262	1.68
A_23_P165879	RAB10	0.0223	-1.70
A_24_P193295	RAB15	0.0109	1.97
A_32_P179258	RAB22A	0.0119	-1.64
A_23_P115091	RAB25	0.0461	1.68
A_32_P7204	RAB28	0.0268	-2.22
A_23_P125147	RAB28	0.0340	-2.02
A_23_P6447	RABL4	0.0064	1.84
A_23_P40952	RAF1	0.0021	-1.53
A_23_P92727	RAI14	0.0098	-2.28
A_23_P50946	RAMP1	0.0179	1.78
A_23_P2661	RAP1B	0.0158	-1.51
A_23_P1962	RARRES3	0.0129	1.77
A_23_P119353	RASIP1	0.0415	1.78
A_24_P407224	RASSF8	0.0001	-1.72
A_24_P913561	RASSF8	0.0017	-2.21
A_23_P116712	RASSF8	0.0035	-1.75
A_24_P404807	RBM41	0.0055	-1.52
A_23_P103099	RBM9	0.0076	-1.89
A_23_P257649	RBP1	0.0066	1.56
A_23_P257457	RDHE2	0.0246	-3.83
A_23_P141447	RDM1	0.0297	2.08
A_23_P25215	RECQL	0.0338	-1.64
A_23_P19182	REEP2	0.0074	1.65
A_23_P330209	RFFL	0.0028	-1.57
A_23_P320578	RGS16	0.0393	1.64
A_23_P20427	RHOBTB2	0.0431	2.09
A_23_P424561	RHOV	0.0002	4.65
A_23_P315386	RHPN1	0.0049	2.43
A_23_P161686	RICS	0.0160	1.59
A_24_P34476	RIF1	0.0340	-1.53
A_24_P360269	RNASET2	0.0065	1.51
A_32_P131367	RNF13	0.0498	-1.65

A_23_P416112	RNF168	0.0116	-1.55
A_23_P130429	ROCK1	0.0050	-1.79
A_23_P70278	ROS1	0.0236	-2.58
A_23_P128574	RP11-301I17.1	0.0011	-3.40
A_24_P602871	RP5-875H10.1	0.0326	3.16
A_23_P108932	RPL23AP13	0.0422	1.77
A_24_P307025	RPL23AP7	0.0377	-1.68
A_23_P335920	RPS6KA2	0.0257	-1.82
A_24_P237601	RPS6KA5	0.0001	-2.44
A_24_P304636	RPS6KA5	0.0014	-2.06
A_24_P590560	RRN3	0.0087	-1.86
A_23_P206877	RRN3	0.0257	-1.59
A_24_P296347	RRN3	0.0347	-1.59
A_32_P161762	RUNX2	0.0052	-2.29
A_23_P23048	S100A9	0.0001	-2.31
A_23_P48585	SALL2	0.0258	1.74
A_23_P29005	SAMSN1	0.0322	-6.58
A_32_P37592	SCARNA17	0.0053	2.29
A_24_P385134	SCD5	0.0002	1.64
A_23_P212475	SCOTIN	0.0003	-1.97
A_24_P394246	SCOTIN	0.0211	-2.28
A_23_P93722	SDK1	0.0034	2.07
A_32_P29118	SEMA3D	0.0126	2.20
A_23_P95165	SEMA4B	0.0011	-1.55
A_23_P121374	SEMA5B	0.0023	-1.68
A_23_P119015	SERPINB13	0.0259	1.61
A_24_P250614	SF3A1	0.0473	-1.64
A_23_P254254	SGSH	0.0104	1.86
A_23_P61688	SLC12A7	0.0293	1.74
A_24_P179467	SLC1A6	0.0326	2.18
A_24_P103004	SLC20A1	0.0314	-1.77
A_23_P125078	SLC26A11	0.0010	2.86
A_23_P140450	SLC27A2	0.0031	3.03
A_23_P41789	SLC27A6	0.0027	5.04
A_23_P22915	SLC30A7	0.0040	-1.62
A_23_P217109	SLC31A2	0.0187	-1.52
A_23_P17695	SLC37A1	0.0435	1.79
A_23_P23575	SLC39A1	0.0009	-1.74
A_23_P342641	SLC44A5	0.0011	-1.53
A_24_P109838	SLC6A15	0.0114	-1.95
A_23_P13725	SLC6A15	0.0221	-1.67
A_23_P135990	SLCO2A1	0.0308	-4.22

A_23_P144348	SLIT2	0.0016	5.59
A_24_P942773	SLMAP	0.0372	-1.61
A_32_P109002	SMAD2	0.0027	-1.69
A_24_P150466	SMOC1	0.0343	1.61
A_23_P160881	SMPDL3B	0.0209	1.70
A_24_P937139	SNAI2	0.0173	-2.49
A_23_P23175	SNIP1	0.0042	-2.80
A_24_P936688	SNRK	0.0127	-2.47
A_24_P375421	SNX22	0.0000	-2.26
A_24_P179044	SNX9	0.0167	-1.65
A_24_P216654	SOAT1	0.0032	-1.98
A_23_P63319	SOAT1	0.0139	-2.26
A_24_P48014	SOCS1	0.0103	-1.58
A_23_P5813	SOCS5	0.0215	-1.66
A_23_P121665	SORCS2	0.0482	2.05
A_24_P325520	SORT1	0.0012	1.73
A_23_P209712	SP100	0.0364	1.55
A_23_P146066	SPAG1	0.0246	-2.14
A_32_P219660	SPANXA1	0.0018	-2.28
A_23_P209356	SPAST	0.0063	-1.56
A_24_P941988	SPAST	0.0219	-2.32
A_23_P52219	SPFH1	0.0169	-1.93
A_23_P433990	SPG7	0.0311	-1.71
A_23_P74012	SPRR1A	0.0389	2.48
A_24_P337657	SRF	0.0195	-1.61
A_23_P162449	SRGAP1	0.0399	1.69
A_24_P922261	SRGAP1	0.0409	2.46
A_23_P74269	SRM	0.0057	-2.00
A_23_P96383	SRPX	0.0019	-1.66
A_24_P679409	SRRM1	0.0154	-1.60
A_24_P159916	SSBP4	0.0442	1.53
A_23_P150147	SSH3	0.0095	1.60
A_32_P50431	ST13	0.0104	-1.81
A_24_P268123	ST3GAL3	0.0135	-2.07
A_23_P33856	STAG2	0.0487	-1.74
A_23_P327519	STARD4	0.0375	-1.87
A_23_P158880	STARD5	0.0222	1.52
A_23_P309837	STON2	0.0051	1.50
A_24_P201171	STXBP1	0.0250	-1.85
A_24_P398746	STYX	0.0162	-1.66
A_23_P43164	SULF1	0.0048	-2.34
A_23_P434212	SULT1A1	0.0303	1.53

A_24_P262201	SULT1A4	0.0317	1.62
A_23_P107981	SULT2B1	0.0138	1.84
A_23_P78808	SYMPK	0.0196	1.54
A_23_P46390	SYTL1	0.0349	1.54
A_23_P91081	TACSTD1	0.0012	1.55
A_23_P74663	TAF1A	0.0485	-1.51
A_24_P376441	TAF5L	0.0254	-2.46
A_23_P325887	TBC1D8B	0.0146	-2.56
A_23_P114172	TBC1D8B	0.0168	-1.62
A_23_P142872	TCF7L1	0.0005	1.69
A_32_P208350	TDRD9	0.0023	-3.51
A_24_P483083	TFDP1	0.0258	-1.82
A_23_P17095	TFPI	0.0000	-5.68
A_23_P393620	TFPI2	0.0084	-3.14
A_23_P62021	THBS2	0.0061	-1.54
A_32_P52119	THC2250585	0.0135	1.55
A_24_P921402	THC2263973	0.0479	1.95
A_32_P171921	THC2265989	0.0331	1.52
A_24_P592591	THC2270231	0.0202	-1.88
A_24_P863124	THC2276723	0.0436	-1.98
A_32_P24939	THC2278570	0.0004	-4.23
A_32_P3932	THC2280799	0.0359	1.67
A_24_P649735	THC2281176	0.0138	1.73
A_24_P367421	THC2283170	0.0405	-2.31
A_32_P117730	THC2283727	0.0454	1.64
A_32_P212294	THC2283810	0.0425	-1.77
A_32_P65843	THC2289112	0.0481	1.50
A_24_P494807	THC2294587	0.0385	-1.80
A_32_P231143	THC2296760	0.0488	2.35
A_24_P703462	THC2308747	0.0016	-1.56
A_32_P84707	THC2308802	0.0235	3.03
A_32_P125820	THC2314239	0.0471	1.51
A_32_P139551	THC2316649	0.0119	2.39
A_24_P795106	THC2317099	0.0445	1.89
A_32_P34970	THC2317139	0.0049	-1.74
A_32_P137408	THC2317808	0.0267	-2.14
A_24_P564396	THC2335868	0.0000	-1.84
A_24_P707530	THC2336549	0.0433	-1.89
A_32_P224586	THC2342537	0.0219	2.77
A_32_P148380	THC2343919	0.0454	-1.62
A_32_P167176	THC2343936	0.0326	2.58
A_32_P177477	THC2344152	0.0295	2.12

A_32_P167883	THC2344420	0.0153	2.20
A_32_P190682	THC2347318	0.0464	2.17
A_32_P118481	THC2357608	0.0060	-2.11
A_32_P213002	THC2364440	0.0359	1.71
A_24_P455579	THC2365025	0.0152	2.02
A_32_P221517	THC2365212	0.0001	1.78
A_23_P82488	THC2372503	0.0419	-1.68
A_32_P82671	THC2373077	0.0020	-1.88
A_32_P2050	THC2373975	0.0373	1.58
A_32_P217901	THC2375512	0.0065	-2.46
A_23_P165541	THC2376224	0.0006	-1.67
A_32_P5168	THC2377418	0.0474	-1.86
A_23_P57393	THC2377764	0.0104	1.99
A_23_P149938	THC2378635	0.0109	1.76
A_32_P74932	THC2379429	0.0356	3.12
A_32_P42976	THC2381319	0.0174	2.14
A_32_P203408	THC2391898	0.0045	-2.36
A_32_P233834	THC2392085	0.0404	1.89
A_32_P12820	THC2400533	0.0037	-1.96
A_32_P74477	THC2403217	0.0318	1.73
A_32_P146844	THC2406576	0.0331	2.59
A_32_P121755	THC2406579	0.0173	1.54
A_32_P13337	THC2408277	0.0074	1.88
A_23_P218569	THC2411515	0.0135	-1.81
A_32_P112712	THC2420676	0.0076	-1.73
A_32_P142334	THC2434948	0.0450	2.20
A_32_P158181	THC2437757	0.0033	2.60
A_32_P174572	THC2438039	0.0101	-1.57
A_32_P220523	THC2439328	0.0280	2.25
A_32_P138437	THC2440634	0.0210	2.30
A_32_P111394	THC2441040	0.0084	2.10
A_32_P188193	THC2445517	0.0064	3.98
A_32_P184417	THC2447037	0.0146	-1.87
A_32_P136450	THC2449500	0.0168	-1.53
A_24_P417474	TIA1	0.0133	-1.56
A_23_P167444	TINP1	0.0004	-1.58
A_24_P238333	TINP1	0.0010	-1.51
A_23_P372946	TM4SF19	0.0312	-1.79
A_24_P305933	TMCC3	0.0209	-1.54
A_23_P158277	TMCO4	0.0367	1.73
A_24_P274987	TMEFF1	0.0205	-1.70
A_24_P255082	TMEM110	0.0181	-1.97

A_23_P202964	TMEM123	0.0170	-1.61
A_24_P171058	TMEM64	0.0220	-2.00
A_23_P57089	TMEPAI	0.0436	1.58
A_23_P435477	TMPRSS13	0.0035	3.24
A_23_P203150	TMPRSS13	0.0048	4.73
A_23_P127608	TMPRSS4	0.0065	1.59
A_23_P47735	TNFRSF19L	0.0072	-1.51
A_24_P350576	TNIK	0.0031	-2.12
A_23_P141180	TOM1L2	0.0478	1.52
A_24_P67364	TPM3	0.0241	-2.72
A_24_P161244	TPM3	0.0270	-2.18
A_24_P257224	TPO	0.0330	-2.81
A_24_P323997	TPRX1	0.0136	-1.67
A_32_P141135	TRAF3IP3	0.0135	2.57
A_23_P19333	TREM1	0.0240	-2.60
A_23_P7932	TREML2	0.0253	-1.61
A_24_P337062	TSC22D2	0.0382	-1.68
A_23_P121987	TSLP	0.0403	1.67
A_23_P29248	TST	0.0156	1.74
A_23_P230	TTC4	0.0486	-1.51
A_24_P359838	TTL	0.0000	-1.51
A_23_P500892	TUB	0.0119	2.08
A_24_P211064	TWSG1	0.0183	-2.13
A_23_P27256	TWSG1	0.0209	-1.96
A_23_P204581	TXNRD1	0.0236	-2.01
A_23_P6307	U2AF1	0.0467	1.52
A_23_P21207	UBE1L	0.0055	2.11
A_32_P155506	UBE2E2	0.0080	-1.93
A_24_P412617	UBE2Q2	0.0177	-1.71
A_23_P77274	UBE2Q2	0.0213	-1.56
A_24_P329597	UBQLN1	0.0086	-2.67
A_24_P102203	UBR1	0.0292	-1.55
A_23_P132956	UCHL1	0.0014	-2.34
A_23_P60599	UGT1A6	0.0402	2.14
A_32_P914221	USH1G	0.0130	1.76
A_32_P26330	USP10	0.0156	-1.57
A_32_P132206	USP18	0.0034	1.87
A_23_P132159	USP18	0.0036	1.85
A_23_P207666	USP6	0.0250	-1.51
A_23_P129695	VASN	0.0336	1.65
A_23_P201551	VAV3	0.0090	1.75
A_23_P256223	VBP1	0.0164	-1.88

A_23_P253123	VGLL1	0.0009	9.34
A_23_P161190	VIM	0.0423	-2.19
A_23_P130285	VPS53	0.0316	-1.61
A_23_P86089	VWA1	0.0231	1.54
A_23_P205255	WDR20	0.0427	-1.51
A_24_P248167	WDR21A	0.0059	1.60
A_23_P11787	WNT4	0.0121	5.10
A_23_P382607	WNT4	0.0433	2.22
A_23_P4353	WSB1	0.0288	1.62
A_23_P81392	WWC1	0.0273	1.61
A_32_P6868	WWTR1	0.0283	-2.05
A_24_P336417	XPR1	0.0042	-1.78
A_23_P139825	YAF2	0.0456	-1.68
A_24_P48403	YES1	0.0114	-1.77
A_23_P164507	YES1	0.0289	-2.81
A_24_P501544	YPEL2	0.0385	-2.00
A_32_P226149	YWHAZ	0.0018	-1.69
A_24_P209571	YWHAZ	0.0039	-1.61
A_23_P330448	ZADH2	0.0399	-1.54
A_23_P93032	ZBED3	0.0043	2.81
A_24_P76995	ZDHHC17	0.0429	-1.60
A_32_P226205	ZFHX2	0.0234	2.47
A_23_P388867	ZNF141	0.0037	-1.63
A_24_P402836	ZNF141	0.0135	-1.59
A_23_P404064	ZNF161	0.0376	-1.52
A_23_P66260	ZNF267	0.0142	-1.52
A_23_P258124	ZNF346	0.0115	1.71
A_23_P434430	ZNF439	0.0181	-2.00
A_23_P23966	ZNF488	0.0280	1.81
A_24_P185487	ZNF507	0.0421	-1.69
A_32_P187663	ZNF596	0.0053	-1.74
A_23_P355993	ZNF614	0.0420	-1.65
A_23_P5405	ZNF650	0.0040	-1.85
A_24_P285501	ZNF650	0.0133	-1.64
A_24_P71700	ZNF651	0.0339	1.53
A_24_P118862	ZNF678	0.0398	-1.51
A_32_P163458	tcag7.1017	0.0299	1.55
