# Supplementary material for: Genome-Wide Small RNA Sequencing and Gene Expression Analysis Reveals a microRNA Profile of Cancer Susceptibility in ATM-Deficient Human Mammary Epithelial Cells
Source: PLoS One. 2013 May 31;8(5):e64779. doi: 10.1371/journal.pone.0064779 (PMC3669333; doi:10.1371/journal.pone.0064779)
Supplement: Table S5 — 40 miRNA and 202 mRNA targets. List of 40 significantly regulated miRNAs with negatively correlated predicted mRNA targets and the 202 mRNA predicted targets with significant changes in gene expression. The direction of change (increase or decrease) of the ATM-deficient cells compared to the WT cells is indicated for both the miRNAs (column 2) and the gene (mRNA) targets (column 4). (PDF) [file pone.0064779.s005.pdf]

miRNA ID	Change in expression level after loss of ATM	Gene Symbol	Change in expression level after loss of ATM
hsa-miR-106a	Up	ABHD2, AHRR, B4GALT6, BAMBI, BMP2, C10orf46, CCNG2, CEP170, CNOT6L, CRIM1, DCBLD2, DPYSL2, EFNB2, FAM3C, FNDC3A, FRMD6, GDA, GPD2, HMGA2, KATNAL1, MASTL, MAT2B, MBD5, MBTPS1, MCL1, NAA30, OTUD4, PCDHA11, PLDN, PPP2CA, PPP3R1, RAB22A, RPS6KA2, SEMA4B, SLC30A7, SNX9, STYX, SULF1, TMEM123, TMEM64, TSC22D2, USP6, YES1, YPEL2	Down
hsa-miR-107	Up	ABHD2, ACTR2, BACH2,CAPZA2, CDC27, CHD2, CNOT6L, DCBLD2, EFNB2, EIF2C4, EXOC5, G3BP2, GK, GNAI3, KIAA1804, MCFD2, MTMR4, OTUD4, PPP3R1, PRMT8, RAB10, RAI14, SNRK, ST13, YAF2	Down
hsa-miR-10b	Up	BACH2, ELAVL2, GATA6, MAP4K4, SMAD2	Down
hsa-miR-1201	Up	CNOT6L, PLDN, SEMA4B, SRF	Down
hsa-miR-130a	Down	AGPAT3, CALB1, FBXO9, MAF, N4BP1, NAV2, NDRG2, SMOC1, TOM1L2	Up
hsa-miR-135b	Down	ABAT, FRMD4A, MEGF6	Up
hsa-miR-141	Down	FRMD4A, LOC162073, MAF, SCD5	Up
hsa-miR-148a/b	Up	ARFIP1, BACH2, CPD, EFNB2, EIF2C4, EIF4E3, ELAVL2, FBXO11, FMR1, HMGA2, ITGA5, KIAA0256, KIAA1324L, MAP1B, March3, MDFIC, MTMR6, MTMR9, NRP1, OTUD4, PIGA, PRKAG2, PTGES3, QKI, RA, 14, RBM9, ROCK1, SMAD2, SNRK, SULF1, USP6, WDR20	Down
hsa-miR-152	Up	ARFIP1, BACH2, CPD, EFNB2, EIF2C4, EIF4E3, ELAVL2, FBXO11, FMR1, HMGA2, ITGA5, KIAA0256, KIAA1324L, MAP1B, March3, MDFIC, MTMR6, MTMR9, NRP1, OTUD4, PIGA, PRKAG2, PTGES3, QKI, RAB14, RBM9, ROCK1, SMAD2, SNRK, SULF1, USP6, WDR20	Down
hsa-miR-183	Up	BACH2, BMI1, BZW1, CHD2, CNOT6L, CYYR1, FGF9, FRMD6, GPAM, ICK, MTMR6, MYO1B, NUDT4, OSBPL8, PPP2CA, YAF2	Down
hsa-miR-192	Up	PABPC4, PRKAR1A	Down
hsa-miR-19a	Down	ENC1, MICAL3, MYLIP, NBEAL2, PITX1, SLC37A1, SMOC1, TUB	Up
hsa-miR-200a	Down	FRMD4A, MAF, SCD5	Up
hsa-miR-200b	Up	AP1S2, BACH2, BMI1, C10orf46, C3orf23, C6orf167, CHD2, COPS8, DCBLD2, DDEF1, EFNB2, ELAVL2, EPS8, ETV5, FRMD6, GNAI3, HS2ST1, ICK, KATNAL1, KIAA0256, MAP4K4, MCFD2, NUDT4, OTUD4, PCTK2, PPP2CA, PPP2R2C, PRKAR1A, RAP1B, RBM9, RPS6KA2, SMAD2, SPAST, SRF, SULF1, TSC22D2, YPEL2	Down
hsa-miR-200c	Up	AP1AR, AP1S2, ASAP1, B4GALT6, BACH2, BMI1, C10orf46, C3orf23, CDK17, CHD2, CNOT6L, COPS8, DCBLD2, EFNB2, ELAVL2, EPS8, ETV5, FRMD6, GATA6, GNAI3, HS2ST1, ICK, KATNAL1, MAP4K4, MCFD2, MMS22L, MTMR6, MTMR9, NUDT4, OTUD4, PLDN, PPP2CA, PPP2R2C, PRKAR1A, QKI, RAP1B, RBFOX2, RPS6KA2, SMAD2, SPAST, SRF, SULF1, TMEM123, TSC22D2, YPEL2	Down
hsa-miR-215	Up	PABPC4	
hsa-miR-221	Up	BMI1,FMR1, FNDC3A, GNAI3, NFYB, PCDHA11, PPP3R1, RAB22A, SOCS1, STYX	Down
hsa-miR-29c	Down	BLMH, CBX6, COL5A3, FRMD4A, FZD5, IFI30, MEGF6, NAV2, PTRF, ZNF346	Up

hsa-miR-30a/d	Up	ABHD2, B4GALT6, BACH2, C9orf72, CAMK4, CEACAM1,CEP170, CYYR1, DPYSL2, ELAVL2, EPDR1, FAM83F, FNDC3A, GDA, GJA1, ICK, ITGA5, KIAA1804, LARP4, LIFR, LIN7C, MAP4K4, MYH10, NFYB, OTUD4, PIGA, PPP3R1, PRKAR1A, QKI, RAB22A, RAI14, RAP1B, RFFL, RPS6KA2, RUNX2, SMAD2, SNIP1, SOCS1, SPAST, STAG2, STXBP1, TFDP1, THBS2, TIA1, TMEFF1, XPR1, YAF2, YES1, YPEL2, YWHAZ	Down
hsa-miR-31	Down	CEBPA, DGCR8, PDAP1, SALL2, VAV3	Up
hsa-miR-335	Up	ACTR2, CRIM1, FMR1, MAT2B, MTMR9, NFYB, RBFOX2, SNIP1	Down
hsa-miR-33a/b	Down	ENC1, GALNT10	
hsa-miR-345	Down	SORT1	Up
hsa-miR-374a	Down	ACTR2, ADAM10, ANTXR2, BACH2, BMP2, C3orf23, CAPZA2, CDA, CDK17, CRIM1, CTAGE5, CYYR1, EPDR1, EXOC5, FGF5, FNDC3A, GK, ICK, LIFR, LIN7C, MCFD2, MME, MTMR9, NFYB, OSBPL8, PCDHA11, PLDN, PPP2CA, PPP2R2C, RAB10, RAB22A, SMAD2, STYX, TFDP1, TMEM123, WDR20, YAF2, YPEL2	Up
hsa-miR-379	Up	CAPZA2, KATNAL1	Down
hsa-miR-424	Down	AGPAT3, GYLTL1B, KIF5A, N4BP1, OSCP1, PDAP1, SEMA3D, SORT1, WNT4, WSB1	Up
hsa-miR-425	Down	CBX6, KIAA0889, SLC37A1	Up
hsa-miR-452	Up	B4GALT6, CHD2, CNOT6L, ELAVL2, EPS8, FNDC3A, KIAA1804, LIFR, MTMR6, RAB14, SMAD2, SNRK	Down
hsa-miR-548e	Up	B4GALT6, BZW1, CPD, EIF2C4, ELAVL2, EXOC5, FAM3C, FGF5, FMR1, FNDC3A, G3BP2, GDA, GJA1, GPAM, KRIT1, LIN7C, MSRB3, NIPA2, OSBPL8, PPP3R1, QKI, RAB10, RAB22A, STAG2, TMEM64, TXNRD1, YAF2, YES1	Down
hsa-miR-598	Up	MSRB3	Down
hsa-miR-625	Down	CBX6	
hsa-miR-651	Up	GDA, RAP1B, SLMAP, SPAST, STXBP1	Down
hsa-miR-708	Down	AGPAT3, DNAJA4, LYPD3, N4BP1, RGS16	Up
hsa-miR-93	Up	ABHD2, AHRR,AHRR, B4GALT6, BAMBI, BMP2, C10orf46, CCNG2, CEP170, CNOT6L, CRIM1, DCBLD2, DPYSL2, EFNB2, FAM3C, FNDC3A, FRMD6, GDA, GPD2, HMGA2, MASTL, MAT2B, MBD5, MBTPS1, MCL1, NAT12, OTUD4, PCDHA11, PPP2CA, PPP3R1, RAB22A, RPS6KA5, SEMA4B, SLC30A7, SNX9, STYX, SULF1, TMEM123, TMEM64, TSC22D2, USP6, YES1, YPEL2	
hsa-miR-944	Up	ACTR2, AHRR, DCBLD2, DPYSL2, EXOC5, FAM3C, GATA6, GK, GPAM, LIN7C, MDFIC, MMS22L, MTMR6, OSBPL8, PLDN, PPP2CA, PRKAG2, PRKAR1A, QKI, RAB22A, RUNX2, SLMAP, SMAD2, SPAST, STYX, TMEM64, TSC22D2, UBR1, YAF2, YPEL2	Down
hsa-miR-96	Down	ABAT, CBX6, CELSR2, FRMD4A, MAF, N4BP1, PODXL, SORT1	Up
hsa-miR-99b	Up	AP1AR, EPDR1, HS3ST2	Down
