# Supplementary material for: Genome-Wide Small RNA Sequencing and Gene Expression Analysis Reveals a microRNA Profile of Cancer Susceptibility in ATM-Deficient Human Mammary Epithelial Cells
Source: PLoS One. 2013 May 31;8(5):e64779. doi: 10.1371/journal.pone.0064779 (PMC3669333; doi:10.1371/journal.pone.0064779)
Supplement: Table S6 — Potential target transcription factors. Transcription Factors identified as possible targets of significantly regulated miRNAs using the Molecular Signatures Database of GSEA. (PDF) [file pone.0064779.s006.pdf]

Transcription Factors

CEBPA
CSRP1
ELF3
EYA2
GATA6
ID2
ID3
MAF
NCOA4
NR4A3
POU5F1
RRN3
SALL2
TAF1A
TCF7L1
TUB
VGLL1
YAF2
ZNF141
ZNF267
