# Supplementary material for: Genome-Wide Small RNA Sequencing and Gene Expression Analysis Reveals a microRNA Profile of Cancer Susceptibility in ATM-Deficient Human Mammary Epithelial Cells
Source: PLoS One. 2013 May 31;8(5):e64779. doi: 10.1371/journal.pone.0064779 (PMC3669333; doi:10.1371/journal.pone.0064779)
Supplement: Table S7 — Representative genes in specific functional categories. Examples of target genes implicated in Cancer, Cell Cycle Regulation and DNA Replication, Recombination and Repair based on Ingenuity Pathway Analysis. (PDF) [file pone.0064779.s007.pdf]

| Selected Genes Implicated in Cancer | Selected Genes Implicated in Cell Cycle |
| --- | --- |
| ABAT | BMI1 |
| ADAM10 | BMP2 |
| ANTXR2 | CACUL1 |
| BAMBI | CCNG2 |
| BZW1 | CDC27 |
| CAPZA2 | CEBPA |
| CDA | FGF9 |
| CDC27 | GATA6 |
| DGCR8 | GJA1 |
| DPYSL2 | GNA13 |
| EFNB2 | HMGA2 |
| ENC1 | ITGA5 |
| EPS8 | MASTL |
| ETV5 | NRP1 |
| GATA6 | PRKAR1A |
| GJA1 | PTGES3 |
| HMGA2 | RFFL |
| HS3ST2 | RUNX2 |
| IFI30 | SMAD2 |
| LIFR | SOCS1 |
| LIN7C | SULF1 |
| MAF | TFDP1 |
| MAP4K4 | VAV3 |
| MCL1 | WNT4 |
| MME | ZNF346 |
| MTMR6 | |
| NRP1 | |
| PPP2CA | |
| RUNX2 | |
| SMAD2 | |
| SOCS1 | |
| SORT1 | |
| SULF1 | |
| VAV3 | |
| VIM | |
| YES1 | |
| YWHAZ | |

**Selected Genes Implicated in DNA Replication, Recombination and Repair**

BMP2
CEBPA
CEBPA
FGF9
GJA1
GNAI3
ITGA5
MAF
MCL1
RAP1B
RUNX2
SALL2
THBS2
VIM
